# Synthesis of Methyl Aprabiosaminide and 2-Hydroxyapramycin from Apramycin

**DOI:** 10.1021/acs.orglett.5c00168

**Published:** 2025-02-14

**Authors:** Niteshlal Kasdekar, Michael R. Spieker, Andrea Vasella, Sven N. Hobbie, David Crich

**Affiliations:** † Department of Pharmaceutical and Biomedical Sciences, University of Georgia, 250 West Green Street, Athens, Georgia 30602, United States; ‡ Department of Biochemistry and Molecular Biology, University of Georgia, 120 East Green Street, Athens, Georgia 30602, United States; § Organic Chemistry Laboratory, ETH Zürich, Vladimir-Prelog-Weg 1-5/10, 8093 Zürich, Switzerland; ∥ Division of Clinical Bacteriology and Mycology, University Hospital Basel, Petersgraben 4, 4031 Basel, Switzerland; ⊥ Department of Chemistry, University of Georgia, 302 East Campus Road, Athens, Georgia 30602, United States; # Complex Carbohydrate Research Center, University of Georgia, 315 Riverbend Road, Athens, Georgia 30602, United States

## Abstract

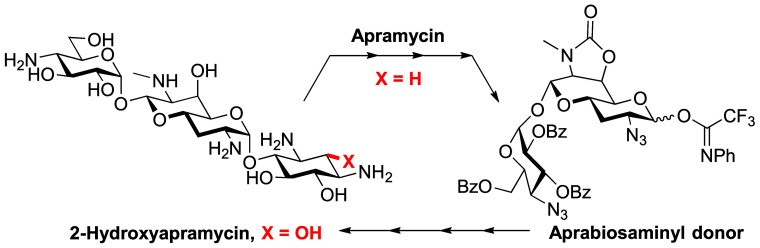

We describe a protocol for the selective cleavage of the 2-deoxystreptamine ring from the structurally unusual aminoglycoside antibiotic apramycin, enabling for the first time the preparation of aprabiosamine derivatives. We further describe reglycosylation of the aprabiosamine core with a selectively protected optically pure streptamine derivative, giving, after deprotection, 2-hydroxyapramycin, the first apramycin derivative functionalized at the 2 position.

Apramycin **1**(1) is an atypical 2-deoxystreptamine (DOS)-type aminoglycoside antibiotic (AGA)^2−5^ lacking substitution at either the 5 or 6 positions, whose unusual structure circumvents the action of the vast majority of resistance-imparting aminoglycoside-modifying enzymes (AMEs) and ribosomal methyltransferases (RMTs), all while conveying minimal ototoxicity,^6−9^ culminating in its recent assessment in phase 1 clinical trials (NCT04105205 and NCT05590728; www.clinicaltrials.gov). These attributes and the ever-growing threat of multidrug-resistant (MDR) infections^10^ have spurred multiple efforts to synthesize next-generation derivatives of apramycin with greater levels of activity and the ability to overcome the one remaining AME that acts on it,^11−13^ the still rare but spreading aminoacyltransferase (3)-IV [AAC(3)-IV].^6,7,14−17^

We report here on the first synthesis and evaluation of an apramycin derivative modified at the 2 position, 2-hydroxyapramycin **2**. The selection of compound **2** as a target builds on the expected reduction of ∼1 p*K*_a_ unit in the basicity of N3^18^ and the anticipated corresponding reduction in its nucleophilicity, which we anticipated to retard the acetyl group transfer by AAC(3)-IV. We were further inspired by the hybrimycins **3**–**5**,^19−24^ 2-hydroxy analogues of the 4,5-disubstituted DOS AGAs paromomycin **6** and neomycin **7**, especially those in which the additional hydroxy group is equatorial, and by the 2-hydroxy analogues **8** and **9**, respectively, of the clinically significant 4,6-disubstituted DOS AGAs gentamicin C1a **10**(25) and arbekacin **11**,^26^ which retain high levels of antibacterial activity coupled with reduced nephrotoxicity (Figure 1).

**Figure 1:**
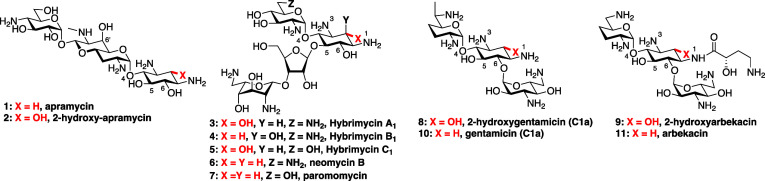
2-Deoxystreptamine AGAs and their 2-hydroxy variants.

We selected a route based on cleavage of the DOS ring from compound **1** to give a derivative of previously unknown aprabiosamine **12** and reglycosylation with a suitably desymmetrized derivative of streptamine **13** (Scheme 1).^27^ This route held the added attraction of opening the way to the eventual synthesis of a broader variety of ring I analogues, such as explored in other AGA classes.^28^ Aqueous acidic hydrolysis of apramycin selectively cleaves the 4-aminoglucosyl ring to yield the far less active aprosamine **14**, with more forcing conditions required to cleave the DOS ring **15**.^1^ This degradation sequence is maintained in protected variants of apramycin,^13,29^ such that aprabiosamine and its derivatives were previously unknown. Furthermore, total syntheses of apramycin and its analogues have all proceeded from 2-deoxystreptamine derivatives as starting material,^30−33^ such that all known derivatives of apramycin at the beginning of this project were characterized by the absence of functionality at the 2 position.

**Scheme 1:**
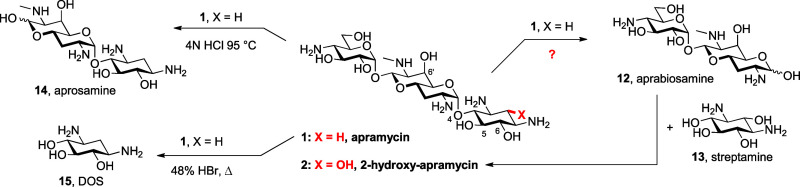
Acid-Mediated Degradation of Apramycin **1** and the Strategic Disconnection to Aprabiosamine, Enabling Hemisynthesis of 2-Hydroxyapramycin **2**

Apramycin **1** was converted to tetraazido triazene **16** by reaction first with benzenediazonium tetrafluoroborate and then imidazolesulfonyl azide as previously described.^34^ The reaction of compound **16** with benzoyl chloride in pyridine gave pentabenzoate **17** as a white crystalline solid in 58% yield, in which the regioselective monobenzoylation of the 5,6-diol moiety is consistent with earlier reports on selective protection of neamine derivatives and other apramycin derivatives.^8,11,15,35^ Exposure of compound **17** to cesium carbonate in *N*,*N*-dimethylformamide (DMF) at room temperature resulted in a 6 → 5-benzoyl group migration,^36^ consistent with earlier observations,^11^ giving an approximately 1:1 separable mixture of the two regioisomers **17** and **18**. Dess–Martin oxidation of 5-*O*-benzoate **18** gave corresponding ketone **19** that on treatment with lithium carbonate in DMF underwent β-elimination, yielding a first aprabiosamine derivative **20** in 68% yield. Treatment with *N*-phenyl-trifluoroacetimidoyl chloride^37,38^ and cesium carbonate gave the corresponding glycosyl donor **21** as a 2.7:1 α/β mixture of anomers in 88% yield (Scheme 2). Unfortunately, attempted application of compound **21** as a donor for the glycosylation of enantiomerically pure streptamine derivative **22**, obtained from streptomycin,^39^ resulted in only degradation under the various conditions investigated as a result of the sensitivity of the triazene moiety toward Brønsted and Lewis acidic conditions (Scheme 2).

**Scheme 2:**
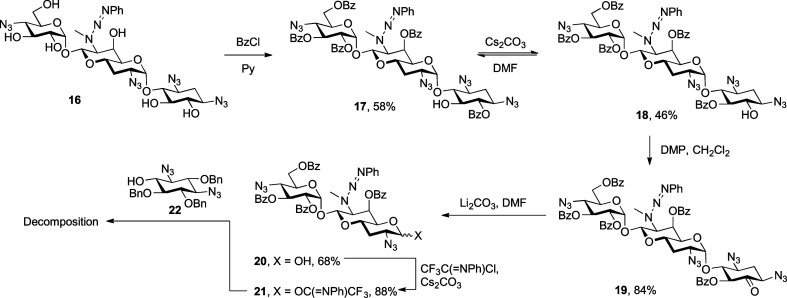
Synthesis and Attempted Glycosylation of Triazene-Protected Aprabiosaminide Donor **21**

We fell back on the more robust 6′-*O*,7′-*N*-oxazolidine protection of the secondary amine in apramycin, preparing compound **23** and subjecting it to regioselective tetrabenzoylation to give compound **24** in 82% yield as previously described.^11^ Treatment of compound **24** with cesium carbonate in DMF gave an inseparable mixture of 6- and 5-*O*-benzoates **24** and **25**, which was subjected to Dess–Martin oxidation to give ketones **26** and **27** in 40 and 46% yields, respectively. The latter was subjected to elimination with lithium carbonate in DMF to give aprabiosamine derivative **28** in 79% yield. A minor phenolic glycoside **29**, arising from 5 → 6-benzoyl migration at the enolate stage, subsequent elimination of two azido groups, and aromatization, was also isolated in the form of a mixture of regioisomers. Treatment of ketone **26** under similar conditions also yielded a regioisomeric mixture of the phenolic glycosides **29** in 86% yield, which was converted to perbenzoate derivative **30** for ease of characterization (Scheme 3).

**Scheme 3:**
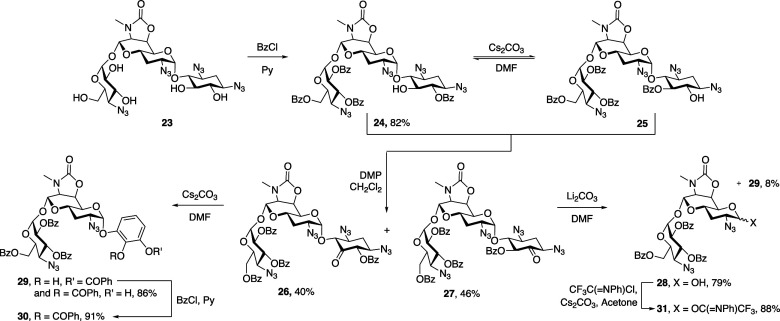
Preparation of *N*-Phenyltrifluoromethylimidate **31** and Phenolic Glycosides **29** and **30**

The ^1^H nuclear magnetic resonance (NMR) spectrum of aprabiose hemiacetal **28** was characterized by an unusual upfield δ_H1_ value of the β-anomer at 3.36 ppm (^3^*J*_H1,H2_ = 7.3 Hz), while corresponding anomeric carbon had a more typical δ_C1_ value of 98.3 ppm. The α-anomer of compound **28** had a typical anomeric δ_H1_ of 5.07 ppm (^3^*J*_H1,H2_ = 3.4 Hz). We traced this phenomenon to the adoption of a boat–chair conformation by the bicyclic core of compound **28β**, enforced by the *cis*-fused oxazolidinone ring and indicated by the large ^3^*J*_H6,H7_ coupling constant of 9.0 Hz and the small ^3^*J*_H7,H8_ coupling constant of 2.2 Hz. This conformation places H1 in the shielding cone of 2′-*O*-benzoate ester of the terminal 4-aminoglucosyl ring (Figure 2). Hemiacetal **28** was converted in 88% yield to *N*-phenyltrifluoroacetimate **31**, an α/β mixture in which H1 of the β-anomer also had a somewhat upfield δ_H1_ of 4.70 ppm.

**Figure 2:**
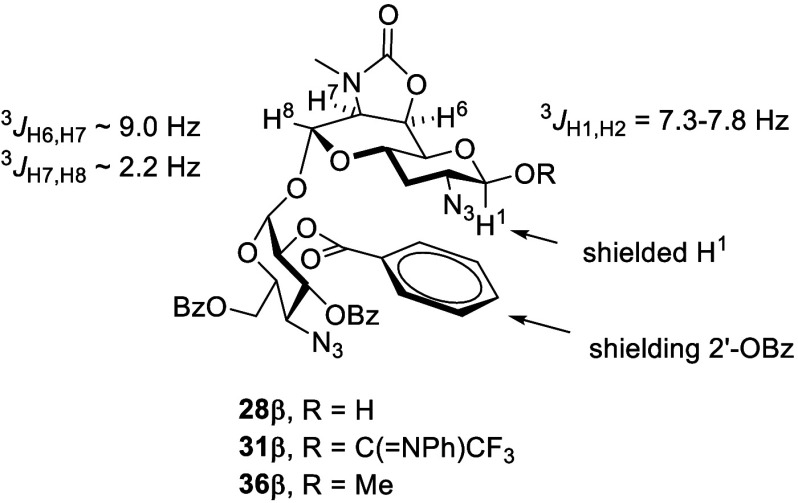
Conformation of the 2′-*O*-benzoyl β-aprabiosaminide derivatives **28β**, **31β**, and **36β**.

Reaction of compound **31** with acceptor **22** in the presence of TfOH as a promoter resulted in the formation of an inseparable mixture of anomeric glycosides **32** in 68% yield, unexpectedly favoring the equatorial isomer. Benzoate deprotection under Zemplen conditions then gave equatorial and axial glycosides **33** and **34**. Lacking 2′-*O*-benzoate ester and its shielding effect, equatorial glycoside **33** has a more typical anomeric δ_H1_ of 4.92 ppm (^3^*J*_H1,H2_ = 7.9 Hz). Coupling of donor **31** with methanol similarly gave a 1:1.4 α,β-anomeric mixture of methyl glycosides **36** favoring the equatorial β-isomer, which also exhibited an unusual upfield δ_H1_ at 2.95 ppm (^3^*J*_H1,H2_ = 7.8 Hz). Despite considerable effort, we have thus far been unable to overcome the preference of donor **31** for β-selective glycosylation, which we attribute to shielding of the α-face by the terminal aminoglucosyl ring, consistent with upfield δ_H1_ in the various 2′-*O*-benzoyl equatorial anomers **28β**, **31β**, and **36β**. Deprotection of compound **34**, its equatorial β-isomer **33**, and the mixture of methyl glycosides **36** finally gave the target compound 2-hydroxyapramycin **2** in 21% yield, its 1′-epimer **35** in 39% yield, and methyl aprabiosamide **37** as a 1:1.2 mixture of anomers in 74% yield (Scheme 4).

**Scheme 4:**
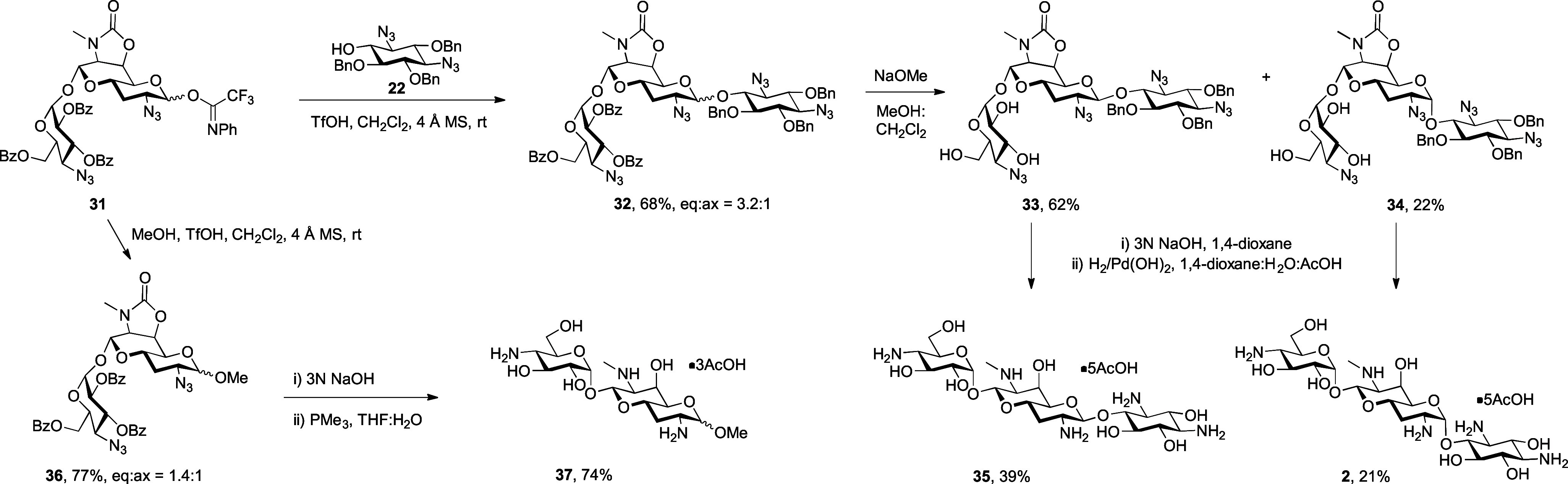
Synthesis of 2-Hydroxyapramycin **2**, 2-Hydroxy-1′-epiapramycin **35**, and Methyl α,β-Aprabiosaminide **37**

2-Hydroxyapramycin **2**, its 1′-epimer **35**, and the mixture of methyl aprabiosamides **37** were screened along with the parent apramycin **1** for their ability to inhibit protein synthesis in a cell-free translation assay employing wild-type bacterial ribosomes and for their ability to inhibit growth of wild-type Gram-negative *Escherichia coli* and Gram-positive methicillin-resistant *Staphylococcus aureus* (MRSA) (Table 1). Disappointingly, 2-hydroxyapramycin **2** was approximately 10-fold less active in the cell-free translation assay than apramycin itself, reflecting a negative impact of the 2-hydroxy group on binding to the drug binding pocket, the decoding A site.^7^ The 1′-epimer **35** was essentially devoid of activity, as was the methyl aprabiosamide anomeric mixture, reflecting both the importance of the (deoxy) streptamine ring for the inhibition of bacterial protein synthesis and its axial linkage to the bicyclic octadiose core of the molecule. Minimum inhibitory concentrations (MICs) against both *E. coli* and MRSA confirmed the trend in the IC_50_ values with 2-hydroxyapramycin being much less active than apramycin itself and compounds **35** and **37** essentially inactive. In view of the low activity of 2-hydroxyapramycin, broader antibacterial screening was foregone.

**Table 1:** Inhibition of Protein Synthesis by Wild-Type Bacterial Ribosomes (IC_50_) and MICs

		MIC (μg/mL) (strain)	
compound	IC_50_ (μM)	*E. coli* (ATCC 25922)	MRSA (AG038)
**1**	0.14	4	4
**2**	1.1	64	>64
**31**	>20	>256	>128
**32**	>20	>256	>256

The loss of activity on installation of the 2-hydroxy group in apramycin stands in contrast that of the same modification to neomycin and paromomycin in the form of hybrimycins, gentamicin, and arbekacin (Figure 1)^19−26^ but is consistent with the reduced activity of 2-hydroxyribostamycin **38** in comparison to parent ribostamycin **39** (Figure 3).^23^ We conclude that, for the 4,5-DOS AGAs neomycin and paromomycin, the reduction in basicity of N1 and N3 on hydroxylation and the subsequent reduction in affinity for the drug binding pocket is adequately compensated by the presence of the doubly positively charged ring IV and the strong electrostatic attraction that it provides for the ribosome:^40^ in contrast in apramycin and ribostamycin, lacking ring IV, hydroxylation at the 2 position results in a significant loss of affinity for the binding pocket and a concomitant reduction in activity.

**Figure 3:**
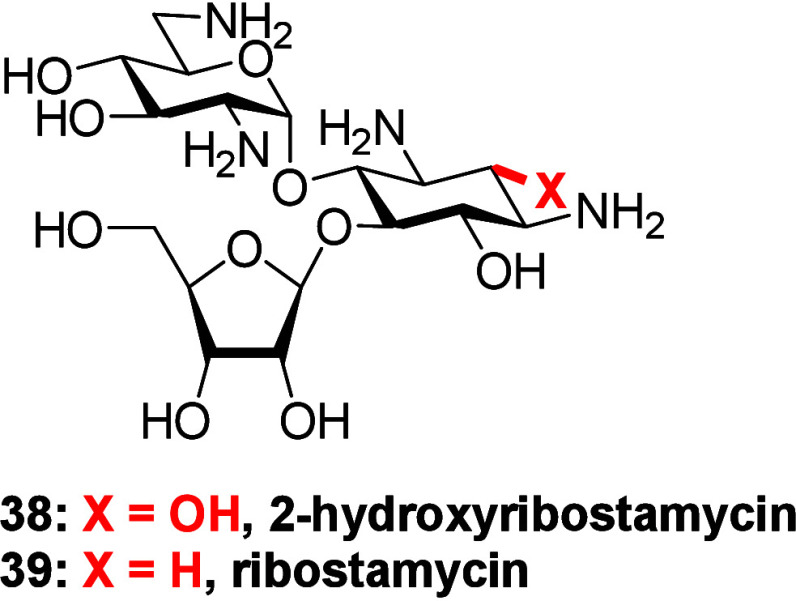
2-Hydroxyribostamycin and ribostamycin.

## Data Availability

The data underlying this study are available in the published article and its Supporting Information.

## Supporting Information Available

Full experimental details and copies of NMR spectra of all compounds (PDF)
