# Supplementary material for: Synthesis of Methyl Aprabiosaminide and 2-Hydroxyapramycin from Apramycin
Source: Org Lett. 2025 Feb 14;27(8):1918–22. doi: 10.1021/acs.orglett.5c00168 (PMC11877507; doi:10.1021/acs.orglett.5c00168)

Supporting information

for

Synthesis of Methyl Aprabiosaminide and 2-Hydroxyapramycin from Apramycin

Niteshlal Kasdekar,[a] Michael R Spieker,[a,b] Andrea Vasella,[c] Sven N. Hobbie,[d] and David Crich[a,e,f,*]

a) Department of Pharmaceutical and Biomedical Sciences, University of Georgia, 250 West Green Street, Athens, GA 30602, USA

b) Department of Biochemistry and Molecular Biology, University of Georgia, 120 East Green Street, Athens, GA 30602, USA

c) Organic Chemistry Laboratory, ETH Zürich, Vladimir-Prelog-Weg 1-5/10, 8093 Zürich, Switzerland

d) Division of Clinical Bacteriology and Mycology, University Hospital Basel, Petersgraben 4, 4031 Basel, Switzerland

e) Department of Chemistry, University of Georgia, 302 East Campus Road, Athens, GA 30602, USA

f) Complex Carbohydrate Research Center, University of Georgia, 315 Riverbend Road, Athens, GA 30602, USA

Email: David.Crich@uga.edu

Table of contents

**Contents**	**Page no.**
General experimental	S3
Caution	S3
Experimental procedure and compound characterization data	S3 – S19
Cell-free translation assays	S20
Antibacterial assays	S20
References	S21
Spectral charts	S22-S93

Experimental Section

General Experimental. All reagents were purchased from commercial sources and used without further purification. Thin-layer chromatography (TLC) was carried out with 250 μm glass-backed silica plates. TLC plates were visualized by UV irradiation (254 nm) and by charring with sulfuric acid in ethanol (20:80, v/v) or with ceric ammonium molybdate solution [$Ce(SO_4)_2$: 4 g, $(NH_4)_6Mo_7O_{24}$: 10 g, H_2SO_4: 40 mL, H_2O: 360 mL]. All organic solutions were concentrated under vacuum at 30 - 45 ºC on a rotary evaporator. The purification of crude residues was carried over silica gel (230-400 mesh) using automated flash column chromatography. Specific rotations were recorded on an automatic polarimeter in $CHCl_3$, MeOH, or H_2O at 589 nm and 21 ± 1 ºC with a path length of 10 cm. Nuclear magnetic resonance (NMR) spectra of all compounds were obtained in $CDCl_3$, CD_3OD, or D_2O on 500, 600 or 900 MHz instruments, with chemical shifts (δ) calculated with respect to the residual solvent peak and given in ppm. NMR multiplicities are abbreviated as follows: s (singlet), d (doublet), t (triplet), q (quartet), dd (doublet of doublet), br s (broad singlet) and m (multiplet). Peak assignments are based on COSY, HSQC, HMBC and TOCSY experiments. High-resolution electrospray ionization (ESI) mass spectrometry spectra were recorded using a Thermo Scientific Orbitrap mass analyzer. Reaction mixtures were heated when neccessary with an appropriately-sized, thermostatically-controlled aluminum heating block.

Caution. All reactions involving azides should be conducted in a fume hood behind an explosion shield.

1,3,2’,4’’-Tetra-azido-6,6’,2’’,3’’,6’’-penta-*O*-benzoyl-1,3,2’,4’’-tetra-(desamino)-7’-*N*-phenyldiazenyl-apramycin (17): To an ice-cold solution of compound **16** (0.6 g, 0.81 mmol) in anhydrous pyridine (8.0 mL), benzoyl chloride (0.56 mL, 4.81 mmol) was added dropwise over 10 mins after which the reaction mixture was stirred at room temperature overnight. After 14 h, the mixture was poured onto crushed ice, forming a slurry, which was extracted with ethyl acetate (30 mL × 3) and the combined organic layers were washed with brine (40 mL), dried over anhydrous

Na_2SO_4, and concentrated under reduced pressure. The crude product was purified by silica gel column chromatography using ethyl acetate in hexane (gradient 10 → 40%) to obtain the title compound **17** (0.60 g, 58%) as a white solid; R_f = 0.35 (40% EtOAc in hexane); $[\alpha]_D^{21}$ = (+)-58.1 (*c* = 1.0, $CHCl_3$); ^1H NMR (500 MHz, $CDCl_3$) δ 8.19 – 7.96 (m, 8H, Ar), 7.90 – 7.86 (m, 1H, Ar), 7.82 – 7.65 (m, 2H, Ar), 7.61 – 7.51 (m, 5H, Ar), 7.49 – 7.38 (m, 8H, Ar), 7.30 – 7.26 (m, 2H, Ar), 7.13 – 6.98 (m, 4H, Ar), 5.99 (t, *J* = 2.7 Hz, 1H, H6'), 5.89 (t, *J* = 10.0 Hz, 1H, H3"), 5.79 (d, *J* = 8.2 Hz, 1H, H8'), 5.75 (d, *J* = 3.6 Hz, 1H, H1"), 5.20 (d, *J* = 3.6 Hz, 1H, H1'), 5.19 – 5.09 (m, 2H, H2", H6), 3.99 (dd, *J* = 9.8, 2.7 Hz, 1H, H7'), 3.95 – 3.88 (m, 3H, 2 x H6", H5'), 3.87 (td, *J* = 10.6, 4.3 Hz, 1H, H4'), 3.80 – 3.72 (m, 2H, H5, H4"), 3.70 (dt, *J* = 10.1, 2.3 Hz, 1H, H5"), 3.55 (t, *J* = 9.4 Hz, 1H, H4), 3.43 (td, *J* = 11.2, 4.4 Hz, 1H, H3), 3.30 (s, 3H, N-Me), 3.30 – 3.26 (m, 1H, H2'), 2.46 (dt, *J* = 12.5, 4.5 Hz, 1H, H2a), 1.84 (dd, *J* = 11.0, 5.5 Hz, 1H, H3'a), 1.71 (q, *J* = 12.8 Hz, 1H, H2b), 1.48 (q, *J* = 12.0 Hz, 1H, H3'b); ^{13}C NMR (126 MHz, $CDCl_3$) δ 166.3 (C=O), 166.1 (C=O), 165.8 (C=O), 165.7 (C=O), 165.2 (C=O), 150.1, 134.7, 133.8, 133.7, 133.6, 133.4, 130.7, 130.2, 130.1, 129.9, 129.9, 129.8, 129.4, 129.3, 129.2, 129.1, 129.0, 128.7, 128.7, 128.6, 128.5, 128.4, 126.2, 120.8, 98.5 (C1'), 96.8 (C8'), 94.5 (C1''), 82.7 (C4), 75.9 (C2''), 75.1 (C5), 71.6 (C6), 70.5 (C3''), 69.3 (C7'), 68.8 (C5''), 68.7 (C6''), 67.4 (C5'), 66.7 (C4''), 62.1 (C6''), 60.4 (C4'), 59.3 (C3), 58.4 (C1), 57.1 (C2''), 36.5 (NCH_3), 32.4 (C2), 28.2 (C3'); HRMS (ESI) m/z calculated for $C_{62}H_{57}O_{16}N_{15}$ $[M + Na]^+$ 1290.4005; found 1290.4018.

1,3,2',4''-Tetra-azido-5,6',2'',3'',6''-penta-*O*-benzoyl-1,3,2',4''-tetra-(desamino)-7'-*N*-phenyldiazenyl-apramycin (18): To a stirred solution of compound **17** (0.66 g, 0.520 mmol) in anhydrous DMF (13.0 mL), Cs_2CO_3 (0.51 g, 1.560 mmol) was added after which stirring was continued for 18 h at room temperature before the mixture was diluted with ethyl acetate (40 mL) and washed with deionized water (30 mL). The aqueous layer was re-extracted with ethyl acetate (30 mL), and the combined organic layer was washed with brine (30 mL), dried over Na_2SO_4, and concentrated under reduced pressure. The crude mixture was purified by silica gel column

chromatography (eluent 20→40% EtOAc in hexane) giving compound **18** (0.31 g, 46%) as a white solid along with recovered **17** (0.32 g, 46%); R_f = 0.30 in 40% EtOAc in hexane; $[\alpha]_D^{21}$ = (+)-56.4 (*c* = 1.0, $CHCl_3$); ^{1}H NMR (500 MHz, $CDCl_3$) δ 8.04 – 7.93 (m, 8H, Ar), 7.87 (d, *J* = 7.7 Hz, 2H, Ar), 7.61 – 7.50 (m, 4H, Ar), 7.49 – 7.35 (m, 10H, Ar), 7.30 – 7.24 (m, 2H, Ar), 7.17 – 6.99 (m, 4H, Ar), 6.00 (t, *J* = 2.7 Hz, 1H, H6'), 5.89 (t, *J* = 10.0 Hz, 1H, H3''), 5.76 (d, *J* = 8.2 Hz, 1H, H8'), 5.73 (d, *J* = 3.6 Hz, 1H, H1''), 5.33 (t, *J* = 9.3 Hz, 1H, H5), 5.11 (dd, *J* = 10.2, 3.6 Hz, 1H, H2''), 4.93 (d, *J* = 3.6 Hz, 1H, H1'), 4.06 (dd, *J* = 9.9, 2.8 Hz, 1H, H5'), 3.96 – 3.89 (m, 3H, H6'', H7'), 3.82 – 3.75 (m, 3H, H4, H4', H4''), 3.70 (t, *J* = 9.3 Hz,, 1H, H6), 3.61 – 3.49 (m, 3H, H1, H3, H5''), 3.31 (s, 3H, NCH_3), 3.07 (s, 1H, OH), 2.74 (dt, *J* = 13.1, 4.1 Hz, 1H, H2'), 2.41 (dq, *J* = 7.8, 5.3 Hz, 1H, H2a), 1.69 (dt, *J* = 9.6, 4.5 Hz, 1H, H3'a), 1.58 (q, *J* = 12.4 Hz, 1H, H2b), 1.40 (q, *J* = 12.0 Hz, 1H, H3'b); ^{13}C NMR (126 MHz, $CDCl_3$) δ 166.2 (C=O), 166.2 (C=O), 165.9 (C=O), 165.7 (C=O), 165.3 (C=O), 150.1, 133.8, 133.6, 133.5, 133.4, 133.4, 130.1, 130.0, 129.9, 129.9, 129.8, 129.8, 129.4, 129.2, 129.2, 129.1, 128.8, 128.7, 128.7, 128.6, 128.5, 128.4, 126.2, 120.8 (aromatic), 98.5 (C1'), 96.9 (C8'), 94.6 (C1''), 78.1 (C4), 77.4 (C5), 76.5 (C5''), 75.5 (C2''), 71.4 (C3''), 70.4 (C6), 69.3 (C6'), 68.8 (C5'), 67.5 (C7'), 66.7 (C4'), 62.1 (C6''), 60.3 (C4''), 60.3 (C1) 59.3 (C3), 55.6 (C2'), 36.4 (NCH_3), 31.9 (C2), 27.6 (C3'); HRMS (ESI) m/z calculated for $C_{62}H_{57}O_{16}N_{15}$ $[M + Na]^+$ 1290.4005; found 1290.4025.

1,3,2',4''-Tetra-azido-5,6',2'',3'',6''-penta-*O*-benzoyl-1,3,2',4''-tetra-(desamino)-7'-*N*-phenyldiazenyl-apramycin-6-one (19): Dess-martin periodinane (300 mg, 0.77 mmol) was added to the stirred solution of 5-*O*-benzoate **18** (300 mg, 0.236 mmol) in an anhydrous CH_2Cl_2 (6.0 mL), and the mixture was stirred for 18 h. Then, the mixture was diluted with CH_2Cl_2 (30 mL), washed with deionized water (40 mL), and brine (40 mL), dried over Na_2SO_4, concentrated under a vacuum, and the residue was subjected to silica gel column chromatography to isolate ketone **19** (253 mg, 84%) as a white amorphous solid; R_f = 0.4 in 35% EtOAc in hexanes; $[\alpha]_D^{21}$ = (+)-67.1 (*c* = 1.0, $CHCl_3$); ^{1}H NMR (500 MHz, $CDCl_3$) δ 8.11 – 7.95 (m, 8H, Ar), 7.89 – 7.87 (m, 2H,

Ar), 7.62 – 7.51 (m, 4H, Ar), 7.50 – 7.37 (m, 10H, Ar), 7.30 – 7.26 (m, 2H, Ar), 7.13 – 7.02 (m, 4H, Ar), 5.97 (d, *J* = 3.5 Hz, 1H, H6'), 5.89 (t, *J* = 10.0 Hz, 1H, H3''), 5.79 (d, *J* = 8.2 Hz, 1H, H8'), 5.75 (d, *J* = 3.6 Hz, 1H, H1''), 5.16 (d, *J* = 9.9 Hz, 1H, H5), 5.10 (dd, *J* = 10.3, 3.7 Hz, 1H, H2''), 5.07 (d, *J* = 3.5 Hz, 1H, H1'), 4.27 (dd, *J* = 12.6, 6.1 Hz, 1H, H2'), 4.06 – 4.01 (m, 2H, H6''), 4.00 – 3.95 (m, 1H, H5''), 3.94 – 3.91 (m, 2H, H4', H5'), 3.90 – 3.87 (m, 2H, H1, H7'), 3.84 (dt, *J* = 9.7, 4.0 Hz, 1H, H3), 3.77 (t, *J* = 10.1 Hz, 1H, H4), 3.69 (t, *J* = 10.6 Hz, 1H, H4''), 3.31 (s, 3H, NCH_3), 2.89 (dt, *J* = 12.1, 4.1 Hz, 1H, H2a), 2.64 (dt, *J* = 12.4, 5.4 Hz, 1H, H3'a), 1.81 (q, *J* = 12.2 Hz, 1H, H2b), 1.45 (q, *J* = 12.4 Hz, 1H, H3'b); ^{13}C NMR (125 MHz, $CDCl_3$) δ 194.5 (C=O), 165.5 (C=O), 165.3 (C=O) 165.0 (C=O), 164.4 (C=O), 164.2 (C=O), 153.0, 133.2, 133.1, 133.0, 132.9, 129.3, 129.2, 129.1, 129.0, 129.0, 128.6, 128.2, 128.1, 128.1, 128.0, 128.0, 127.9, 127.8, 127.7, 101.0 (C1'), 95.9 (C8'), 95.4 (C1''), 79.4 (C5), 78.7 (C4), 71.6 (C2''), 70.5 (C3''), 69.7 (C6'), 69.3 (C5''), 66.3 (C5'), 65.1 (C6''), 62.7 (C4'), 60.1 (C1), 59.5 (C4''), 59.0 (C3), 57.5 (C7'), 54.5 (C2'), 31.3 (C2), 29.7 (NCH_3), 26.7 (C3'); HRMS (ESI) *m/z* calculated for $C_{62}H_{55}N_{15}O_{16}$ $[M + Na]^+$ 1288.3849; found 1288.3851.

2,4'-Di-azido-6,2',3',6'-tetra-*O*-benzoyl-2,4'-di-(desamino)-7'-*N*-phenyldiazenyl-α,β-aprabiosamine (20): Ketone **19** (253 mg, 0.199 mmol) in anhydrous DMF (5.0 mL) was stirred with Li_2CO_3 (44 mg, 0.599 mmol) under an argon atmosphere. After 16 h, the reaction mixture was neutralized with glacial acetic acid (100 μL), diluted with ethyl acetate (40 mL), and washed with deionized water (50 mL). The aqueous layer was extracted with ethyl acetate (40 mL), and the combined organic layers were washed with brine (50 mL), dried over Na_2SO_4, and concentrated under vacuum. The crude mixture was purified by silica gel column chromatography, eluting with ethyl acetate in hexanes (20→40%), to isolate an anomeric mixture of hemiacetal **20** (132 mg, 68%) as a white amorphous solid. R_f = 0.30 (35% EtOAc in hexanes, data for both anomers were obtained with the aid of 1D-TOCSY Experiments). ^1H NMR (600 MHz, $CDCl_3$) δ 8.04 – 7.98 (m, 10H, Ar), 7.89 (d, *J* = 7.5 Hz, 2H, Ar), 7.61 – 7.51 (m, 6H, Ar), 7.49 – 7.37 (m,

10H, Ar), 7.28 (t, *J* = 7.8 Hz, 2H, Ar), 7.16 – 7.05 (m, 6H, Ar) (**β-anomer**) ^{1}H NMR (600 MHz, $CDCl_3$) δ 6.09 (t, *J* = 2.8 Hz, 1H, H6), 5.86 (t, *J* = 10.0 Hz, H2'), 5.79 (d, *J* = 8.2 Hz, 1H, H8), 5.74 (d, *J* = 3.7 Hz, 1H, H1'), 5.08 (d, *J* = 3.8 Hz, 1H, H1'), 4.57 (d, *J* = 7.7 Hz, 1H, H1), 3.94 – 3.89 (m, 2H, H6'), 3.92 (dd, *J* = 8.1, 2.6 Hz, 1H, H7), 3.86 (dt, *J* = 11.5, 4.5 Hz, 1H, H4), 3.85 – 3.82 (m, 1H, H5'), 3.76 – 3.73 (m, 1H, H4') 3.38 (dd, *J* = 9.6, 2.8 Hz, 1H,. H5), 3.19 (dt, *J* = 12.6, 6.6 Hz, 1H, H2), 2.03 – 1.91 (m, 1H, H3a), 0.93 (q, *J* = 12.3 Hz, 1H, H3b); (**α-anomer**) δ 5.96 (t, *J* = 2.8 Hz, 1H, H6), 5.89 (t, *J* = 10.0 Hz, 1H, H3'), 5.78 (d, *J* = 3.7 Hz, 1H, H1'), 5.61 (d, *J* = 9.8 Hz, 1H, H8), 5.14 (m, 1H, H2') 5.12 (s, 1H, H1), 3.98 (dd, *J* = 8.2, 2.5 Hz, 1H, H5) 3.95 (dd, *J* = 9.9, 2.8 Hz, 1H, H7), 3.94 – 3.89 (m, 2H, H6'), 3.78 (t, *J* = 9.5 Hz, 1H, H4'), 3.82 (td, *J* = 10.7, 4.3 Hz, 1H, H4), 3.72 – 3.71 (m, 1H, H5') 3.03 (dt, *J* = 12.7, 3.8 Hz, 1H, H2), 1.80 (t, *J* = 6.1 Hz, 1H, H3a), 1.48 (q, *J* = 12.0 Hz, 1H, H3b); ^{13}C NMR (151 MHz, $CDCl_3$) δ 166.2 (C=O), 166.0 (C=O), 165.9 (C=O), 165.7 (C=O), 165.7 (C=O), 165.5 (C=O), 150.13 (NC=O), 150.07 (NC=O), 133.8, 133.7, 133.6, 133.49, 133.4, 130.1, 130.1, 130.0, 130.0, 129.9, 129.92, 129.8, 129.7, 129.2, 129.2, 129.1, 129.1, 128.8, 128.7, 128.7, 128.6, 128.7, 128.6, 128.6, 128.5, 128.5, 126.3, 120.9, 120.8, 98.5 (C1β), 96.9(C8α), 96.5 (C8β), 94.5 (C1'α), 94.2 (C1'β), 92.0 (C1α), 72.6 (C2'β), 71.6 (C3'β), 71.5 (C2'α), 70.5 (C3'α), 70.4 (C6β) 70.4 (C6α), 69.3 (C4α), 69.0 (C5α), 68.6 (C4β), 68.5 (C5'β), 68.1 (C5'α), 67.8 (C7), 67.2 (C5β), 66.1(C6'β), 62.2 (C6'α), 60.4 (C4'α), 60.3 (C4'β), 60.1 (C2β), 56.4 (C2α), 36.5 (NCH_3β), 36.4 (NCH_3α), 32.7 (C3β), 27.3 (C3α); HRMS (ESI) *m/z* calculated for $C_{49}H_{45}N_9O_{13}$ $[M + Na]^+$ 990.3029; found 990.3024.

***N*-Phenyltrifluoroacetamidoyl 2,4'-di-azido-6,2',3',6'-tetra-*O*-benzoyl-2,4'-di-(desamino)-7'-*N*-phenyldiazenyl-aprabiosamine (21):** Hemiacetal **20** (86 mg, 0.088 mmol) was treated with 2,2,2-trifluoro-*N*-phenylacetimidoyl chloride (22 µL, 0.133 mmol) and Cs_2CO_3 (57 mg, 0.177 mmol) in acetone (1.5 mL) at room temperature. Upon completion of the reaction, the mixture was diluted with CH_2Cl_2 (20 mL), washed sequentially with saturated $NaHCO_3$ solution (20 mL) and brine (20 mL), dried over Na_2SO_4, and concentrated under reduced pressure. The residue was

purified by silica gel column chromatography to afford the titled compound **21** (96 mg, 88%) as a white foam; R_f = 0.35 in 35% EtOAc in hexane; (α:β=1:2.7); ^{1}H NMR (500 MHz, $CDCl_3$) δ 8.08 – 7.95 (m, 6H, Ar), 7.86 – 7.83 (m, 2H, Ar), 7.62 – 7.27 (m, 16H, Ar), 7.16 – 6.97 (m, 6H, Ar), 6.86 – 6.83 (m, 2H, Ar), (***α*-anomer**)- 6.01 (s, 1H, H6), 5.86 (t, *J* = 10.1 Hz, 1H, H3'), 5.77 (d, *J* = 8.0 Hz, 1H, H8), 5.73 (d, *J* = 3.7 Hz, 1H, H1'), 5.50 (br s, 1H, H1), 5.13 (dd, *J* = 10.3, 3.7 Hz, 1H, H2'), 5.09 (dd, *J* = 10.3, 3.7 Hz, 1H, H2'), 3.99 – 3.91 (m, 2H, H6'), 3.92 – 3.89 (m, 1H, H4), 3.82 (dd, *J* = 7.9, 2.0 Hz, 1H, H7), 3.76 (t, *J* = 10.2 Hz, 1H, H4'), 3.71 – 3.67 (m, 1H, H5'), 3.66 (d, *J* = 10.5, 3.4 Hz, 1H, H5), 3.53 – 3.51 (m, 1H, H2), 3. 31 (s, 3H, NCH_3), 2.08 (m, 1H, H3a), 1.93 (d, *J* = 11.8 Hz, 1H, H3b); (***β*-anomer**)- 6.00 (s, 1H, H6), 5.91 (d, *J* = 10.1 Hz, 1H, H3'), 5.77 (d, *J* = 8.0 Hz, 1H, H8), 5.75 (d, *J* = 3.9 Hz, 1H, H1'), 5.50 (d, *J* = d, *J* = 8.0 Hz, 1H, H1), 5.13 (dd, *J* = 10.3, 3.7 Hz, 1H, H2'), 3.99 – 3.87 (m, 3H, H6', H4), 3.82 – 3.81 (m, 1H, H7), 3.76 (t, *J* = 10.2 Hz, 1H, H4'), 3.70 (d, *J* = 10.9, 3.4 Hz, 1H, H5), 3.71 – 3.62 (m, 1H, H5'), 3.33 (s, 3H, NCH_3), 3.28 – 3.26 (m, 1H, H2) 1.30 (m, 1H, H3a), 0.87 (q, *J* = 12.1 Hz, 1H, H3b); ^{13}C NMR (126 MHz, $CDCl_3$) δ 166.0 (C=O), 165.8 (C=O), 165.7 (C=O), 165.1 (C=O), 150.0, 143.2, 133.7, 133.7, 133.5, 133.4, 130.2, 130.1, 130.0, 129.9, 129.9, 129.9, 129.8, 129.8, 129.5, 129.3, 129.2, 129.1, 128.9, 128.7, 128.6, 128.6, 128.5, 126.3, 124.7, 120.8, 120.6, 119.4, 97.6 (C1), 96.5 (C8), 94.2 (C1'), 74.9 (C6'), 71.5 (C2'), 70.4 (C3'), 69.4 (C5'), 67.9 (C6), 66.6 (C5), 66.2 (C7), 62.1 (C6'), 60.3 (C4'), 58.0 (C2), 36.6 (NCH_3), 32.8 (C3). HRMS (ESI) m/z calculated for $C_{57}H_{49}O_{13}N_{10}F_3$ $[M + Na]^+$ 1161.3324; found 1161.3354.

1,3,2′,4″-Tetra-azido-6,2″,3″,6″-tetra-*O*-benzoyl-1,3,2′,4″-tetra-(desamino)-6′,7′-oxazolidino-apramycin-5-one (26) and 1,3,2′,4″-Tetra-azido-6,2″,3″,6″-tetra-*O*-benzoyl-1,3,2′,4″-tetra-(desamino)-6′,7′-oxazolidino-apramycin-6-one (27):

Cs_2CO_3 (4.02 g,12.34 mmol) was added to a stirred solution of compound **24** (5.36 g, 4.94 mmol) in anhydrous DMF (98.0 mL) at room temperature. After 16 h, the reaction mixture was diluted with ethyl acetate (150 mL), washed with ice-cold water (150 mL), and the aqueous layer was

extracted with ethyl acetate (100 mL). The combined organic layers were washed with brine (100 mL), dried over Na_2SO_4, and concentrated to dryness, and the residue was purified by flash column chromatography to isolate a mixture of 6- and 5-*O*-benzoate derivatives **25** (5.28 g, 4.86 mmol). The mixture was dissolved in anhydrous CH_2Cl_2 (60 mL) and stirred with DMP (5.16 g, 12.152 mmol) at room temperature for 13 h. The reaction mixture was cooled to 0 °C and quenched with saturated aqueous $Na_2S_2O_3$ (60 mL). The organic layer was separated, washed sequentially with saturated $NaHCO_3$ (60 mL) and brine (60 mL), dried over Na_2SO_4, and concentrated under reduced pressure. The resulting crude mixture was purified by silica gel column chromatography, using ethyl acetate in hexane as the eluent (gradient: 10%, 20%, and 30%), to isolate compound **26** (2.10 g, 40%) and **27** (2.44 g, 46%) as white solids.

Compound 26: R_f = 0.33 in 30% EtOAc in hexane; $[\alpha]_D^{21}$ = (+)-112.1 (*c* = 1.0, $CHCl_3$); **^{1}H NMR** (500 MHz, $CDCl_3$): δ 8.08 – 8.00 (m, 6H, Ar), 7.96 – 7.93 (m, 2H, Ar), 7.64 – 7.58 (m, 2H, Ar), 7.57 – 7.44 (m, 6H, Ar), 7.44 – 7.39 (m, 2H, Ar), 7.38 – 7.34 (m, 2H, Ar), 5.99 (t, *J* = 10.1 Hz, 1H, H3''), 5.69 (d, *J* = 3.7 Hz, 1H, H1''), 5.39 (d, *J* = 10.8 Hz, 1H, H6), 5.15 (dd, *J* = 10.4, 3.7 Hz, 1H, H2''), 5.04 (d, *J* = 3.4 Hz, 1H, H1'), 4.75 (d, *J* = 6.4 Hz, 1H, H8'), 4.71 (dd, *J* = 6.4, 3.8 Hz, 1H, H6'), 4.68 – 4.59 (m, 2H, H6''), 4.46 (d, *J* = 10.5 Hz, 1H, H4), 4.09 (ddd, *J* = 10.6, 5.1, 2.5 Hz, 1H, H5''), 3.94 – 3.84 (m, 2H, H4'' & H1'), 3.79 – 3.71 (m, 2H, H5' & H7'), 3.55 (ddd, *J* = 12.4, 10.5, 4.6 Hz, 1H, H3), 3.45 (td, *J* = 10.8, 4.5 Hz, 1H, H4'), 2.98 (s, 3H, NCH_3), 2.96 (t, *J* = 4.0 Hz, 1H, H2'), 2.58 (dt, *J* = 13.9, 4.6 Hz, 1H, H2eq), 1.97 (q, *J* = 12.9 Hz, 1H, H2ax), 1.68 (dt, *J* = 11.8, 4.5 Hz, 1H, H3'eq), 1.25 (q, *J* = 11.8 Hz, 1H, H3'ax); **^{13}C NMR** (126 MHz, 500 MHz, $CDCl_3$): δ 194.9 (C=O), 166.2 (OC=O), 166.0 (OC=O), 165.7 (OC=O), 164.9 (OC=O), 157.0 (NC=O), 134.0, 133.8, 133.7, 130.2, 130.0, 129.9, 129.8, 129.4, 128.9, 128.9, 128.8, 128.7, 128.7, 128.6, 128.4, 101.9 (C8'), 96.7 (C1'), 96.1 (C1''), 80.3 (C4), 79.6 (C6), 72.2 (C6'), 71.2 (C2''), 70.4 (C3''), 70.1 (C5''), 67.2 (C7'), 66.0 (C4'), 63.4 (C6''), 61.0 (C1), 60.3 (C5'), 59.7 (C3), 58.3 (C4''), 55.3 (C2'),

32.4 (C2), 30.5 (NCH_3), 27.4 (C3'); HRMS (ESI) *m/z* calculated for $C_{50}H_{45}N_{13}O_{16}$ [M + Na]$^+$: 1106.2999 found 1106.2996.

Compound 27: R_f = 0.25 in 30% EtOAc in hexane; $[\alpha]_D^{21}$ = (+)-57.7 (*c* = 1.0, $CHCl_3$); **^{1}H NMR** (500 MHz, $CDCl_3$) δ 8.07 – 8.01 (m, 6H, Ar), 7.93 (m, 2H, Ar), 7.63 – 7.36 (m, 12H, Ar), 5.96 (t, *J* = 10.1 Hz, 1H, H3''), 5.69 (d, *J* = 3.7 Hz, 1H, H1''), 5.60 (d, *J* = 9.2 Hz, 1H, H5), 5.13 (dd, *J* = 10.4, 3.7 Hz, 1H, H2''), 5.08 (d, *J* = 3.5 Hz, 1H, H1'), 4.86 (d, *J* = 4.0 Hz, 1H, H8'), 4.76 (dd, *J* = 7.9, 3.4 Hz, 1H, H6'), 4.71 – 4.59 (m, 2H, H6''), 4.37 (dd, *J* = 10.4, 3.5 Hz, 1H, H5'), 4.27 (dd, *J* = 13.5, 6.0 Hz, 1H, H1), 4.11 – 4.05 (m, 1H, H5''), 4.03 – 3.94 (m, 2H, H4 & H3), 3.88 (t, *J* = 10.1 Hz, 1H, H4''), 3.79 (dd, *J* = 8.0, 4.0 Hz, 1H, H7'), 3.56 (td, *J* = 10.9, 4.4 Hz, 1H, H4'), 2.97 (dt, *J* = 12.2, 3.7 Hz, 1H, H2'), 2.82 (s, 3H, NCH_3), 2.66 (dt, *J* = 13.4, 4.9 Hz, 1H, H2eq), 1.80 (q, *J* = 12.8 Hz, 1H, H2ax), 1.71 – 1.65 (m, 1H, H3'eq), 1.38 (q, *J* = 12.0 Hz, 1H, H3'ax); **^{13}C NMR** (126 MHz, $CDCl_3$) δ 194.9 (C=O), 166.2 (OC=O), 166.2 (OC=O) 166.1 (OC=O), 165.1 (OC=O), 157.0 (NC=O), 134.1, 134.0, 134.0, 133.8, 130.1, 130.0, 129.8, 129.4, 128.9, 128.9, 128.9, 128.8, 128.6, 99.2 (C1'), 98.6 (C8'), 96.1 (C1''), 80.8 (C4), 78.5 (C5), 71.7 (C2''), 70.7 (C3''), 70.5 (C6'), 69.8 (C5''), 66.4 (C5'), 65.4 (C4'), 63.5 (C6''), 61.2 (C1), 61.0 (C4''), 60.4 (C7'), 58.4 (C3), 56.1 (C2'), 32.0 (C2), 30.1 (NCH_3), 29.0 (C3'); HRMS (ESI) *m/z* calculated for $C_{50}H_{45}N_{13}O_{16}$ [M + Na]$^+$: 1106.2994 found 1106.2992.

2,4′-Di-azido-2′,3′,6′-tri-*O*-benzoyl-2,4′-di-(desamino)-6′,7′-oxazolidino-α,β-aprabiosamine (28): To a stirred solution of ketone **27** (2.2 g, 1.21 mmol) in dry DMF (40 mL), Li_2CO_3 (0.27 g, 3.63 mmol) was added and stirring was continued at room temperature. After 6 h, the reaction mixture was neutralized by the dropwise addition of glacial acetic acid (0.45 mL, 8.12 mmol) and diluted with ethyl acetate (60 mL). The mixture was washed with deionized water (50 mL), and the aqueous layer was extracted twice with ethyl acetate (30 mL each). The combined organic layers were washed with brine (50 mL), dried over Na_2SO_4, and concentrated under vacuum. The crude product was purified by silica gel flash column chromatography, using ethyl acetate in hexane as the eluent (gradient: 20%, 30%, and 40%), to afford a 1:1 anomeric mixture of

aprabiosamine **28** (1.23 g, 79%) as a white solid and the side product **29** (0.161 g, 8%) also as a white solid, which was directly converted to **30** as described below.

Compound 28: R_f = 0.45 in 40% EtOAc in hexane; **^{1}H NMR** (500 MHz, $CDCl_3$) δ 8.08 – 7.95 (m, 12H, Ar), 7.65 – 7.36 (m, 18H, Ar), (***β*-anomer**)- 5.93 (t, *J* = 10.1 Hz, 1H, H3'), 5.73 (d, *J* = 3.8 Hz, 1H, H1'), 5.21 (dd, *J* = 10.1, 3.8 Hz, 1H, H2'), 5.09 (d, *J* = 2.2 Hz, 1H, H8), 4.91 (dd, *J* = 9.0, 3.3 Hz, 1H, H6), 4.57 (dd, *J* = 12.1, 2.4 Hz, 1H, H6a'), 4.60 (dd, *J* = 12.1, 5.6 Hz, 1H, H6b') 4.04 (ddd, *J* = 10.6, 5.7, 2.5 Hz, 1H, H5'), 3.92 (dd, *J* = 9.0, 2.2 Hz, 1H, H7), 3.87 (t, *J* = 10.1 Hz, 1H, H4'), 3.70 (dd, *J* = 10.3, 3.3 Hz, 1H, H5), 3.56 (m, 1H, H4), 3.36 (d, *J* = 7.3 Hz, 1H, H1), 3.09 – 3.02 (m, 1H, H2), 2.74 (s, 3H, NCH_3), 2.07 (dt, *J* = 12.4, 4.6 Hz, 1H, H3*eq*), 0.77 (q, *J* = 12.1 Hz, 1H, H3*ax*); (***α*-anomer**)- δ 5.95 (t, *J* = 10.1 Hz, 1H, H3'), 5.75 (d, *J* = 3.8 Hz, 1H, H1'), 5.25 (dd, *J* = 10.3, 3.8 Hz, 1H, H2'), 5.07 (d, *J* = 3.4 Hz, 1H, H1), 5.00 (d, *J* = 2.2 Hz, 1H, H8), 4.81 (dd, *J* = 8.2, 3.6 Hz, 1H, H6), 4.77 (dd, *J* = 12.1, 2.3 Hz, 1H, H6a'), 4.58 (dd, *J* = 12.2, 5.7 Hz, 1H, H6b'), 4.13 (dd, *J* = 10.5, 3.6 Hz, 1H, H5), 4.03 – 3.97 (m, 1H, H5'), 3.90 (t, *J* = 10.1 Hz, 1H, H4'), 3.90 (dd, *J* = 8.2, 3.7 Hz, 1H, H7), 3.63 (td, *J* = 10.9, 4.5 Hz, 1H, H4), 3.06 (ddt, *J* = 11.5, 4.4, 2.1 Hz, 1H, H2), 1.94 (dt, *J* = 11.5, 4.4 Hz, 1H, H3*eq*), 1.44 (q, *J* = 11.8 Hz, 1H, H3*ax*) **^{13}C NMR** (126 MHz, $CDCl_3$) δ δ 166.3 (C=O), 166.2 (C=O), 166.0 (C=O), 165.8 (C=O), 165.8 (C=O), 165.6 (C=O), 157.3 (NC=O), 157.2 (NC=O), 134.3, 134.0, 133.9, 133.8, 133.8, 130.3, 130.2, 130.0, 129.9, 129.8, 129.8, 129.4, 129.0, 128.8, 128.8, 128.8, 128.8, 128.7, 128.7, 128.6, 128.4, 98.3 (C1β), 94.6 (C8α), 93.3 (C8β), 91.9 (C1α), 91.8 (C1'α), 91.2 (C1'β), 71.2 (C2'β), 71.2 (C5β), 70.7 (C2'α), 70.7 (C5α), 70.4 (C3'β), 69.6 (C3'α), 69.4 (C6β), 68.8 (C5'β), 65.4 (C5'a), 65.2 (C6α), 64.4 (C4β), 63.7 (C6'β), 63.6 (C5'α), 61.2 (C6'α), 61.2 (C4'β), 59.8 (C4α), 59.8 (C4'α), (C7β), 59.2 (C2β), 56.4 (C7α), 53.6 (C2α), 34.5 (*N*CH_3β), 30.0 (NCH_3α), 29.7 (C3β), 28.2 (C3α) HRMS (ESI) *m/z* calculated for $C_{37}H_{35}N_7O_{13}$ $[M + Na]^+$: 808.2185, found 808.2234.

2,3-Dibenzoyloxyphenyl 2',4''-di-azido-6',2'',3'',6''-tetra-*O*-benzoyl-2',4''-di-(desamino)-6-7-oxazolidono-aprabiosaminide (30): Under an argon atmosphere, ketone **26** (82 mg, 0.071

mmol) in anhydrous DMF (2.0 mL) was stirred with Cs_2CO_3 (70 mg, 0.215 mmol). After 8 h, the reaction mixture was diluted with ethyl acetate (20 mL), washed with deionized water (20 mL), and the aqueous layer was extracted with ethyl acetate (20 mL). The combined organic layers were washed with brine (20 mL), dried over Na_2SO_4, concentrated to dryness, and purified by flash column chromatography using ethyl acetate in hexane (20–40%) to isolate a regioisomeric mixture of phenolic glycosides **29** (62 mg, 86%). This mixture was further treated with benzoyl chloride (15 µL, 0.093 mmol) and triethylamine (22 µL, 0.155 mmol) in CH_2Cl_2 (1.5 mL) at 0 °C, and the mixture was stirred for 2 h while gradually warming to room temperature. Upon completion, the reaction mixture was quenched with MeOH (100 µL), diluted with CH_2Cl_2 (15 mL), and washed sequentially with water (10 mL) and brine (10 mL). The organic layer was then dried over Na_2SO_4 and concentrated under reduced pressure. The resulting crude mixture was purified by silica gel column chromatography (eluent: 20–40% ethyl acetate in hexanes) to afford the per-*O*-benzoate **30** (62 mg, 91%) as a white solid.

R_f = 0.30 in 40% EtOAc in hexane; $[\alpha]_D^{21}$ = (+)-53.2 (*c* = 1, $CHCl_3$); ^{1}H NMR (500 MHz, $CDCl_3$) δ 8.08 – 8.04 (m, 4H, Ar), 8.03 – 7.99 (m, 4H, Ar), 7.88 – 7.83 (m, 2H, Ar), 7.63 – 7.56 (m, 2H, Ar), 7.55 – 7.48 (m, 4H, Ar), 7.47 – 7.36 (m, 4H, Ar), 7.36 – 7.32 (m, 2H, Ar), 7.29 – 7.25 (m, 1H, Ar), 7.19 – 7.07 (m, 6H, Ar), 5.97 (t, *J* = 10.1 Hz, 1H, H3'), 5.66 (d, *J* = 3.6 Hz, 1H, H1'), 5.39 (d, *J* = 3.2 Hz, 1H, H1), 5.16 (dd, *J* = 10.4, 3.6 Hz, 1H, H2'), 4.72 (d, *J* = 6.1 Hz, 1H, H8), 4.63 (dd, *J* = 12.2, 3.9 Hz, 2H, H6'), 4.51 (dd, *J* = 6.6, 3.7 Hz, 1H, H6), 4.04 (ddd, *J* = 10.7, 5.3, 2.5 Hz, 1H, H5'), 3.87 (t, *J* = 8.6, 1H, H4'), 3.84 (dd, *J* = 7.4, 2.9 Hz, 1H, H5), 3.49 – 3.41 (m, 2H, H4, H7), 3.08 (dt, *J* = 12.9, 3.9 Hz, 1H, H2), 2.93 (s, 3H, NCH_3), 1.67 – 1.59 (m, 1H, H3a), 1.24 (q, *J* = 12.1 Hz, 1H, H3b); ^{13}C NMR (126 MHz, $CDCl_3$) δ 166.2 (C=O), 166.1 (C=O), 165.7 (C=O), 164.3 (C=O), 163.9 (C=O), 157.0 (NC=O), 149.5, 144.2, 134.0, 134.0, 133.9, 133.8, 133.7, 130.4, 130.3, 129.9, 129.9, 129.8, 129.4, 128.9, 128.8, 128.7, 128.7, 128.7, 128.6, 128.6, 128.5, 126.6, 118.3, 100.5 (C8), 97.6 (C1), 95.3 (C1'), 72.0 (C6), 71.1 (C2'), 70.5 (C3'), 70.1 (C5'), 67.0 (C5), 65.8

(C4), 63.5 (C6'), 61.0 (C4'), 60.2 (H7), 55.7 (C2), 30.5 (CH_3), 27.5 (C3); HRMS (ESI) *m/z*: calculated for $C_{57}H_{47}N_7O_{17}$ [M + Na]$^+$: 1124.2920, found 1124.2924.

N*-Phenyltrifluoroacetamidoyl 2,4′-di-azido-2′,3′,6′-tri-*O*-benzoyl-2,4′-di(desamino)-6′,7′-oxazolidino-α,β-aprabiosamine (31)**: 2,2,2-Trifluoro-*N*-phenylacetimidoyl chloride (52 µL, 0.283 mmol) was added to a stirred solution of aprabiosamine hemiacetal **28** (150 mg, 0.193 mmol) and Cs_2CO_3 (93 mg, 0.283 mmol) in acetone (3.0 mL) at room temperature. Upon completion of the reaction, the mixture was diluted with CH_2Cl_2 (30 mL), washed sequentially with saturated aqueous $NaHCO_3$ solution (30 mL) and brine (30 mL), dried over Na_2SO_4, and concentrated under reduced pressure. The residue was purified by silica gel column chromatography to afford an anomeric mixture compound **31** (96 mg, 88%) as a white amorphous solid. R_f = 0.45 in 30% EtOAc in hexanes, ^{1}H NMR (600 MHz, $CDCl_3$) δ 8.10 – 7.94 (m, 7H, Ar), 7.67 – 7.34 (m, 13H, Ar), 7.33 – 7.28 (m, 2H, Ar), 6.85 (d, *J* = 7.8 Hz, 2H, Ar), (α*-anomer**)- 6.00 (t, *J* = 10.1 Hz, 1H, H3'), 5.69 (d, *J* = 3.7 Hz, 1H, H1'), 5.18 (dd, *J* = 10.5, 3.7 Hz, 1H, H2'), 5.01 (br s, 1H, H1), 4.99 – 4.97 (m, 1H, H6) 4.76 (d, *J* = 2.2 Hz, H8), 4.68 – 4.63 (m, 2H, H6') 4.12 – 4.09 (m, 1H, H5'), 3.86 (t, *J* = 10.1 Hz, 1H, H4'), 3.83 – 3.81 (m, 1H, H5), 3.80 – 3.74 (m, 1H, H4), 3.55 (d, *J* = 8.2 Hz, H5), 3.53 – 3.51 (m, 1H, H7), 3.49 (td, *J* = 10.7, 4.4 Hz, H4), 3.25 (dt, *J* = 12.0, 6.8 Hz, 1H, H2), 2.97 (s, 3H, NCH_3), 1.81 (dt, *J* = 12.0, 4.5 Hz, 1H, H3a), 0.98 (q, *J* = 12.0 Hz, 1H, H3b); (***β*-anomer**)- 5.93 (t, *J* = 10.0 Hz, 1H, H3'), 5.74 (d, *J* = 3.8 Hz, 1H, H1'), 5.22 (dd, *J* = 10.3, 3.9 Hz, 1H, H2'), 5.06 (d, *J* = 2.3 Hz, 1H, H8), 4.96 (d, *J* = 8.7, 2,0 Hz, 1H, H6) 4.74 (dd, *J* = 12.2, 2.4 Hz, 1H, H6'a), 4.70 (d, *J* = 7.8 Hz, 1H, H1), 4.57 (dd, *J* = 12.1, 5.8 Hz, 1H, H6'b), 3.97 (ddd, *J* = 10.7, 5.8, 2.4 Hz, 1H, H5'), 3.88 (dd, *J* = 9.5, 3.5 Hz, H5), 3.85 (t, *J* = 10.1 Hz, 1H, H4'), 3.86 (dd, *J* = 10.1 Hz, 1H, 3.76 – 3.63 (m, 2H, H4, H7), 3.42 (dt, *J* = 13.0, 6.8 Hz, 1H, H2), 2.75 (s, 3H, NCH_3), 2.17 (dt, *J* = 12.8, 3.9 Hz, 1H, H3a), 0.85 (q, *J* = 12.8 Hz 1H, H3b); ^{13}C NMR (151 MHz, $CDCl_3$) δ 166.2 (C=O), 166.1(C=O), 165.9(C=O), 165.7(C=O), 165.7(C=O), 165.6(C=O), 156.9 (NC=O), 156.8 (C=O), 143.2, 143.0, 134.0, 134.0, 133.9, 133.8, 133.7, 130.0, 129.9, 129.9, 129.8, 129.8,

129.7, 129.0, 128.9, 128.8, 128.8, 128.8, 128.7, 128.6, 128.6, 128.6, 128.1, 124.6, 119.3, 115.7 (q, J = 286 Hz, -CF_3), 101.8 (C1β), 97.0 (C8β), 95.5 (C8α), 92.3 (C1α), 91.4 (C1'β), 91.4 (C1'α), 71.9 (C2'β), 71.5 (C2'α), 71.1 (C4α), 71.1(C4β), 70.5 (C3'α), 70.3 (C5'β), 70.2 (C3'β), 69.4 (C5'α), 68.5 (C6β), 68.4 (C6α), 65.2 (C7β), 63.8 (C6'β), 63.6 (C7α), 63.4 (C6'α), 61.1 (C4β), 60.8 (C5α), 60.4 (C4α), 60.3 (C5β), 59.2 (C2α), 57.6 (C2β), 34.2 (C3β), 30.4 (NCH_3α), 29.7 (NCH_3β). 27.8 (C3α); HRMS (ESI) *m/z*: calculated for $C_{45}H_{39}N_8O_{13}F_3$ $[M + Na]^+$: 979.2480, found 979.2490.

1,3,2′,4″-Tetra-azido-2,5,6-tri-*O*-benzyl-1,3,2′,4″-tetra-(desamino)-2-hydroxy-6′,7′-oxazolidino-1'-epi-apramycin (33) and 1,3,2′,4″-Tetra-azido-2,5,6-tri-*O*-benzyl-1,3,2′,4″-tetra-(desamino)-2-hydroxy-6′,7′-oxazolidino-apramycin (34): Glycosyl donor **31** (161 mg, 0.168 mmol) and acceptor **22** (85 mg, 0.168 mmol), along with freshly dried 4 Å molecular sieves (200 mg), were stirred in anhydrous CH_2Cl_2 (1.6 mL) for 30 min. To this mixture, TfOH (8 µL, 0.088 mmol) was added, and stirring was continued for 1 h at room temperature. After completion, the reaction mixture was quenched with triethylamine (50 µL), diluted with CH_2Cl_2 (20 mL), filtered through a bed of Celite®, and concentrated under reduced pressure. The crude mixture was then subjected to silica gel column chromatography, eluting with a gradient of ethyl acetate in hexanes (10→25%), yielding an anomeric mixture of glycosides **32** (95 mg, 68%) as a white solid. The solid was dissolved in a 1:1 mixture of CH_2Cl_2 and MeOH (2.0 mL), and sodium methoxide (22 mg, 0.380 mmol) was added. The mixture was stirred for 4 h at room temperature then was neutralized with IRC-120 H^+ resin, filtered through a cotton plug, and concentrated under vacuum. The crude products were purified by silica gel column chromatography, eluting with a gradient of ethyl acetate in hexanes (20→35%), to afford compounds **33** (45 mg, 62%) and **34** (17 mg, 22%) both as white amorphous solids. **Compound 33:** R_f = 0.45 in 40% EtOAc in hexane; $[\alpha]_D^{21}$ = (+)-112.5 (c = 1.0, $CHCl_3$); ^1H NMR (600 MHz, $CDCl_3$) δ 7.47 – 7.28 (m, 15H, Ar), 5.32 (d, J = 3.8 Hz, 1H, H1''), 5.02 (d, J = 10.4 Hz, 1H, CH_2Ph), 4.96 (d, J = 3.2 Hz, 1H, H8'), 4.92 (d, J = 7.9 Hz, 1H, H1'), 4.90 (d, J = 10.3 Hz, 1H, CH_2Ph), 4.84 – 4.79 (m, 2H, CH_2Ph), 4.76 (dd, J = 8.4, 3.3 Hz,

1H, H6’), 4.74 (d, *J* = 10.5 Hz, 1H, CH_2Ph), 4.64 (d, *J* = 10.4 Hz, 1H, CH_2Ph), 4.03 (dd, *J* = 10.1, 3.3 Hz, 1H, H5’), 3.87 – 3.75 (m, 4H, H6’’, H3’’, H4’), 3.71 (dd, *J* = 8.4, 3.2 Hz, 1H, H7’), 3.65 (dd, *J* = 9.3, 4.4 Hz, 1H, H2’’), 3.60 (t, *J* = 9.7 Hz, 1H, H5), 3.56 (t, *J* = 9.7 Hz, 1H, H6), 3.56 (t, *J* = 9.7 Hz, 1H, H4), 3.53 – 3.48 (m, 2H, H1, H4’’), 3.47 – 3.44 (m, 1H, H5’’), 3.33 (ddd, *J* = 12.2, 7.8, 4.8 Hz, 1H, H2’), 3.28 (t, *J* = 9.7 Hz, 1H, H2), 3.14 (t, *J* = 9.8 Hz, 1H, H3), 2.93 (s, 3H, NCH_3), 2.91 (s, 1H, OH) 2.73 (s, 1H, OH), 2.35 (dt, *J* = 12.2, 4.8 Hz, 1H, H3’eq), 1.97 (s, 1H, OH), 1.70 (q, *J* = 12.2,Hz, 1H, H3’ax). **^{13}C NMR** (151 MHz, $CDCl_3$) δ 157.3 (NC=O), 137.9, 137.6, 136.9, 128.9, 128.6, 128.6, 128.6, 128.5, 128.3, 128.1, 128.0, 104.5 (C1’), 94.6 (C1’’), 93.0 (C8’), 81.9 (C5’’), 80.4 (C2’), 79.6 (C2), 78.5 (C5’), 76.5 (CH_2Ph), 76.0 (CH_2Ph), 75.9 (CH_2Ph), 73.0 (C3’’), 71.9 (C6) 71.4 (C7’), 71.2 (C5’), 69.7 (C6’), 67.4 (C1), 67.2 (C3), 65.1 (C6’’), 61.7 (C3’’), 61.4 (C4’), 59.5 (C4’’), 59.4 (C2’) 34.2 (C3’), 30.0 (NCH_3); HRMS (ESI) *m/z* calculated for $C_{43}H_{49}N_{13}O_{13}$ [M + Na]$^+$: 978.3465, found 978.3471.

Compound 34: R_f = 0.35 in 40% EtOAc in hexane; $[\alpha]_D^{21}$ = (+)-98.5 (*c* = 1.0, $CHCl_3$); ^{1}H NMR (600 MHz, Chloroform-*d*) δ 7.49 – 7.26 (m, 15H, Ar), 5.49 (d, *J* = 3.7 Hz, 1H, H1’), 5.34 (d, *J* = 3.8 Hz, 1H, H1’’), 5.00 (d, *J* = 10.6 Hz, 1H, CH_2Ph), 4.97 (d, *J* = 3.4 Hz, 1H, H8’), 4.95 (d, *J* = 10.4 Hz, 1H, CH_2Ph), 4.88 (d, *J* = 10.5 Hz, 1H, CH_2Ph), 4.86 (d, *J* = 10.5 Hz, 1H, CH_2Ph), 4.82 (dd, *J* = 8.5, 3.1 Hz, 1H, H6’), 4.82 – 4.76 (m, 2H, CH_2Ph), 4.67 (dd, *J* = 10.5, 3.4 Hz, 1H, H5’), 3.84 (t, *J* = 9.3 Hz, 1H, H3’’), 3.82 – 3.78 (m, 2H, H6’’), 3.81 – 3.78 (m, 2H, H4’, H7’), 3.65 (t, *J* = 9.1 Hz, 1H, H6), 3.67 – 3.58 (m, 2H, H2’’, H3), 3.55 (t, *J* = 10.0 Hz, 1H, H5), 3.51 – 3.44 (m, 2H, H4’’, H5’’)., 3.45 (t, *J* = 9.7 Hz, 1H, H1), 3.39 (t, *J* = 9.4 Hz, 1H, H2), 3.34 (t, *J* = 9.9 Hz, 1H, H4), 3.16 (dt, *J* = 12.7, 4.2 Hz, 1H, H2’), 3.03 (s, 1H, OH), 2.92 (s, 3H, NCH_3), 2.70 (s, 1H, OH), 2.28 (dt, *J* = 9.0, 4.4 Hz, 1H, H3’eq), 2.21 (q, *J* = 11.6 Hz, 1H, H3’ax), 1.94 (s, 1H, OH); ^{13}C NMR (151 MHz, $CDCl_3$) δ 156.1 (NC=O), 136.7, 136.1, 135.6, 127.7, 127.5, 127.5, 127.4, 127.1, 127.1, 126.7, 126.1, 97.2 (C1’), 94.2 (C1’’), 92.4 (C8’), 82.3 (C2’’), 80.2 (C2), 78.1 (C4), 76.2 (C5), 76.0 (CH_2Ph), 75.8 (CH_2Ph), 75.0 (CH_2Ph), 74.8 (C3’’), 74.4 (C5’’), 74.0 (C6), 72.1 (C6’), 70.8 (C3), 70.6 (C5’),

69.1 (C7’), 66.2 (C4’’), 64.6 (C6’’), 64.1 (C4’), 60.9 (C6’), 60.1 (C1), 58.9 (C3), 55.0 (C2’), 28.9 (C3’), 28.5 (NCH_3); HRMS (ESI) *m/z* calculated for $C_{43}H_{49}N_{13}O_{13}$ [M + Na]$^+$: 978.3465, found 978.3473.

1’-epi-2-Hydroxy-apramycin pentaacetate salt (35):

A stirred solution of compound **33** (51 mg, 0.053 mmol) in 1,4-dioxane (0.8 mL) was treated with 3N NaOH (0.4 mL) and heated at 90 °C for 6 h. The reaction mixture was cooled to room temperature and neutralized with glacial acetic acid and concentrated *in vacuo*. The residue was passed through a silica gel column (eluent: 50% methanol/dicholoromethane) and the product-containing fractions were concentrated, dissolved in dioxane:water:glacial acetic acid = 1:2:0.2 (0.8 mL). Pd/OH_2 (55 mg, 20%) was added and the reaction mixture was stirred at room temperature under 1 atm of hydrogen (balloon) for 22 h. After completion, the reaction mixture was filtered using a syringe fitted with a 20 μm pore size filter, concentrated to dryness and dissolved in aqueous acetic acid solution (pH 4, 1 mL) before it was charged to a Sephadex column (CM Sephadex C-25). The column was flushed with deionized water (20 mL), then eluted with a gradient of 0.1% - 1.0% ammonium hydroxide in deionized water. The fractions containing the product were combined, acidified with glacial acetic acid and lyophilized to afford **35** (18 mg, 39%) as peracetate salt in the form of a white solid; $[\alpha]_D^{21}$ = (+)-96.6 (*c* = 1.0, D_2O), ^{1}H NMR (500 MHz, D_2O) δ 5.39 (d, *J* = 4.0 Hz, 1H, H1’’), 5.12 (d, *J* = 8.4 Hz, 1H, H1’), 4.97 (d, *J* = 8.5 Hz, 1H, H8’), 4.52 (s, 1H, H6’), 3.89 – 3.83 (m, 2H, H4’, H6), 3.82 – 3.78 (m, 1H, H5’’), 3.77 – 3.73 (m, 2H, H2, H6’’b), 3.69 (dd, *J* = 12.5, 4.8 Hz, 1H, H6’’a), 3.61 (m, 2H, H2’’, H5’), 3.56 (t, *J* = 9.7 Hz, 1H, H5), 3.48 (t, *J* = 8.8 Hz, 1H, H3’’), 3.45 (t, *J* = 9.8 Hz, 1H, H4), 3.30 – 3.22 (m, 2H, H2’, H7’), 3.15 (t, *J* = 10.4 Hz, 1H, H1), 3.13 – 3.08 (m, 1H, H5’’), 3.05 (t, *J* = 10.5 Hz, 1H, H4’’), 2.68 (s, 3H, NCH_3), 2.49 – 2.43 (m, 1H, H3’a), 1.75 (q, *J* = 12.1 Hz 1H, H3’b); ^{13}C NMR (151 MHz, D_2O) δ 181.1 (C=O), 100.0 (C8’), 94.5 (C1’’), 93.0 (C1’), 77.0 (C4’), 75.7 (C3’’), 72.2 (C2’’), 70.3 (C5’), 69.9 (C4), 69.8 (C6), 68.8 (C5’’), 67.9 (C5), 65.9 (C2), 62.8 (C6’), 60.4 (C6’’), 59.3 (C2’), 56.2

(C4''), 56.0 (C3), 52.1 (C1), 49.3 (C7'), 30.8 (C3'), 30.1 (NCH_3), 23.0 ($COCH_3$); HRMS (ESI) *m/z* calculated for $C_{21}H_{42}N_5O_{12}$ [M + H]$^+$: 556.2824, found 556.2813.

2-Hydroxy-apramycin pentaacetate salt (2): A stirred solution of compound **34** (32 mg, 0.033 mmol) in 1,4-dioxane (0.6 mL) was treated with 3N NaOH (0.3 mL) and heated at 90 °C for 8 h. The reaction mixture was cooled to room temperature and neutralized with glacial acetic acid and concentrated *in vacuo*. The crude product was passed through a silica gel column (eluent: 50% methanol/dichloromethane). The product-containing fractions were concentrated, dissolved in dioxane:water:glacial acetic acid = 1:2:0.2 (0.6 mL). Pd/OH_2 (31 mg, 20%) was added and the reaction mixture was stirred at room temperature under 1 atm of hydrogen (balloon) for 23 h. After completion, the reaction mixture was filtered using a syringe fitted with a 20 μm pore size filter, concentrated to dryness and dissolved in aqueous acetic acid solution (pH 4, 1 mL) before it was charged to a Sephadex column (CM Sephadex C-25). The column was flushed with deionized water (20 mL), then eluted with a gradient of 0.1% - 1.0% ammonium hydroxide in deionized water. The fractions containing the product were combined, acidified with glacial acetic acid and lyophilized to afford **2** (6 mg, 21%) as peracetate salt in the form of a white solid. $[\alpha]_D^{21}$ = (+)-88.6 (*c* = 0.4, D_2O), ^{1}H NMR (500 MHz, D_2O) δ 5.69 (d, *J* = 3.9 Hz, 1H, H1'), 5.55 (d, *J* = 4.0 Hz, 1H, H1''), 5.26 (d, *J* = 8.6 Hz, 1H, H8'), 4.59 (s, 1H, H6'), 4.02 – 3.98 (m, 2H, H4', H5), 3.96 (d, *J* = 10.1 Hz, 1H, H2), 3.90 – 3.84 (m, 2H, H6''), 3.83 – 3.80 (m, 1H, H5''), 3.79 – 3.72 (m, 3H, H2'', H5'', H6), 3.72 – 3.65 (m, 3H, H2', H5', H4), 3.59 (t, *J* = 9.5 Hz, 1H, H3''), 3.43 (dd, *J* = 8.5, 2.9 Hz, 1H, H7'), 3.30 (t, *J* = 10.3 Hz, 1H, H3), 3.23 (t, *J* = 9.7 Hz, 1H, H4''), 3.19 (t, *J* = 10.5 Hz, 1H, H1), 2.84 (s, 3H, NCH_3), 2.46 – 2.37 (m, 1H, H3'a), 2.08 (q, *J* = 12.1 Hz, 2H, H3'b); ^{13}C NMR (151 MHz, D_2O) δ 180.61 (C=O), 95.6 (C1'), 94.5 (C1''), 93.0 (C8'), 77.7 (C2''), 75.2 (C5''), 70.3 (C6), 69.8 (C3''), 69.8 (C5'), 69.6 (C5), 68.6 (C2), 67.8 (C4), 66.1 (C4'), 62.8 (C6'), 60.4 (C6''), 59.5 (C7'), 56.3 (C1), 54.9 (C4''), 52.1 (C3), 48.1 (C2'), 30.1 (NCH_3), 27.0 (C3'), 22.8 (CH_3); HRMS (ESI) *m/z* calculated for $C_{21}H_{42}N_5O_{12}$ [M + H]$^+$: 556.2824, found 556.2813.

Methyl 2,4′-di-azido-2′,3′,6′-tri-*O*-benzoyl-2,4′-di-(desamino)-6′,7′-oxazolidino-α,β-aprabiosaminide (36): A stirred solution of glycosyl donor **31** (73 mg, 0.076 mmol), acceptor methanol (9 µL, 0.096 mmol), and freshly activated 4 Å molecular sieves (200 mg) was stirred for 10 minutes at room temperature. TfOH (3.0 µL, 0.019 mmol) was added and stirred for an additional hour. After the reaction was complete, the solution was neutralized with triethylamine (20 µL, 0.051 mmol), filtered through a pad of Celite®, and concentrated under reduced pressure. The crude product was purified by silica gel column chromatography, using a gradient of ethyl acetate in hexanes (20→40%) to isolate methyl glycoside **36** (46 mg, 77%) as an anomeric mixture (α:β = 1:1.4) in the form of a white solid; R_f 0.33 in 30% EtOAc in hexanes, ^{1}H NMR (600 MHz, $CDCl_3$) δ 8.08 – 7.93 (m, 10H, Ar), 7.67 – 7.35 (m, 20H, Ar), (***α*-anomer**)- 5.95 (t, *J* = 10.0 Hz, 1H, H3’), 5.76 (d, *J* = 3.4 Hz, 1H, H1’), 5.14 (dd, *J* = 10.4, 3.7 Hz, 1H, H2’), 4.92 (d, *J* = 2.0 Hz, 1H, H8), 4.77 (dd, *J* = 7.8, 3.6 Hz, 1H, H6), 4.75 – 4.60 (m, 2H, H6’), 4.58 (d, *J* = 3.9 Hz, 1H, H1), 4.01 (ddd, *J* = 10.7, 5.7, 2.3 Hz, 1H, H5’), 3.91 (dd, *J* = 7.7, 1.9 Hz, H7), 3.87 (t, *J* = 10.2 Hz, 1H, H4’), 3.75 (dd, *J* = 10.3, 3.7 Hz, H5), 3.55 (td, *J* = 10.8, 4.4 Hz, 1H, H4), 3.09 (s, 3H, OCH_3), 2.99 (dt, *J* = 12.9, 3.9 Hz, 1H, H2), 2.86 (s, 3H, NCH_3), 1.86 (dt, *J* = 11.4, 4.5 Hz, 1H, H3a), 1.36 (q, *J* = 11.8 Hz, H3b), (***β*-anomer**)- 5.93 (t, *J* = 10.0 Hz, 1H, H3’), 5.77 (d, *J* = 3.4 Hz, 1H, H1’), 5.20 (dd, *J* = 10.3, 3.9 Hz, 1H, H2’), 5.09 (s, 1H, H8), 4.91 (d, *J* = 9.0 Hz, H6), 4.78 – 4.58 (m, 2H, H6’), 3.96 (ddd, *J* = 8.3, 5.7, 2.0 Hz, 1H, H5’), 3.91 (d, *J* = 9.1 Hz, H7), 3.87 (t, *J* = 10.2 Hz, 1H, H4’), 3.68 – 3.61 (m, 2H, H4 & H5), 3.19 (s, 3H, OCH_3), 3.10 (ddd, *J* = 12.6, 7.7, 4.8 Hz, H2), 2.95 (d, *J* = 7.8 Hz, 1H, H1), 2.71 (s, 3H, NCH_3), 2.08 (dt, *J* = 11.2, 3.9 Hz, 1H, H3a), 1.36 (q, *J* = 11.8 Hz, 1H, H3b); ^{13}C NMR (150 MHz, $CDCl_3$) δ 166.2 (C=O), 166.2 (C=O), 166.1 (C=O), 165.9 (C=O), 165.8 (C=O), 165.6 (C=O), 157.1 (NC=O), 157.1 (NC=O), 134.2, 134.0, 133.9, 133.8, 133.8, 133.8, 130.4, 130.2, 129.9, 129.9, 129.8, 129.8, 129.4, 129.0, 128.9, 128.8, 128.8, 128.7, 128.7, 128.6, 128.5, 104.8 (C1β), 98.9 (C1α), 96.3 (C8α), 93.9 (C1’α), 91.4 (C1’β), 90.3 (C8β), 71.5 (C2’α), 71.4 (C2’β), 70.8 (C3’α), 70.7 (C3’β), 70.7 (C6α), 70.6 (C4β), 69.7 (C5’β), 69.3 (C5’α),

68.5 (C6β), 65.6 (C4α), 65.4 (C5α), 64.3 (C4β), 63.6 (C6'α), 63.6 (C6'β), 61.2 (C4'α), 61.1 (C4'β), 60.0 (C5β), 59.1 (C7α), 58.2 (C2β), 56.7 (OCH3α), 55.9 (OCH3β), 55.4 (C2α), 34.7 (C3β), 30.1 (NCH3α), 29.6 (NCH3β), 28.6 (C3α); HRMS (ESI) *m/z* calculated for $C_{38}H_{37}N_7O_{13}$ [M + Na]$^+$: 822.2341, found 822.2326.

Methyl α,β-aprabiosaminide triacetate salt (37): A stirred solution of compound **36** (60 mg, 0.02 mmol) in dioxane (1.0 mL) was treated with 3N NaOH (0.5 mL) and heated at 90 °C for 8 h. The reaction mixture was cooled room temperature and 1M $P(CH_3)_3$ in THF (0.45 mL) was added and stirring continued for 6 h at 60 °C. The reaction mixture was neutralized with glacial acetic acid, concentrated *in vacuo*, and purified through a CM Sephadex C-25 column, loading in 10% aqueous acetic acid and eluting with 0.1% - 1.0% ammonium hydroxide in deionized water. The fractions containing the product were combined and lyophilized with glacial acetic acid to afford compound **37** (25 mg, 74%) as triacetate salt in the form of white solid. ^{1}H NMR (500 MHz, D_2O) (***α*-anomer**)- δ 5.51 (d, *J* = 3.7 Hz, 1H, H1'), 5.24 (d, *J* = 8.4 Hz, 1H, H8), 4.96 (d, *J* = 3.5 Hz, 1H, H1), 4.57 (t, *J* = 2.8 Hz, 1H, H6), 4.02 – 3.98 (m, 1H, H5'), 3.96 (t, *J* = 7.8 Hz, 1H, H3'), 3.90 (ddd, *J* = 11.7, 10.2, 4.5 Hz, 1H, H4), 3.88 – 3.77 (m, 2H, H6'), 3.56 (ddd, *J* = 12.6, 4.7, 3.6 Hz, 1H, H2), 3.68 (dd, *J* = 10.1, 2.7 Hz, 1H, H5), 3.30 (t, *J* = 10.3 Hz, 2H, H4'), 3.31 (dd, *J* = 8.5, 2.8 Hz, 1H, H7), 2.81 (s, 3H, NCH_3), 2.35 (dt, *J* = 10.1, 4.7 Hz, 1H, H3a), 1.98 (q, *J* = 12.0 Hz, 1H, H3b); (***β*-anomer**)- 5.50 (d, *J* = 3.7 Hz, 1H, H1'), 5.22 (d, *J* = 8.4 Hz, 1H, H8), 4.71 (d, *J* = 8.4 Hz, 1H, H1), 4.62 (t, *J* = 2.8 Hz, 1H, H6), 4.02 – 3.98 (m, 1H, H5'), 3.92 (td, *J* = 10.7, 4.6 Hz, 1H, H4), 3.88 – 3.79 (m, 2H, H6'), 3.76 (t, *J* = 7.8 Hz, 1H, H3'), 3.52 (dd, *J* = 10.0, 2.7 Hz, 1H, H5), 3.46 (s, 3H, OCH_3β), 3.37 (dd, *J* = 8.6, 2.8 Hz, 1H, H7), 3.30 (t, *J* = 10.3 Hz, H, H4'), 3.22 (ddd, *J* = 12.8, 8.4, 4.6 Hz, 1H, H2), 2.81 (s, 3H, NCH_3), 2.55 (dt, *J* = 11.9, 4.7 Hz, 1H, H3a), 1.85 (q, *J* = 12.1 Hz, 1H, H3b); ^{13}C NMR (126 MHz, D_2O) δ 180.5 (C=O), 102.2 (C1b), 95.7 (C1a), 94.6 (C1'a), 94.5 (C1'b), 93.0 (C8a), 93.0 (C3b), 75.0 (C5b), 70.4 (C5a), 69.6 (C3'a), 68.5 (C2'a), 68.5, 66.3 (C4a), 66.2 (C4b), 62.9 (C6a), 62.8 (C6b), 60.4 (C6'a), 60.4 (C6'b), 59.7 (C7a), 59.4 (C7b), 57.3

(OCH_3a), 55.2 (OCH_3b), 52.2 (C4'a), 52.1 (C4'b) 49.6 (C2a), 47.9 (C2b), 30.5 (C3b), 30.1 (NCH_3a), 30.1 (NCH_3b), 27.4 (C3a), 22.7 (CH_3); HRMS (ESI) *m/z* calculated for $C_{16}H_{32}N_3O_9$ [M + H]$^+$: 410.2133, found 410.2130.

Cell-Free Translation Assays.

Cell-free in-vitro translation inhibition assays were performed using luciferase mRNA and bacterial S30 extracts containing either wild-type bacterial or human hybrid ribosomes. In brief, firefly luciferase mRNA was transcribed in vitro using T7 RNA polymerase (Thermo) using a plasmid as template in which the mammalian promoter in pGL4.14 (Promega) has been replaced by theT7 bacteriophage promoter. Test articles in aqueous solution containing 0.3% Tween20 were dispensed into white 96-well plates (Eppendorf) using the TECAN D300e digital dispenser. The test article dispension volume was balanced to a total of 1.5 µL by 0.3% Tween20 in water. The reaction volume was brought to 15 µL by addition of 13.5 µL Translation Master Mix comprised of bacterial S30 extract, 0.2 mM amino acid mix, 6 µg tRNA (Sigma), 0.4 µg hFluc mRNA, 0.3 µL protease inhibitor (cOmplete, EDTA-free, Roche), 12 U RNAse inhibitor (Ribolock, Thermo Scientific), and 6 µL S30 premix without amino acids (Promega). Dispension and mixing of reagents was performed on ice prior to incubating the sealed plates at 37 °C. After 1 h of incubation, the reaction was stopped on ice and 75 µL of luciferase assay reagent (Promega) was added to each well. Luminescence was recorded with a plate reader (BIO-TEK FLx800, Witec AG, Littau, Switzerland).

Antibacterial Assays.

The minimal inhibitory concentrations (MIC) of synthesized compounds were determined by broth microdilution assays according to CLSI reference methodology M07[1] as described previously[2] and using strains described previously.[3] Clinical bacterial isolates were obtained from the diagnostic laboratories of the Institute of Medical Microbiology, University of Zurich.

References.

1. Clinical Laboratory Standards Institute (2015). Methods for Dilution Antimicrobial Susceptibility Tests for Bacteria That Grow Aerobically-Tenth Edition: Approved Standard M07-A10. CLSI, Wayne, PA, USA.
2. Juhas, M.; Widlake, E.; Teo, J.; Huseby, D. L.; Tyrrell, J. M.; Polikanov, Y.; Ercan, O.; Petersson, A.; Cao, S.; Aboklaish, A. F.; Rominski, A.; Crich, D.; Böttger, E. C.; Walsh, T. R.; Hughes, D. E.; Hobbie, S. N., In-vitro Activity of Apramycin Against Multidrug-, Carbapenem-, and Aminoglycoside-Resistant Enterobacteriaceae and Acinetobacter baumannii. *J. Antimicrob. Chemother.* **2019,** *74*, 944-952.
3. Lubriks, D.; Zogota, R.; Sarpe, V. A.; Matsushita, T.; Sati, G. C.; Haldimann, K.; Gysin, M.; Böttger, E. C.; Vasella, A.; Suna, E.; Hobbie, S. N.; Crich, D., Synthesis and Antibacterial Activity of Propylamycin Derivatives Functionalized at the 5''- and Other Positions with a View to Overcoming Resistance due to Aminoglycoside Modifying Enzymes. *ACS Infect. Dis.* **2021,** *7*, 2413-2424.

^{1}H NMR Spectrum (500 MHz, $CDCl_3$) of 1,3,2',4''-Tetra-azido-6,6',2'',3'',6''-penta-*O*-benzoyl-1,3,2',4''-tetra-(desamino)-7'-*N*-phenyldiazenyl-apramycin (**17**)

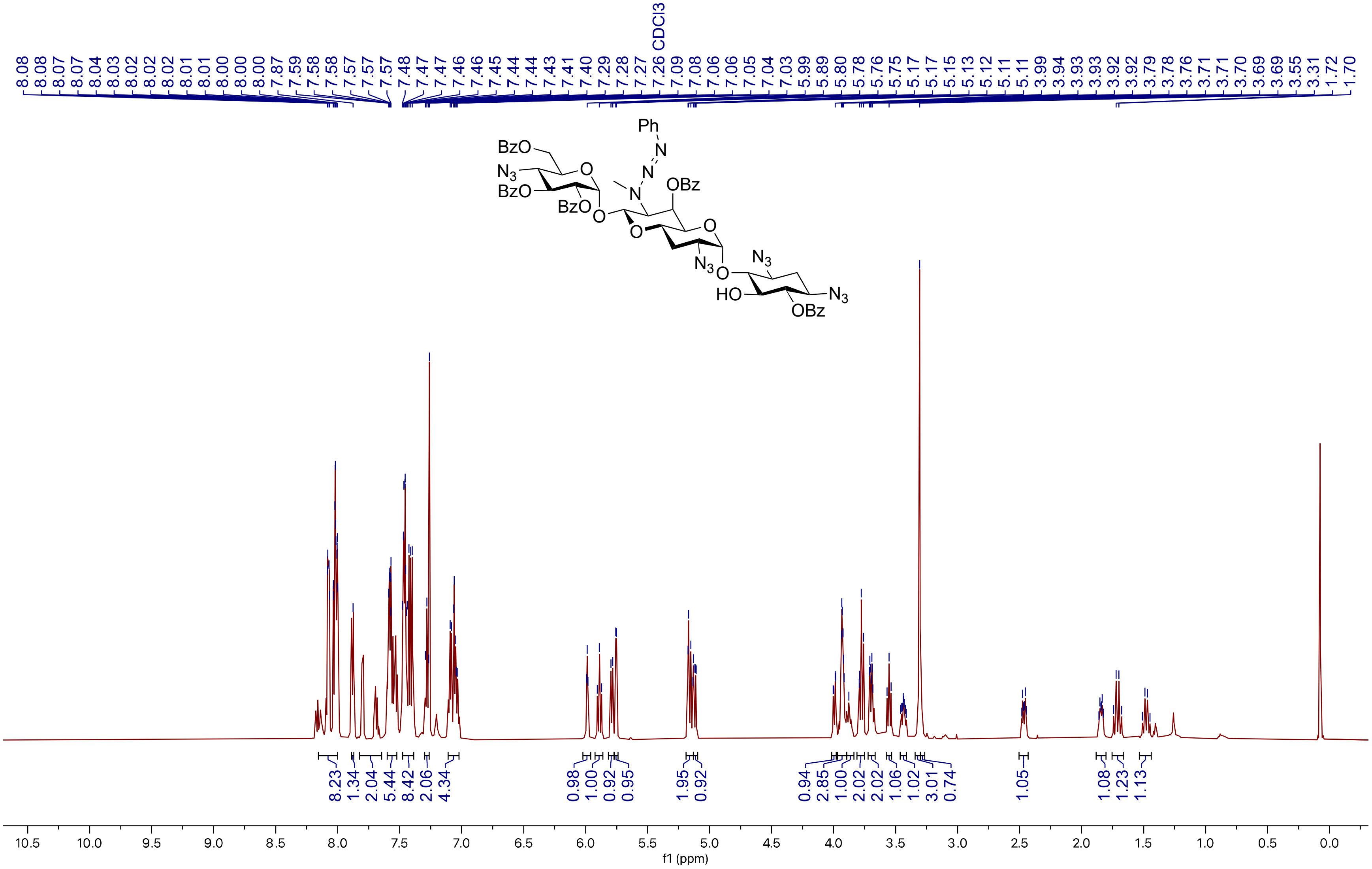

^{13}C NMR Spectrum (125 MHz, $CDCl_3$) of 1,3,2',4''-Tetra-azido-6,6',2'',3'',6''-penta-*O*-benzoyl-1,3,2',4''-tetra-(desamino)-7'-*N*-phenyldiazenyl-apramycin (**17**)

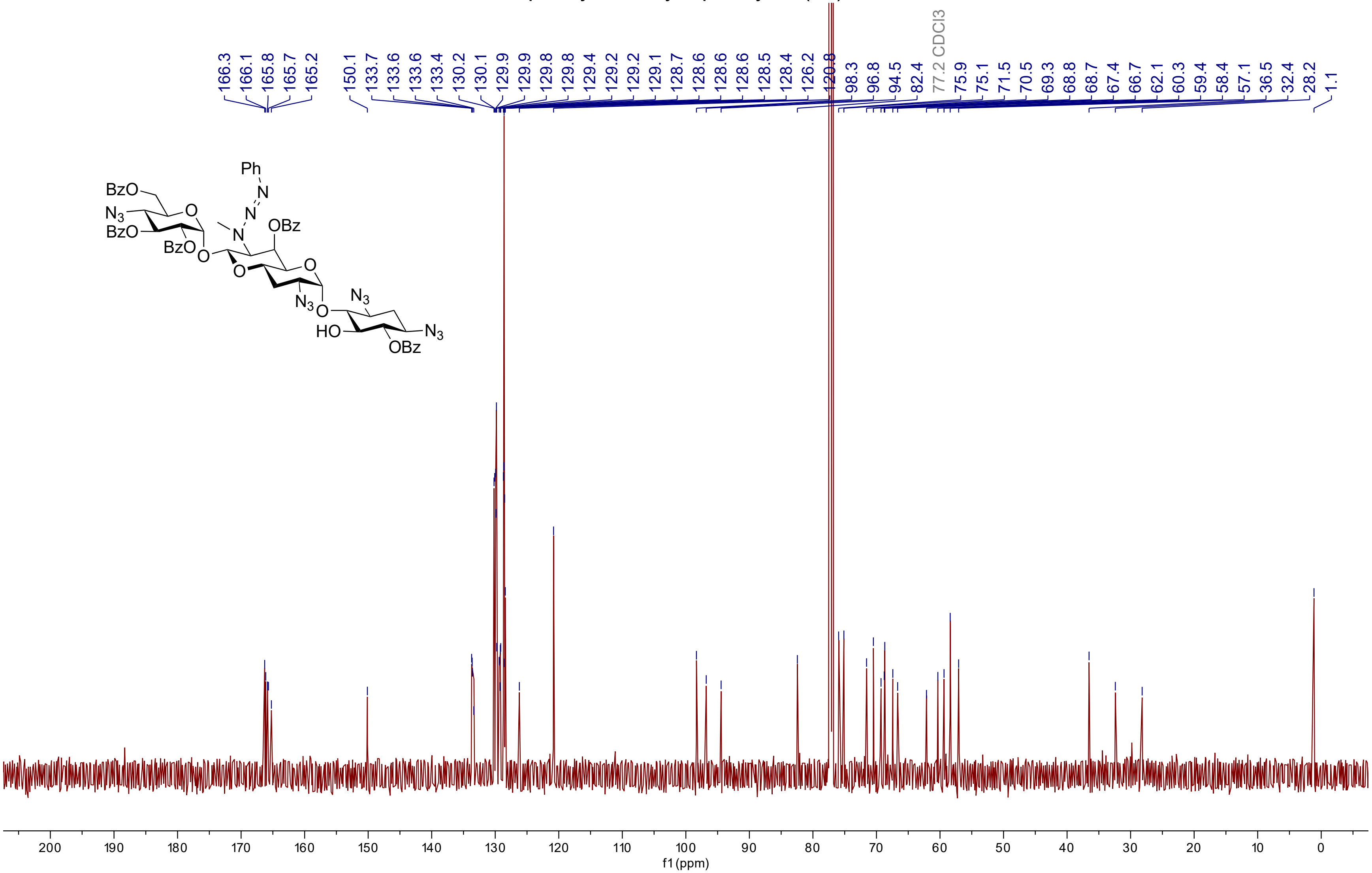

^{1}H-^{1}H COSY Spectrum (500 MHz, $CDCl_3$) of 1,3,2',4''-Tetra-azido-6,6',2'',3'',6''-penta-*O*-benzoyl-1,3,2',4''-tetra-(desamino)-7'-*N*-phenyldiazenyl-apramycin (**17**)

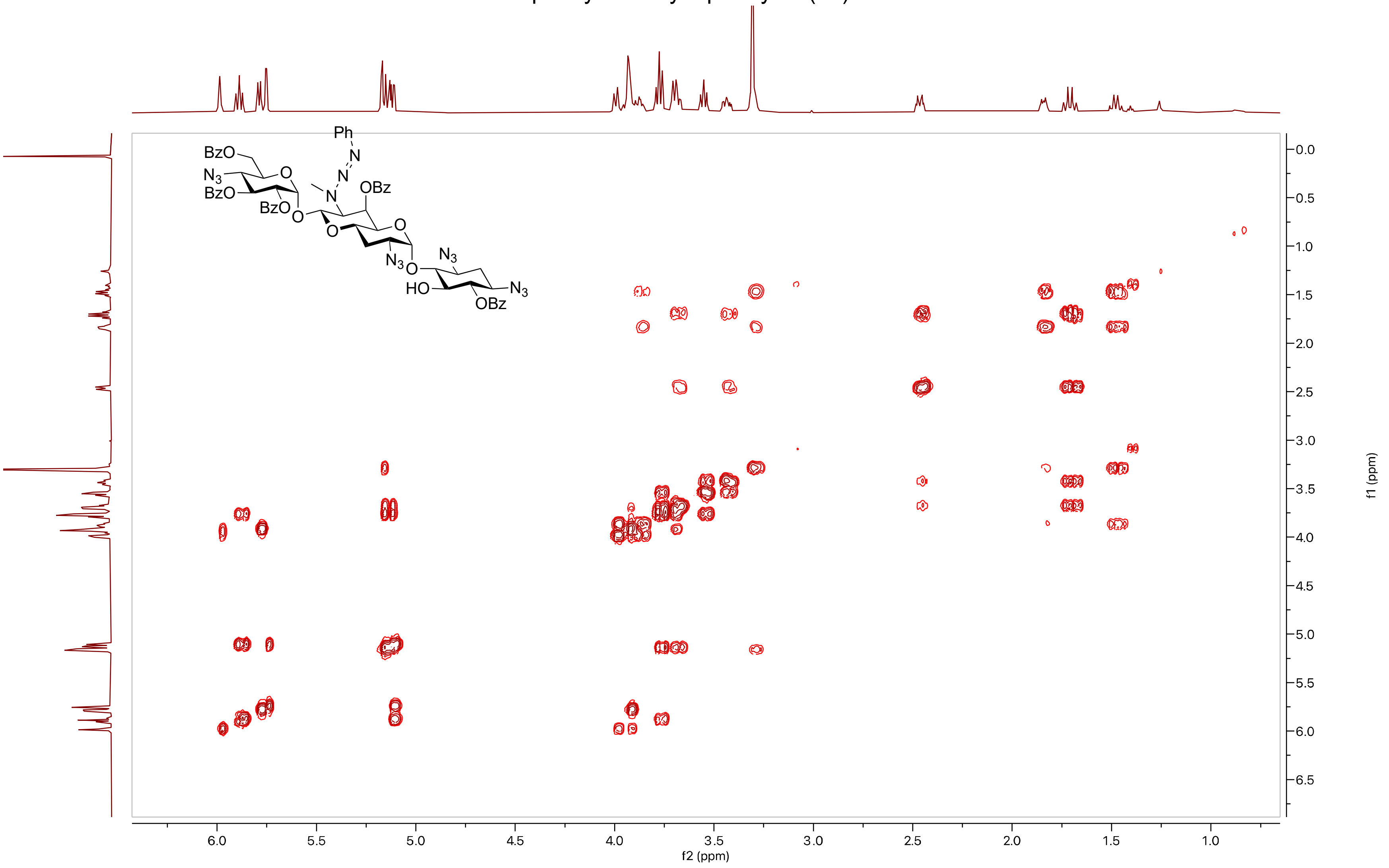

HSQC Spectrum (500 MHz, $CDCl_3$) of 1,3,2',4''-Tetra-azido-6,6',2'',3'',6''-penta-*O*-benzoyl-1,3,2',4''-tetra-(desamino)-7'-*N*-phenyldiazenyl-apramycin (**17**)

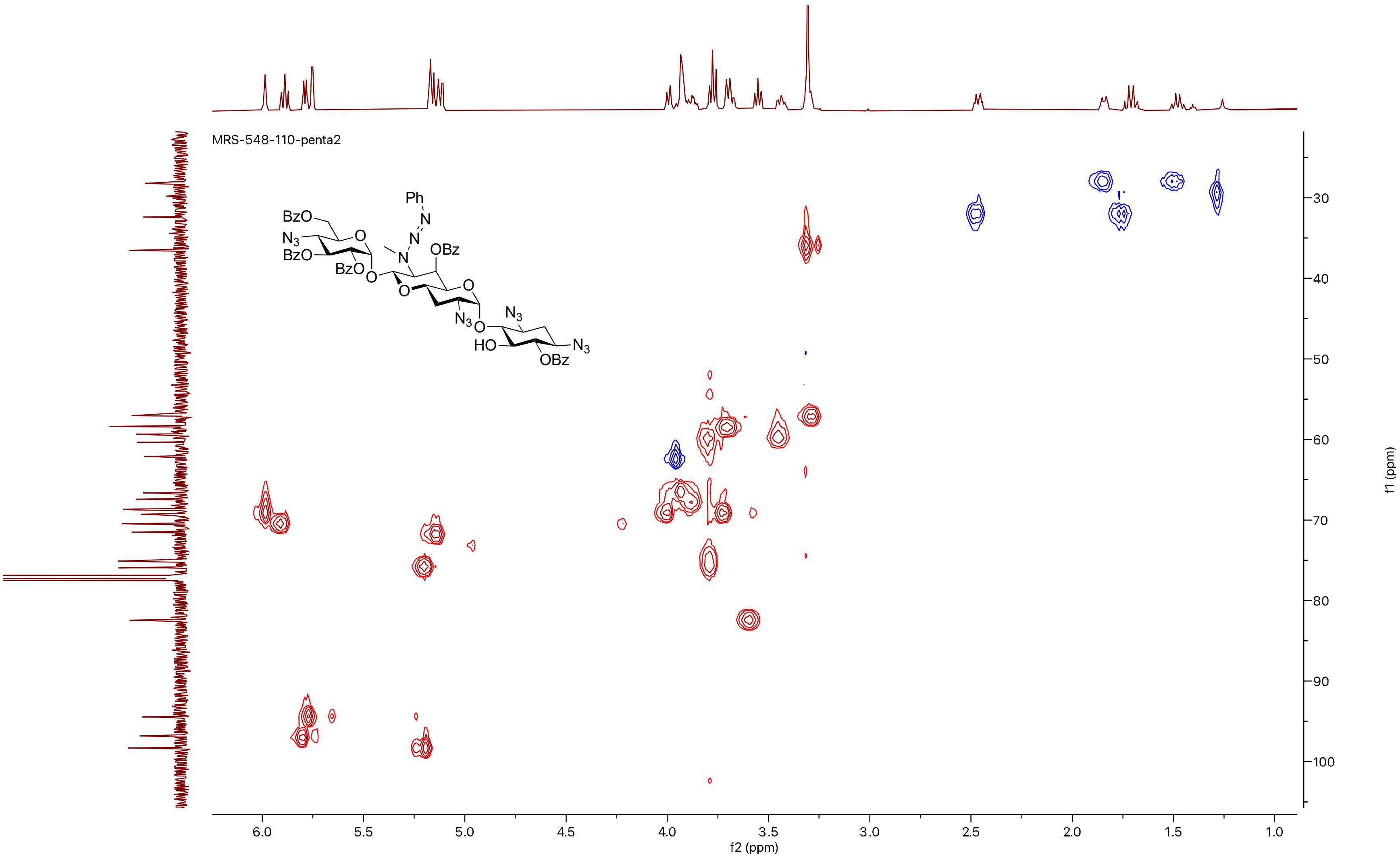

^{1}H NMR Spectrum (500 MHz, $CDCl_3$) of 1,3,2’,4’’-Tetra-azido-5,6’,2’’, 3’’,6’’-penta-*O*-benzoyl-1,3,2’,4’’-tetra-(desamino)- 7’-*N*-phenyldiazenyl-apramycin (**18**)

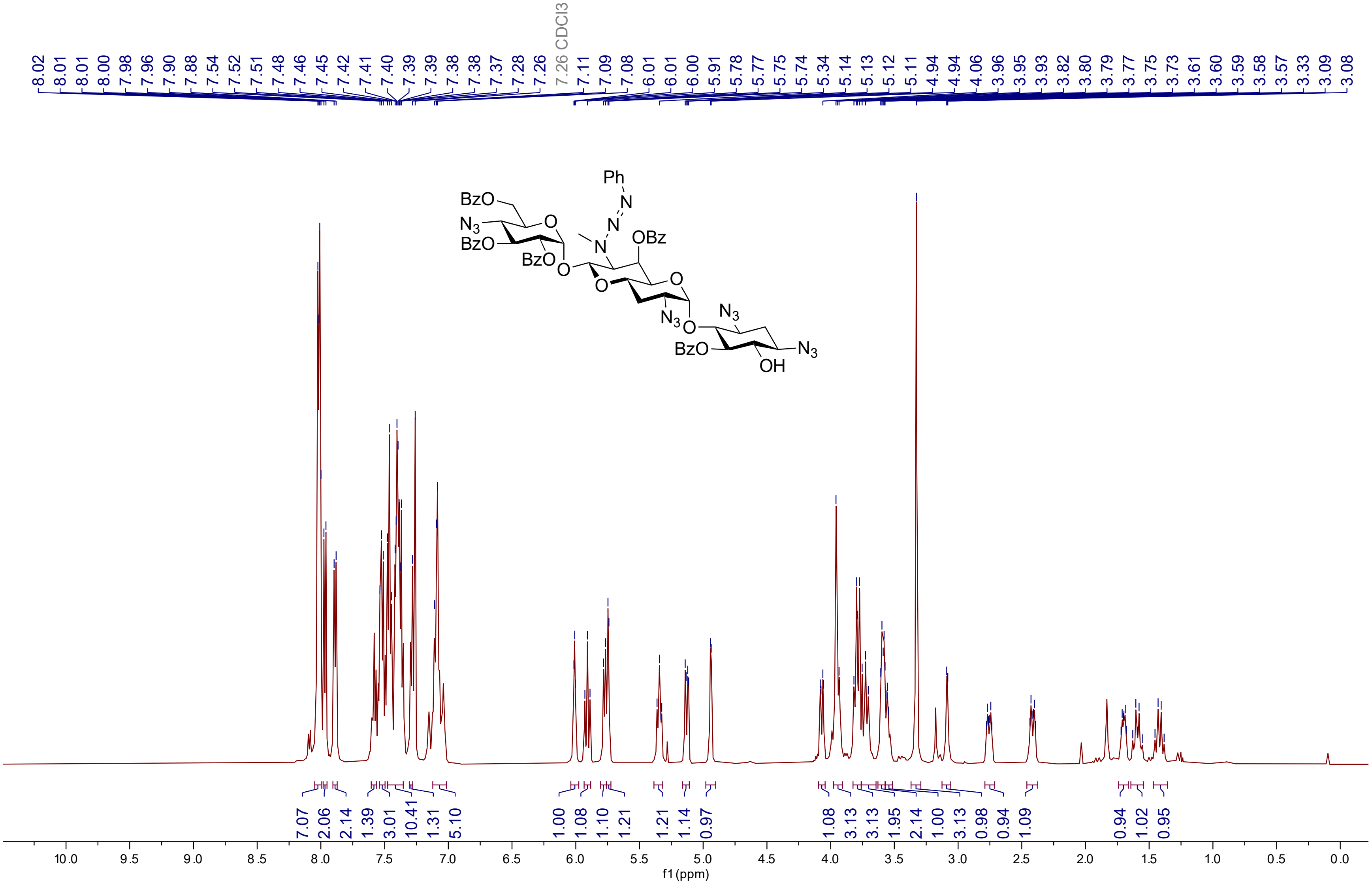

^{13}C NMR Spectrum (125 MHz, $CDCl_3$) of 1,3,2’,4’’-Tetra-azido-5,6’,2’’, 3’’,6’’-penta-*O*-benzoyl-1,3,2’,4’’-tetra-(desamino)-7’-*N*-phenyldiazenyl-apramycin (**18**)

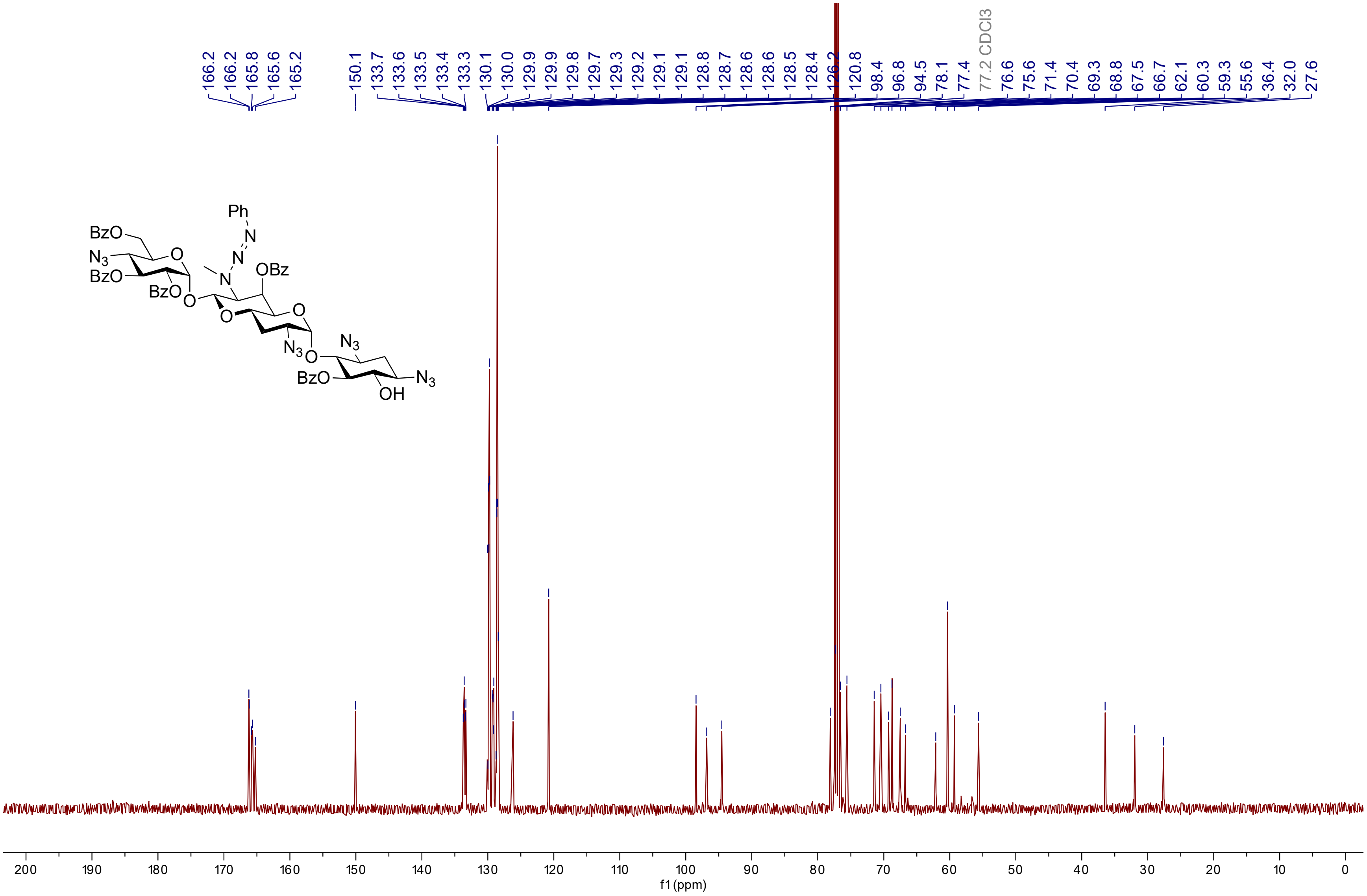

^{1}H-^{1}H COSY Spectrum (500 MHz, $CDCl_3$) of 1,3,2',4''-Tetra-azido-5,6',2'', 3'',6''-penta-*O*-benzoyl-1,3,2',4''-tetra-(desamino)- 7'-*N*-phenyldiazenyl-apramycin (**18**)

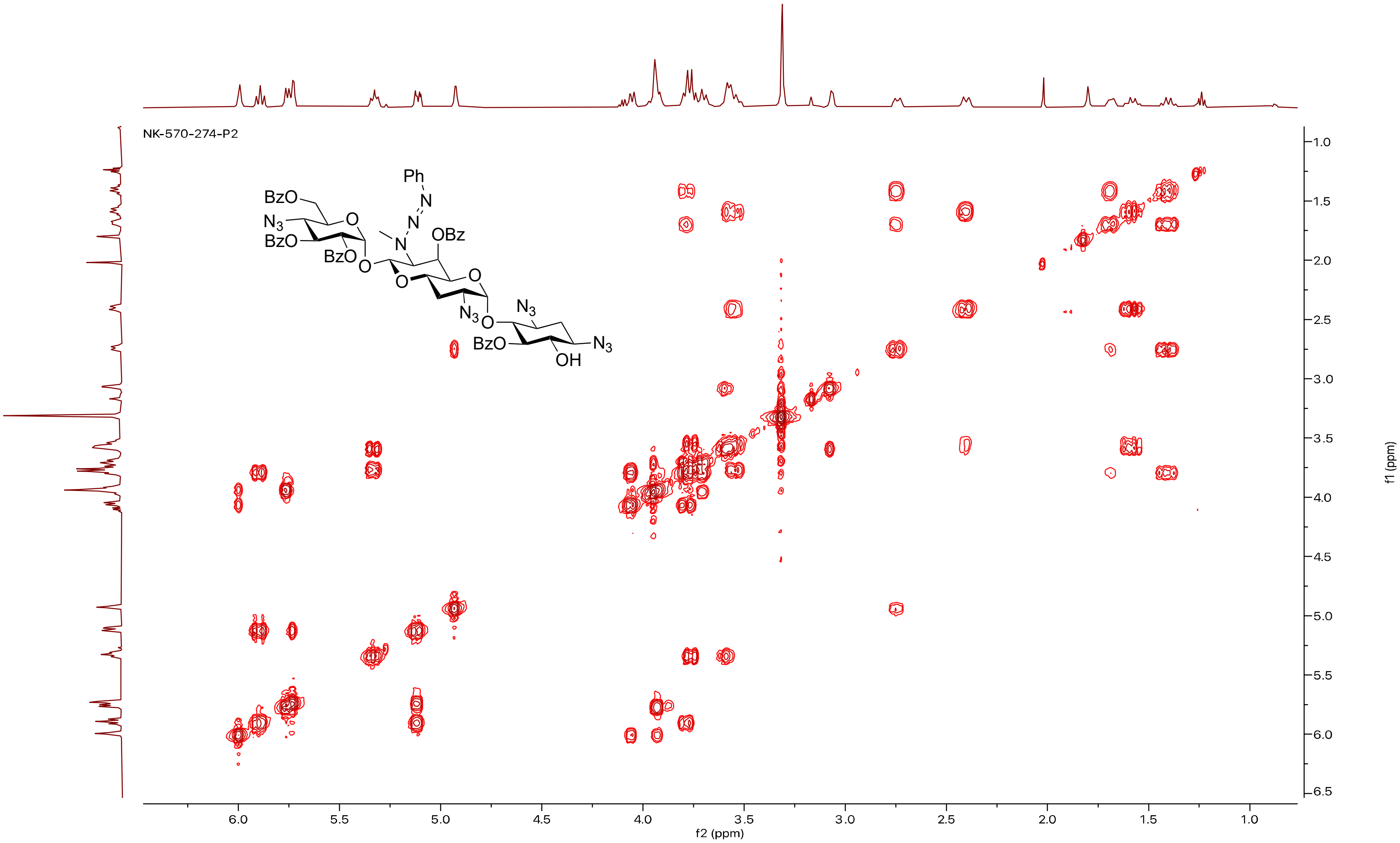

HSQC Spectrum (500 MHz, CDCl3) of 1,3,2',4''-Tetra-azido-5,6',2'', 3'',6''-penta-*O*-benzoyl-1,3,2',4''-tetra-(desamino)-7'-*N*-phenyldiazenyl-apramycin (**18**)

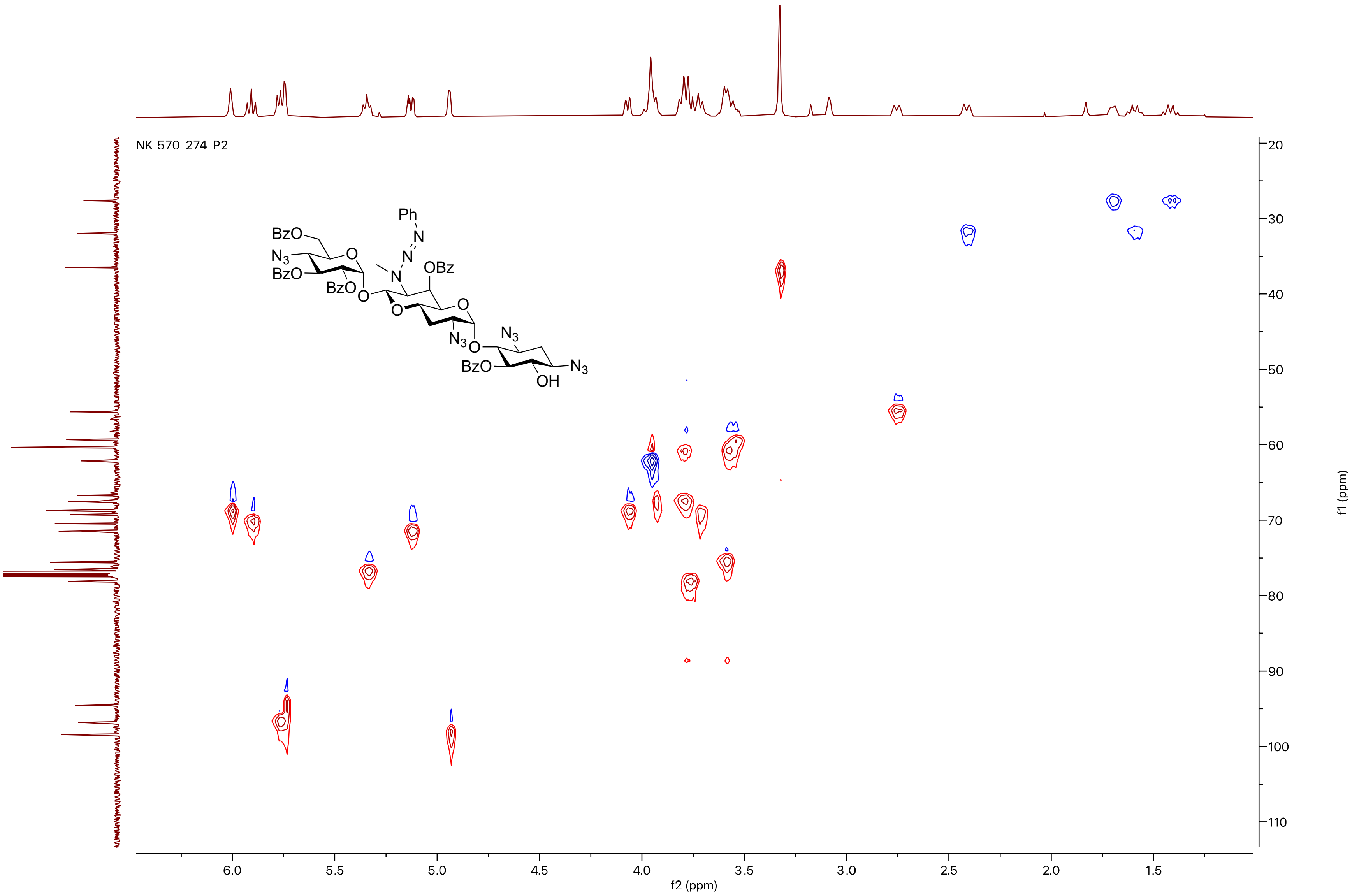

^{1}H NMR Spectrum (500 MHz, CDCl$_3$) of 1,3,2',4''-Tetra-azido-5,6',2'', 3'',6''-penta-*O*-benzoyl-1,3,2',4''-tetra-(desamino)-7'-*N*-phenyldiazenyl-apramycin-6-one (**19**)

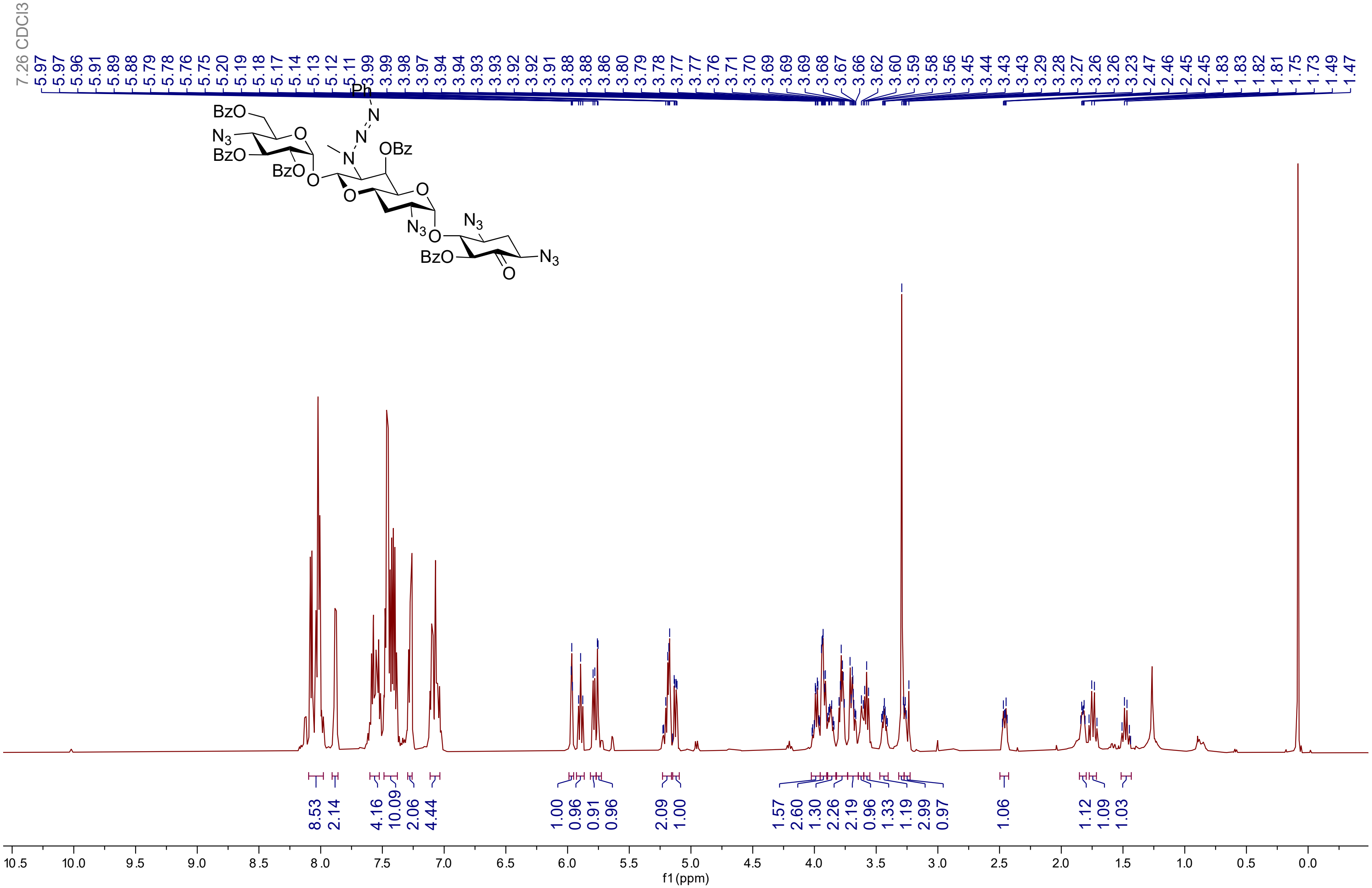

^{13}C NMR Spectrum (125 MHz, $CDCl_3$) of 1,3,2',4''-Tetra-azido-5,6',2'', 3'',6''-penta-*O*-benzoyl-1,3,2',4''-tetra-(desamino)-7'-*N*-phenyldiazenyl-apramycin-6-one (**19**)

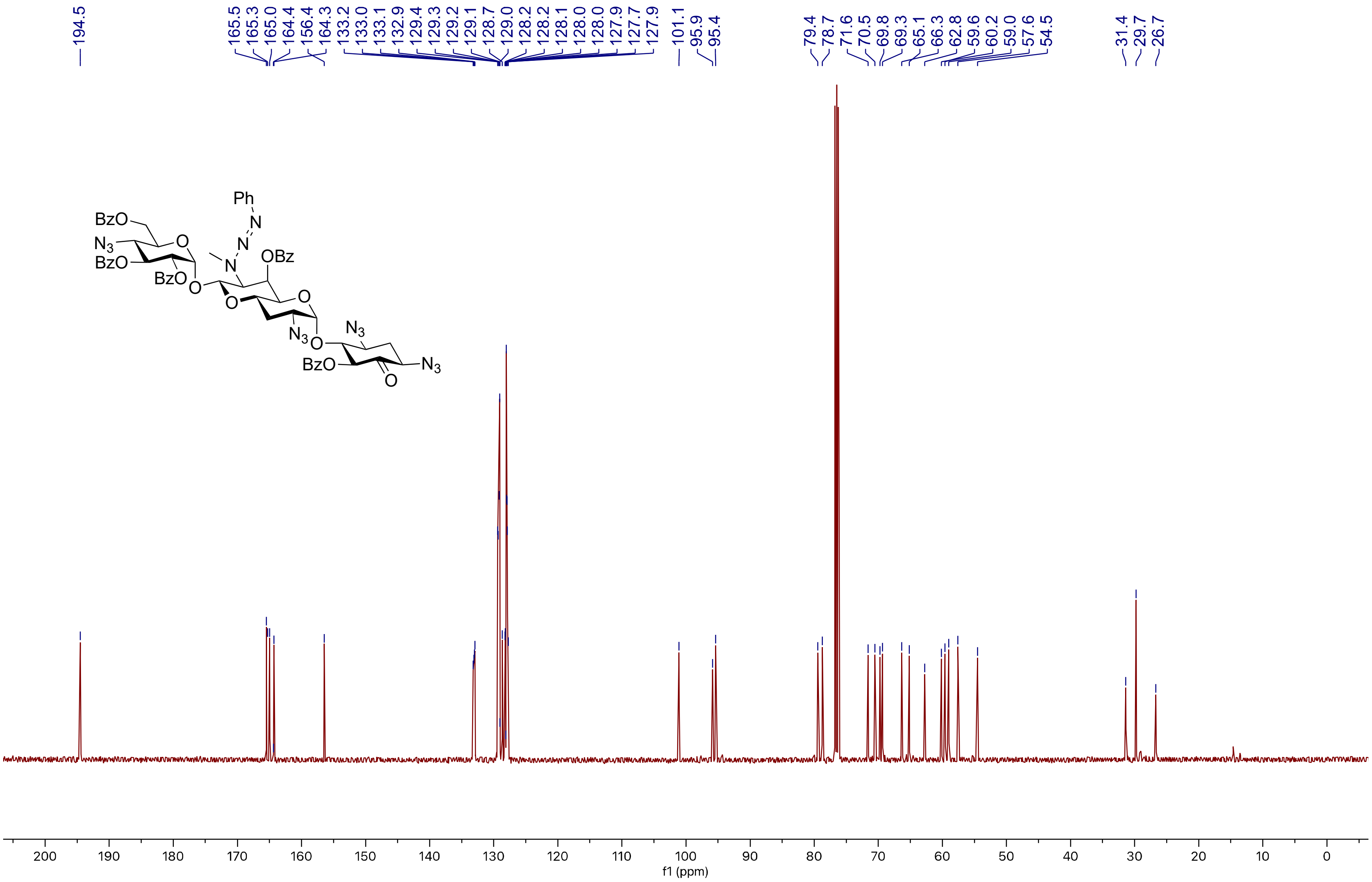

^{1}H-^{1}H COSY Spectrum (500 MHz, $CDCl_3$) of 1,3,2',4''-Tetra-azido-5,6',2'', 3'',6''-penta-*O*-benzoyl-1,3,2',4''-tetra-(desamino)-7'-*N*-phenyldiazenyl-apramycin-6-one (**19**)

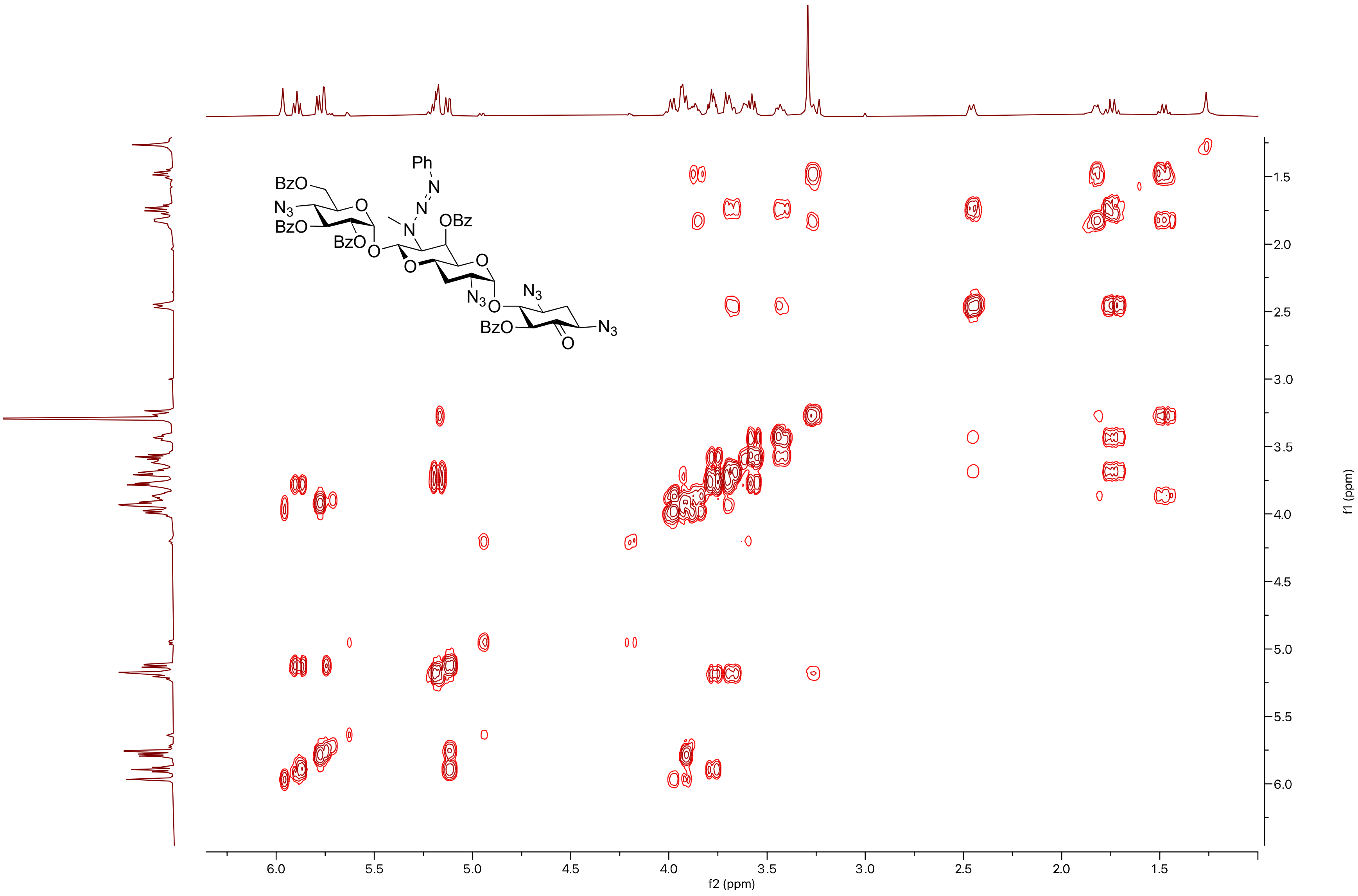

HSQC Spectrum (500 MHz, $CDCl_3$) of 1,3,2',4''-Tetra-azido-5,6',2'', 3'',6''-penta-*O*-benzoyl-1,3,2',4''-tetra-(desamino)-7'-*N*-phenyldiazenyl-apramycin-6-one (**19**)

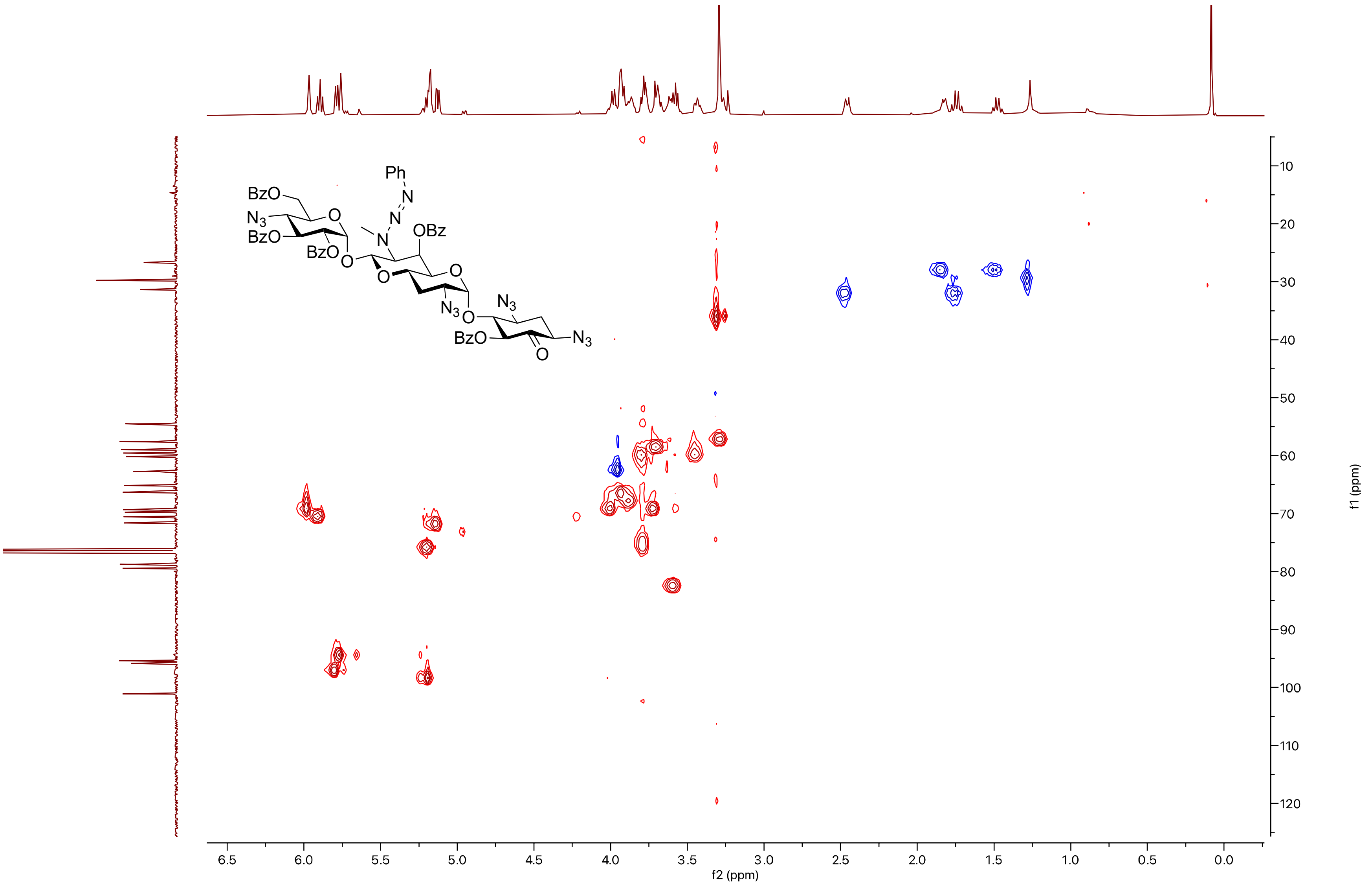

^{1}H NMR Spectrum (500 MHz, $CDCl_3$) of 2,4'-Di-azido-6,2', 3',6'-tetra-*O*-benzoyl-2,4'-di-(desamino)-7'-*N*-phenyldiazenyl-α,β-aprabiosamine (**20**)

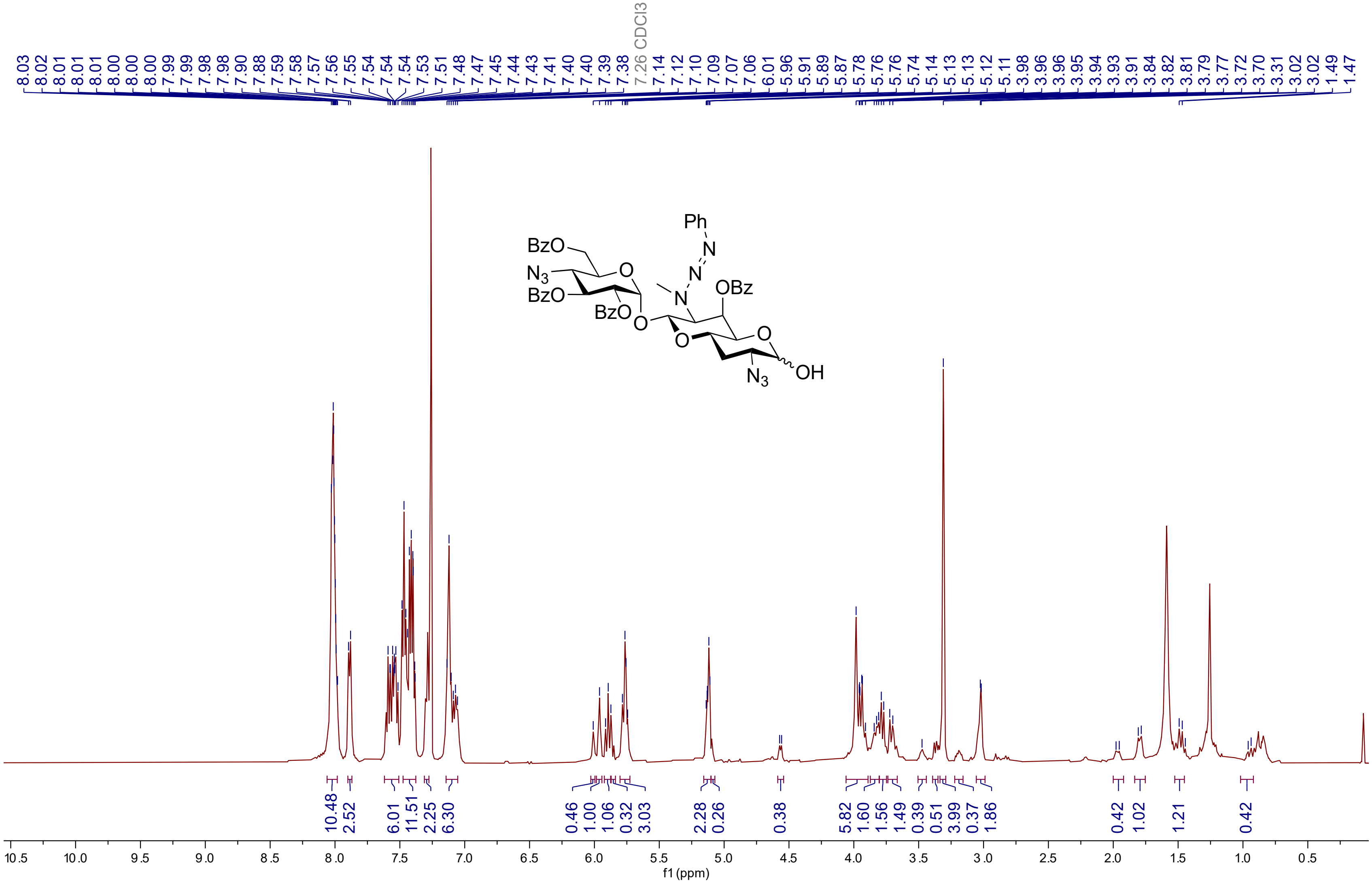

1D Selective Gradient TOCSY (600 MHz, $CDCl_3$) of the α-anomer of 2,4'-Di-azido-6,2', 3',6'-tetra-*O*-benzoyl-2,4'-di-(desamino)-7'-*N*-phenyldiazenyl-aprabiosamine (**20**)

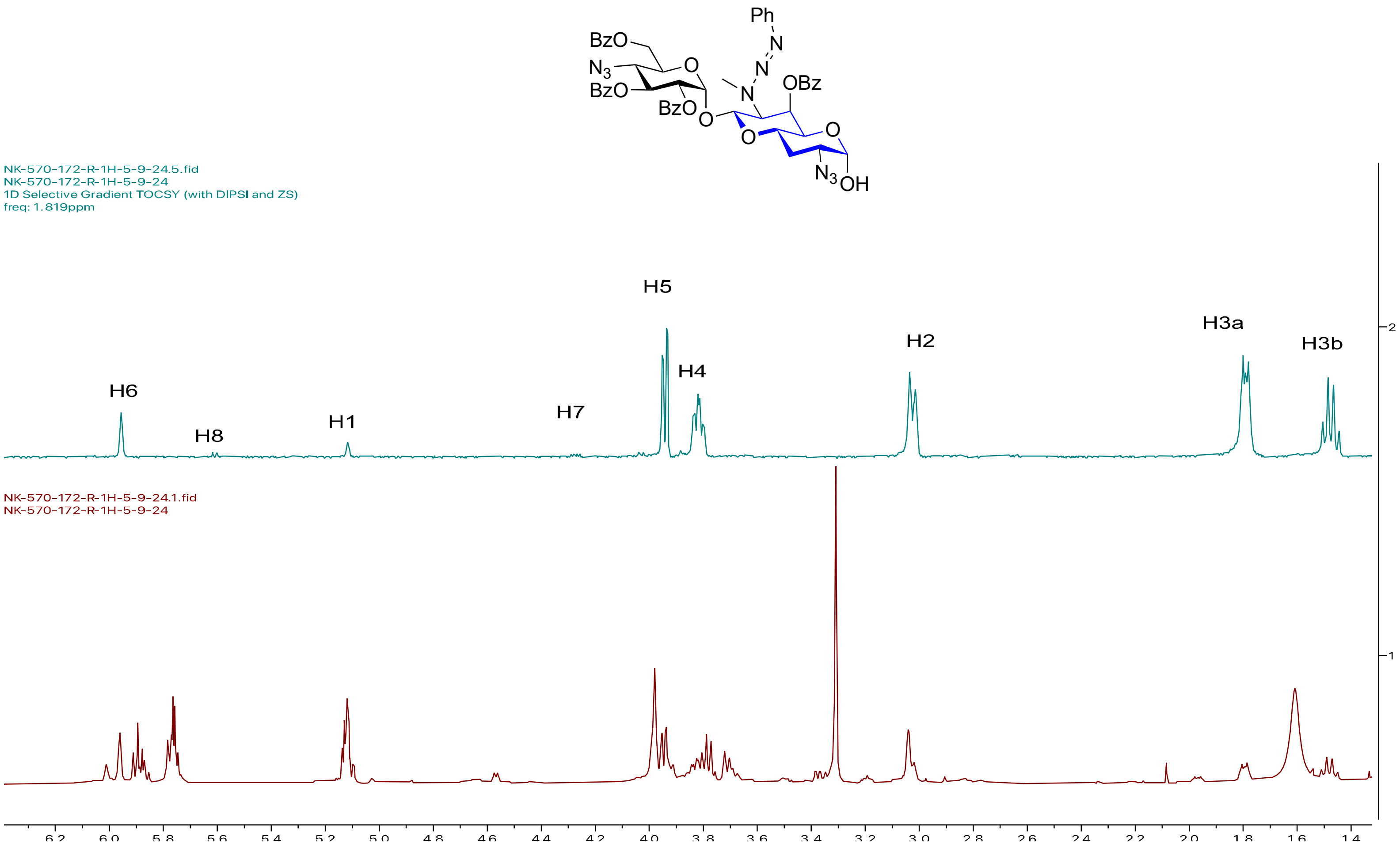

1D Selective gradient TOCSY (600 MHz, $CDCl_3$) of the β-anomer of 2,4'-Di-azido-6,2', 3',6'-tetra-*O*-benzoyl-2,4'-di-(desamino)-7'-*N*-phenyldiazenyl-aprabiosamine (**20**)

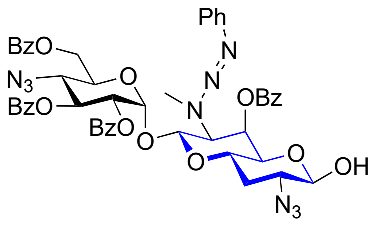

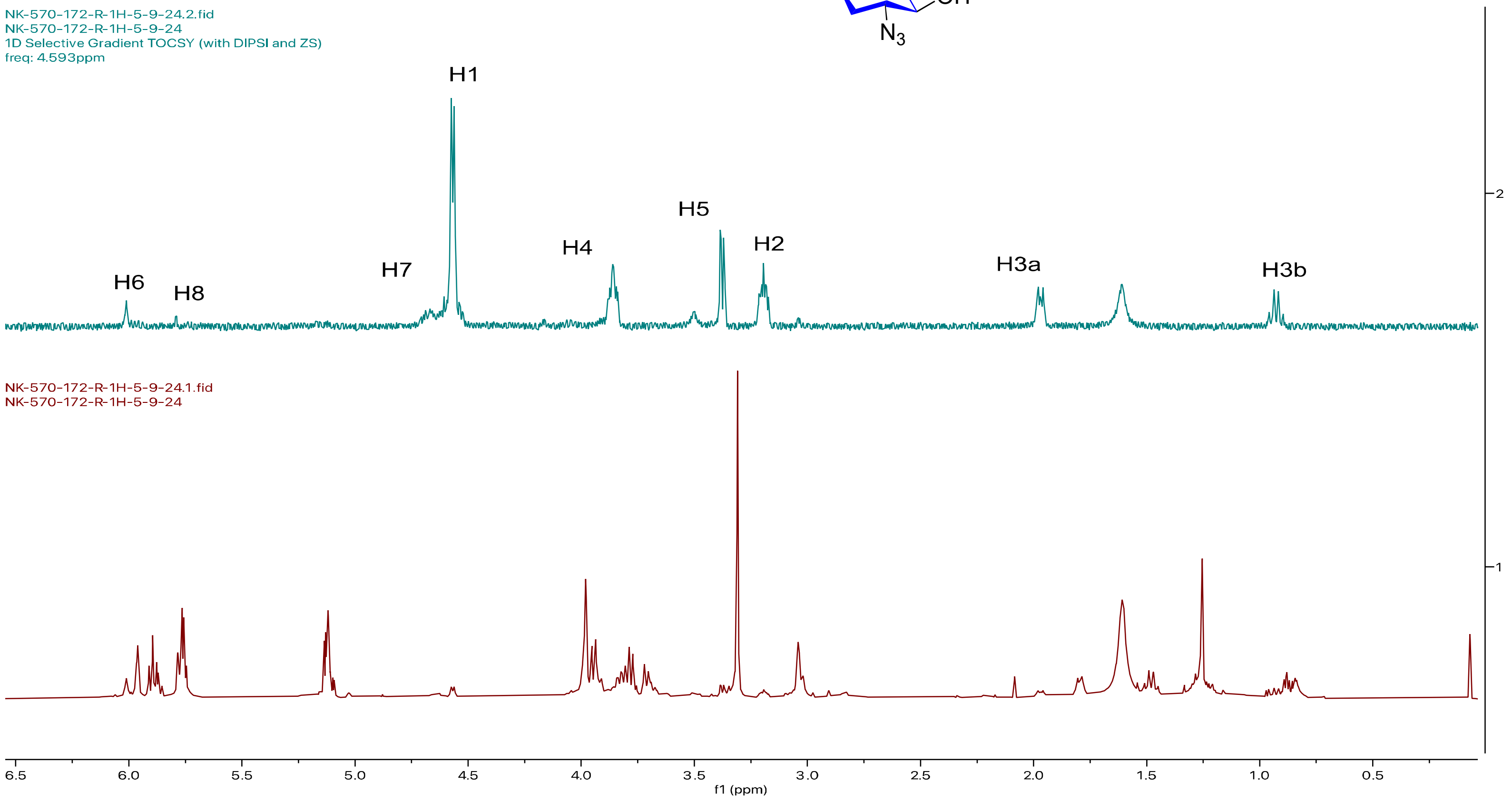

^{13}C NMR Spectrum (500 MHz, $CDCl_3$) of 2,4'-Di-azido-6,2',3',6'-tetra-*O*-benzoyl-2,4'-di-(desamino)-7'-*N*-phenyldiazenyl-α,β-aprabiosamine (**20**)

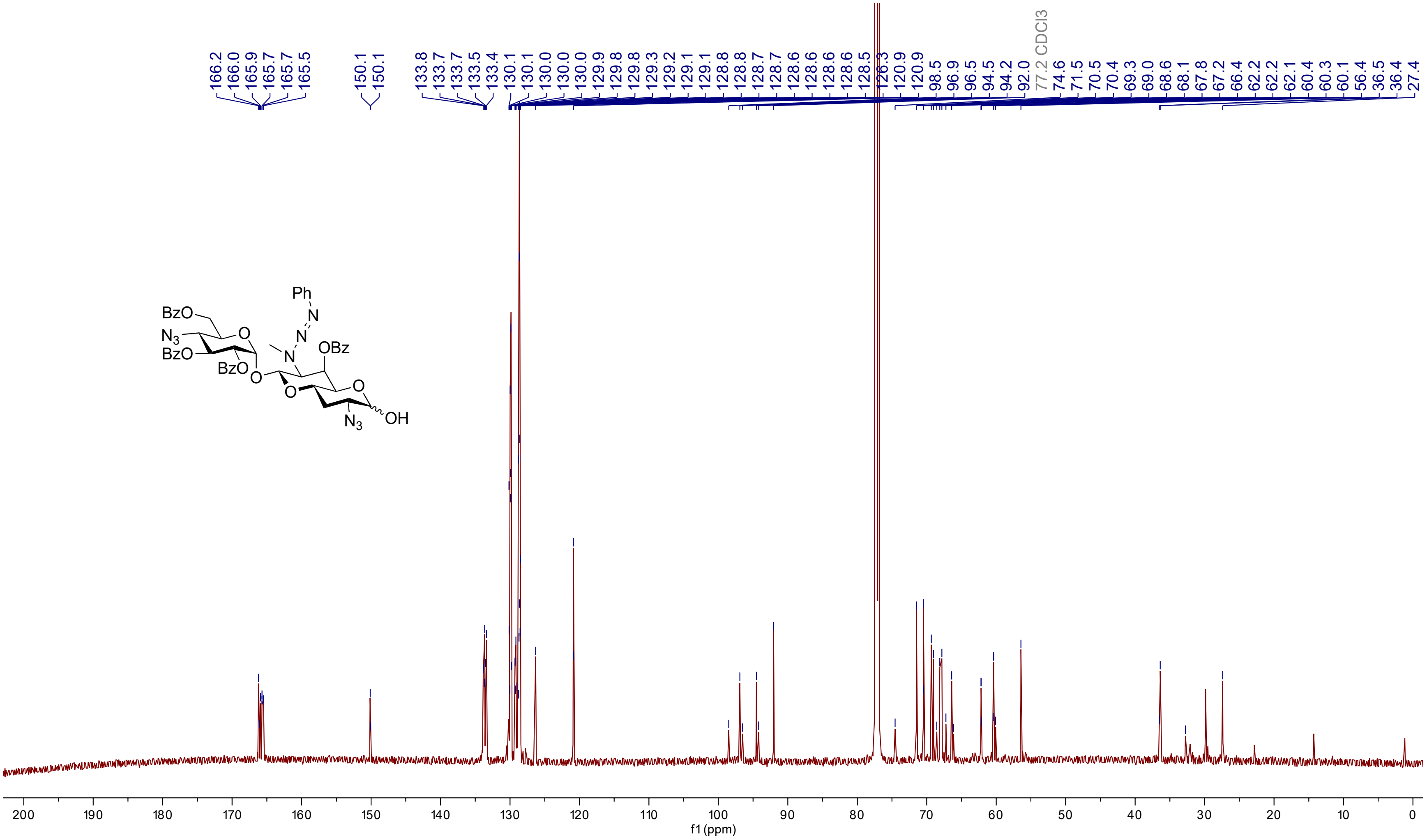

HSQC Spectrum (500 MHz, $CDCl_3$) of 2,4'-Di-azido-6,2', 3',6'-tetra-*O*-benzoyl-2,4'-di-(desamino)-7'-*N*-phenyldiazenyl-α,β-aprabiosamine (**20**)

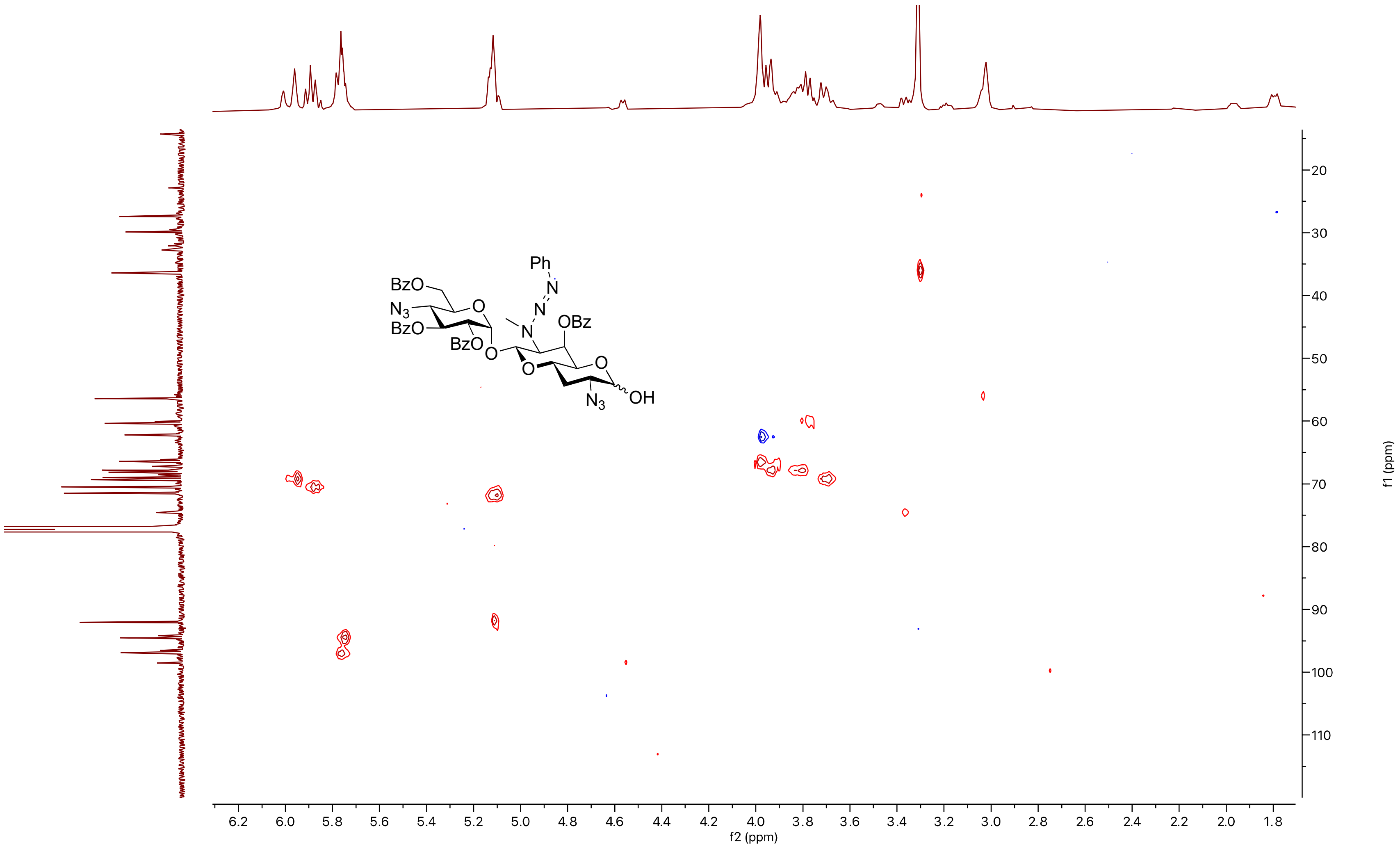

^{1}H NMR Spectrum (500 MHz, $CDCl_3$) of *N*-Phenyltrifluoroacetamidoyl 2,4'-di-azido-6,2', 3',6'-tetra-*O*-benzoyl-2,4'-di-(desamino)-7'-*N*-phenyldiazenyl-α,β-aprabiosaminide (**21**)

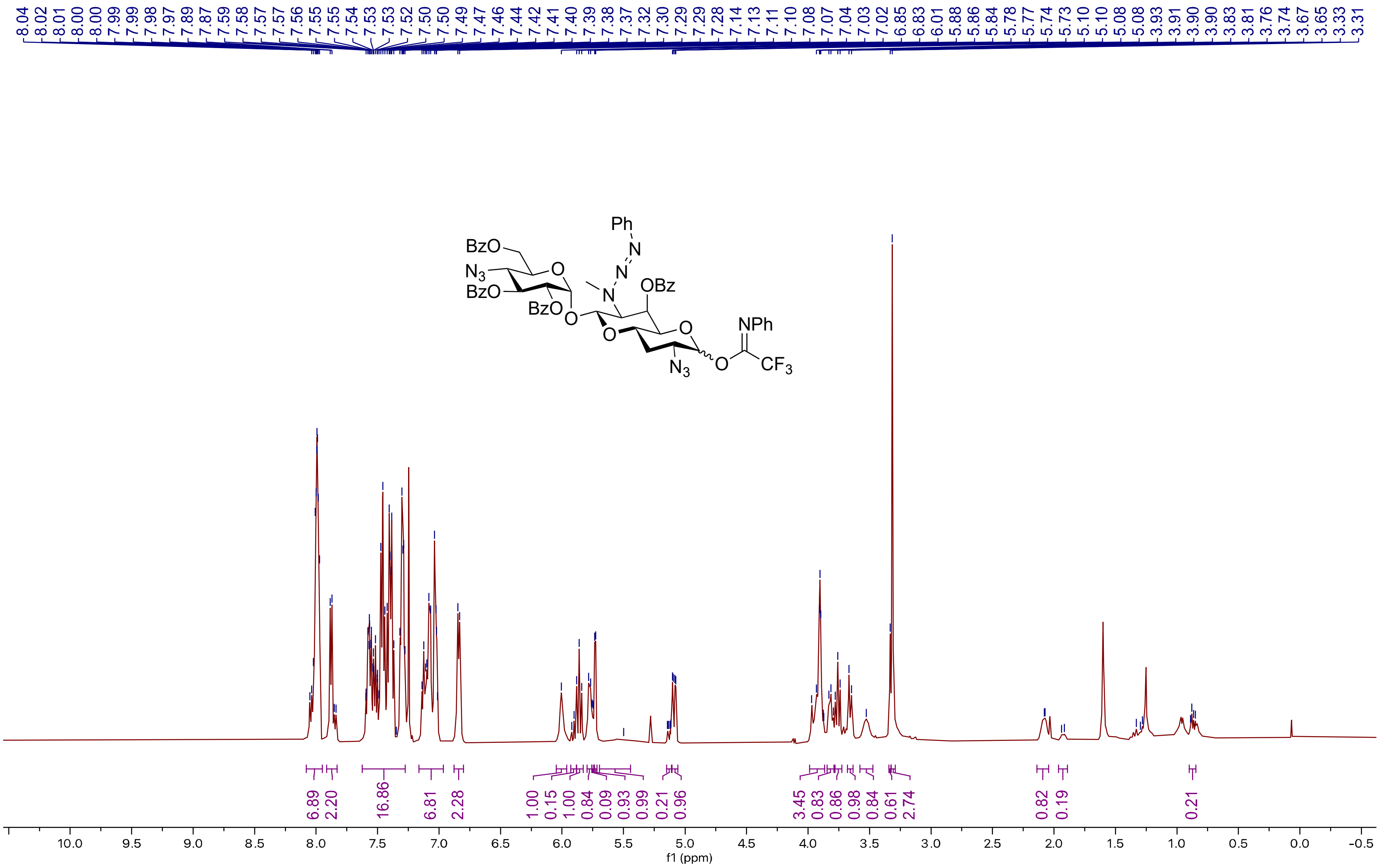

^{13}C NMR Spectrum (125 MHz, CDCl$_3$) of *N*-Phenyltrifluoroacetamidoyl 2,4'-di-azido-6,2', 3',6'-tetra-*O*-benzoyl-2,4'-di-(desamino)-7'-*N*-phenyldiazenyl-α,β-aprabiosaminide (**21**)

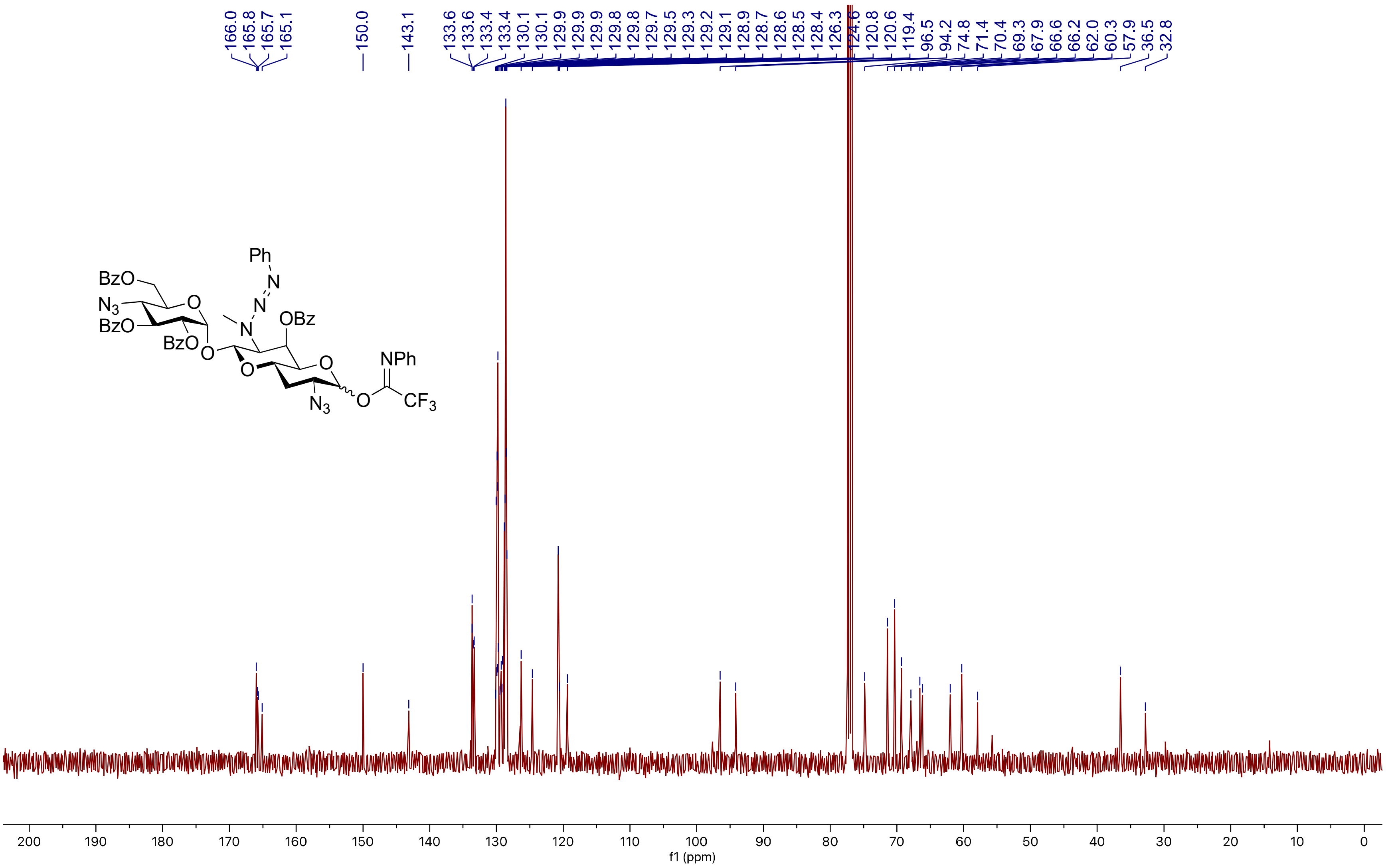

^{1}H-^{1}H COSY Spectrum (500 MHz, $CDCl_3$) of *N*-Phenyltrifluoroacetamidoyl 2,4'-di-azido-6,2', 3',6'-tetra-*O*-benzoyl-2,4'-di-(desamino)-7'-*N*-phenyldiazenyl-α,β-aprabiosaminide (**21**)

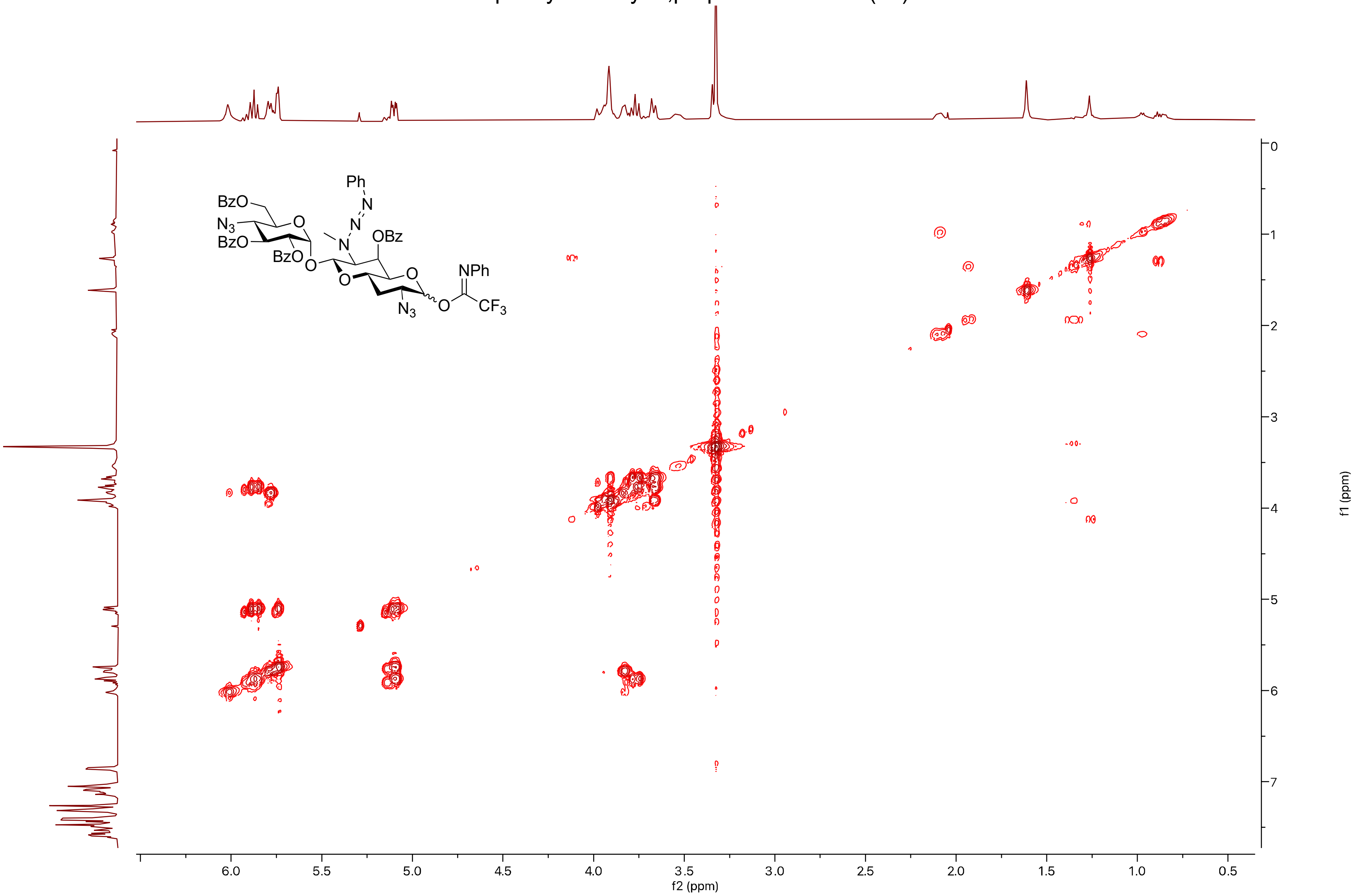

HSQC Spectrum (500 MHz, $CDCl_3$) of *N*-Phenyltrifluoroacetamidoyl 2,4'-di-azido-6,2',3',6'-tetra-*O*-benzoyl-2,4'-di-(desamino)-7'-*N*-phenyldiazenyl-α,β-aprabiosaminide (**21**)

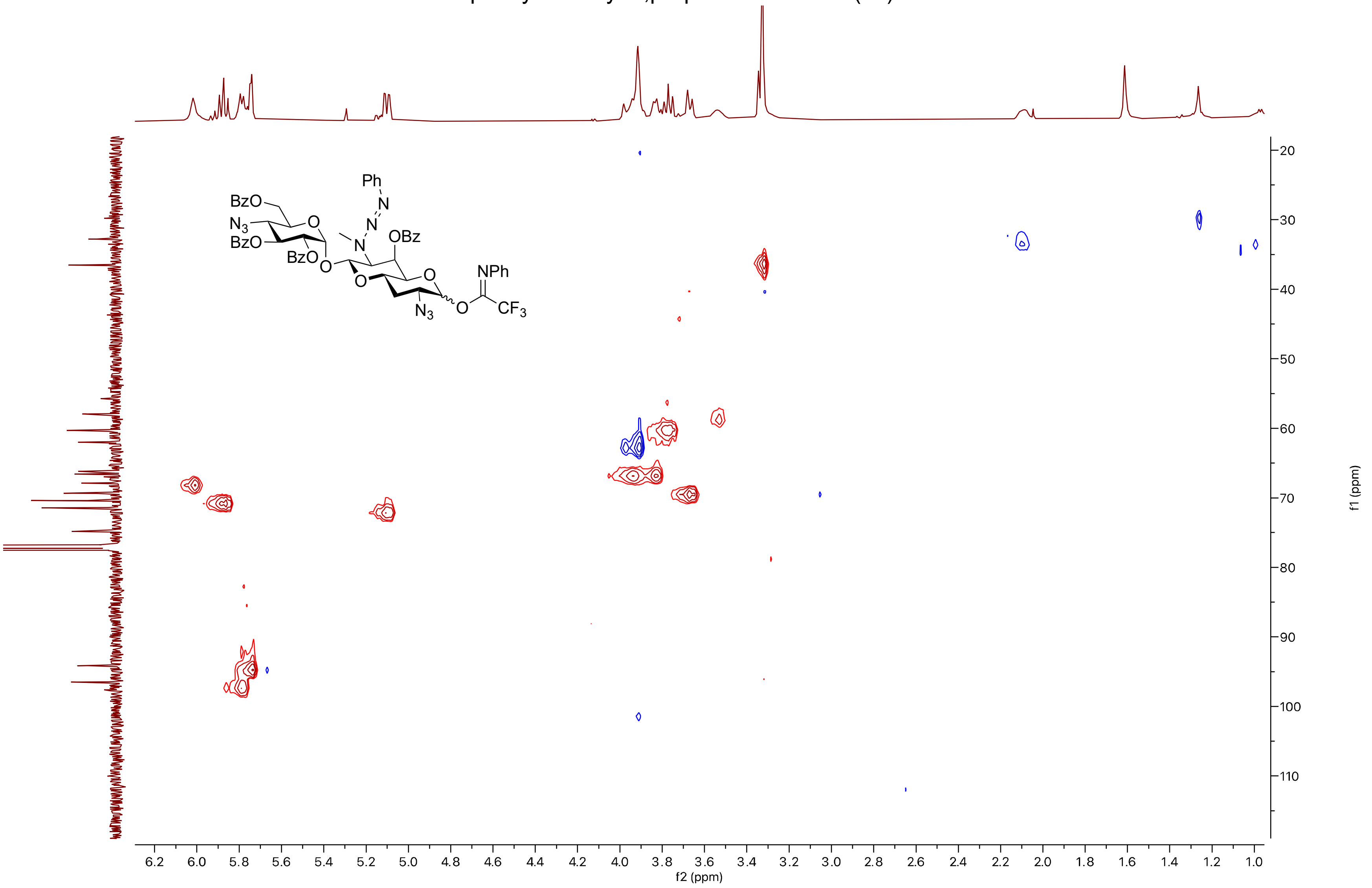

^{1}H NMR Spectrum (500 MHz, $CDCl_3$) of 1,3,2′,4″-Tetra-azido-6,2″,3″,6″-tetra-*O*-benzoyl-1,3,2′,4″-tetra-(desamino)-6′,7′-oxazolidino-apramycin-5-one (**26**)

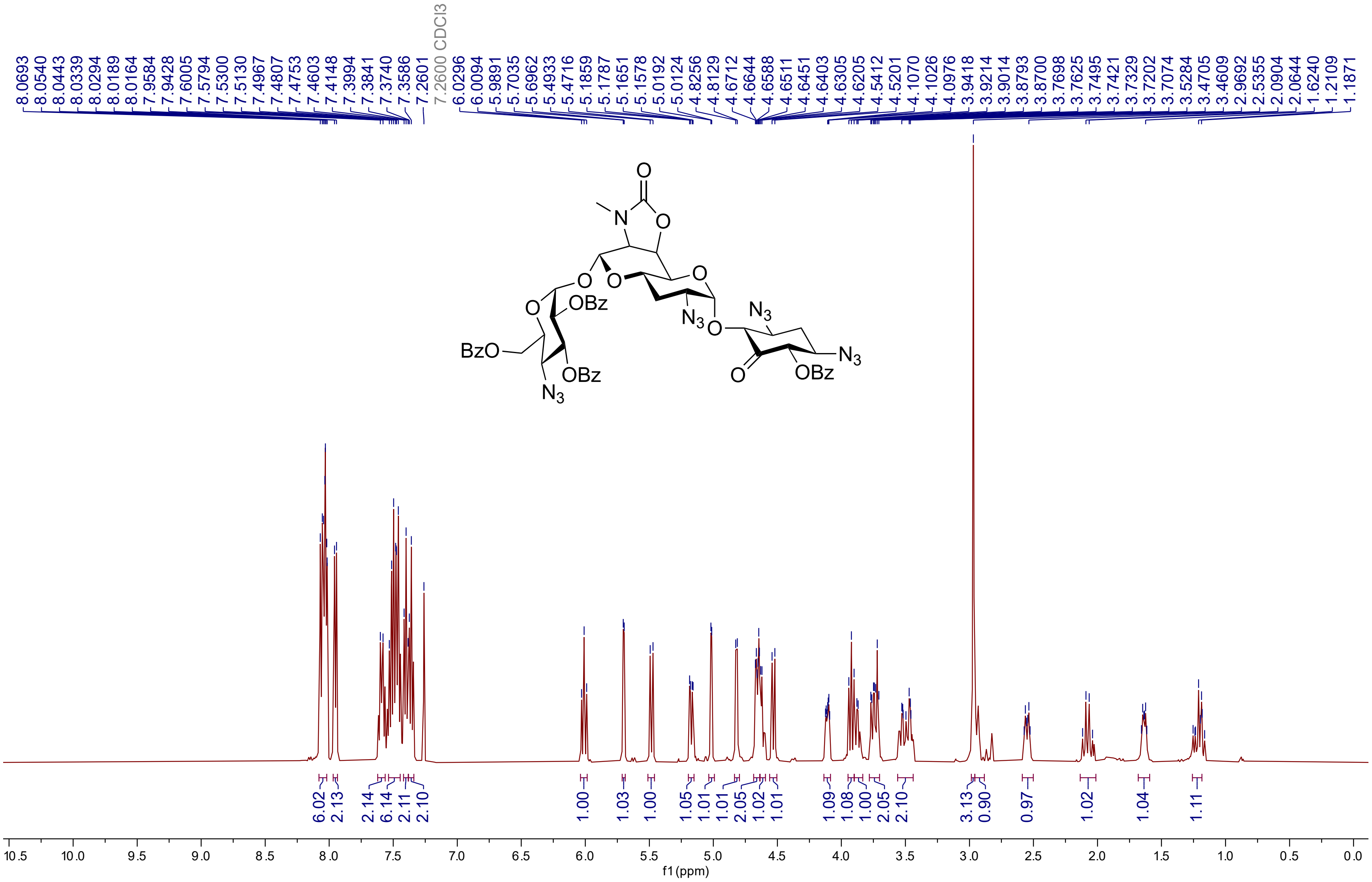

^{13}C NMR Spectrum (125 MHz, $CDCl_3$) of 1,3,2′,4″-Tetra-azido-6,2″,3″,6″-tetra-*O*-benzoyl-1,3,2′,4″-tetra-(desamino)-6′,7′-oxazolidino-apramycin-5-one (**26**)

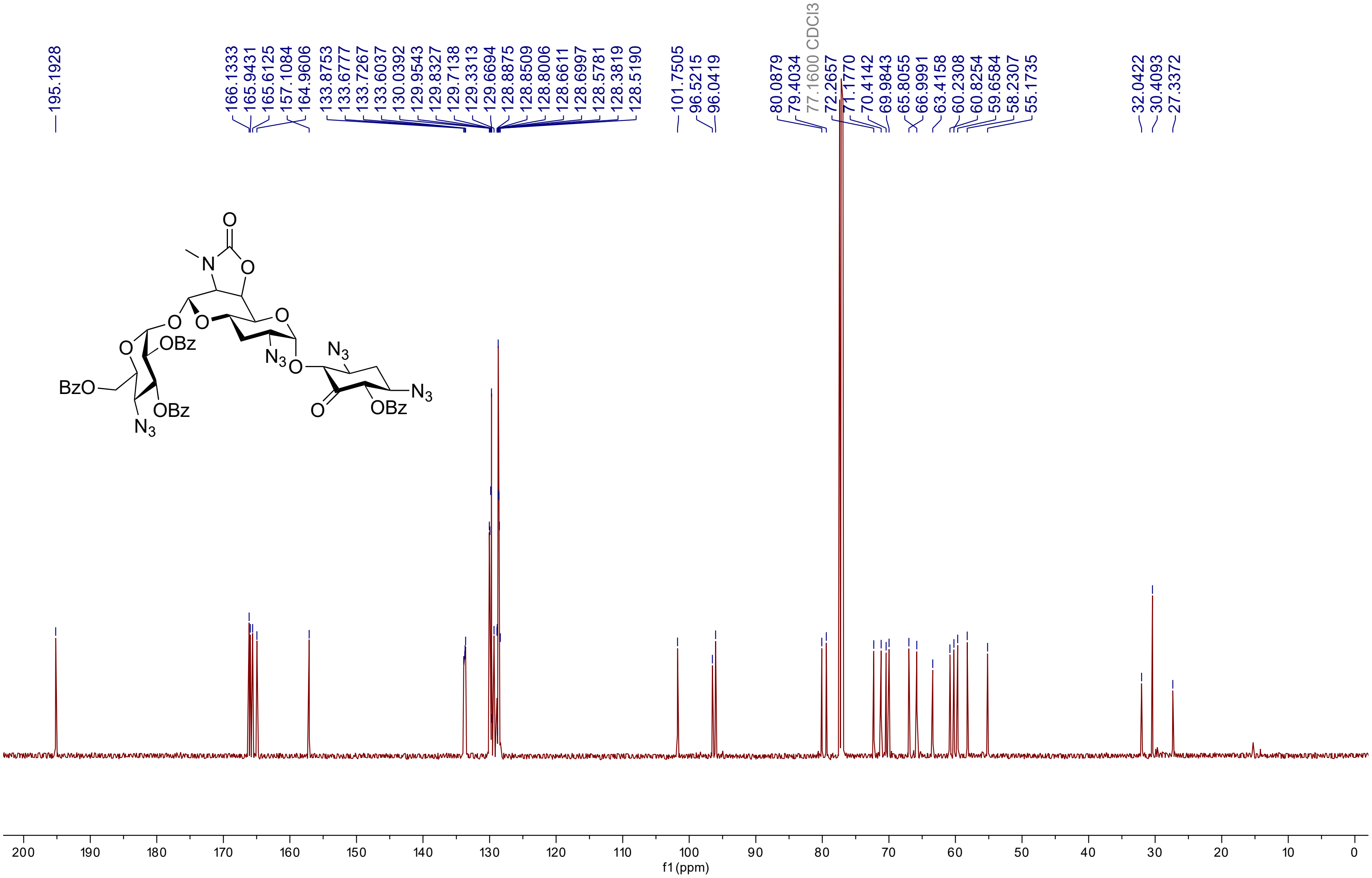

^{1}H-^{1}H COSY Spectrum (500 MHz, $CDCl_3$) of 1,3,2′,4″-Tetra-azido-6,2″,3″,6″-tetra-*O*-benzoyl-1,3,2′,4″-tetra-(desamino)-6′,7′-oxazolidino-apramycin-5-one (**26**)

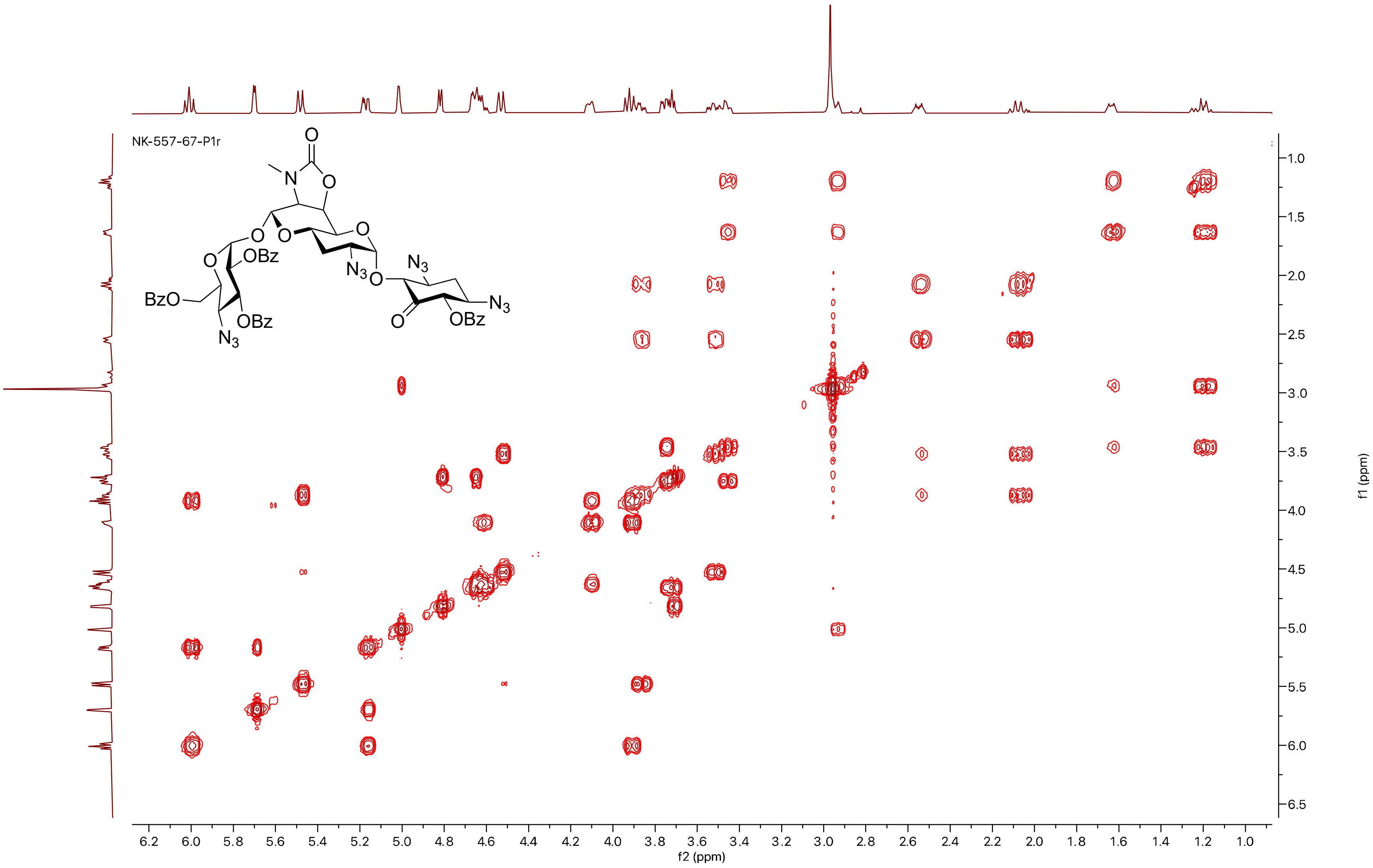

HSQC Spectrum (500 MHz, $CDCl_3$) of 1,3,2′,4″-Tetra-azido-6,2″,3″,6″-tetra-*O*-benzoyl-1,3,2′,4″-tetra-(desamino)-6′,7′-oxazolidino-apramycin-5-one (**26**)

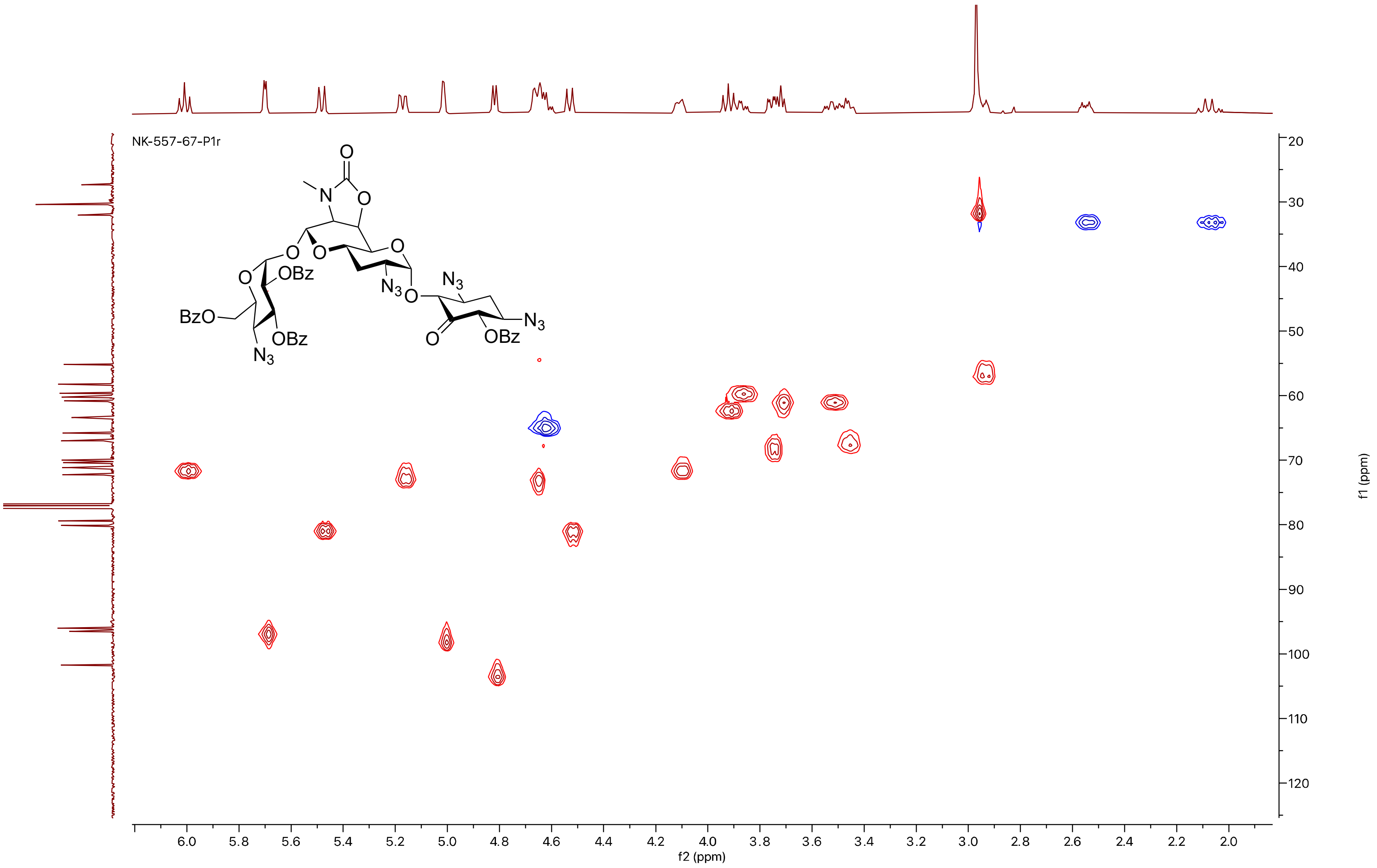

^{1}H NMR Spectrum (500 MHz, $CDCl_3$) of 1,3,2′,4″-Tetra-azido-5,2″,3″,6″-tetra-*O*-benzoyl-1,3,2′,4″-tetra-(desamino)-6′,7′-oxazolidino-apramycin-6-one (**27**)

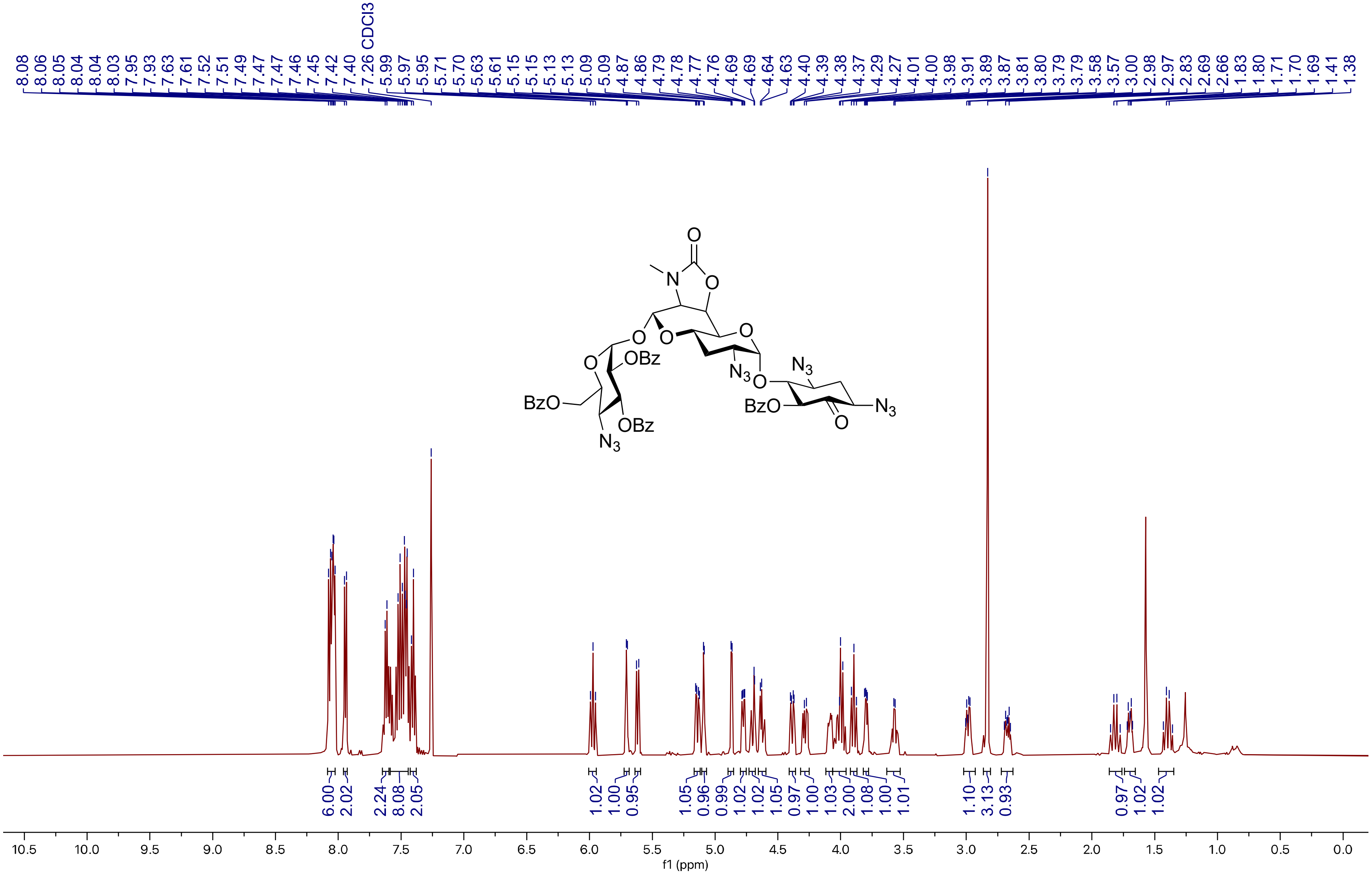

^{13}C NMR Spectrum (125 MHz, $CDCl_3$) of 1,3,2′,4″-Tetra-azido-5,2″,3″,6″-tetra-*O*-benzoyl-1,3,2′,4″-tetra-(desamino)-6′,7′-oxazolidino-apramycin-6-one (**27**)

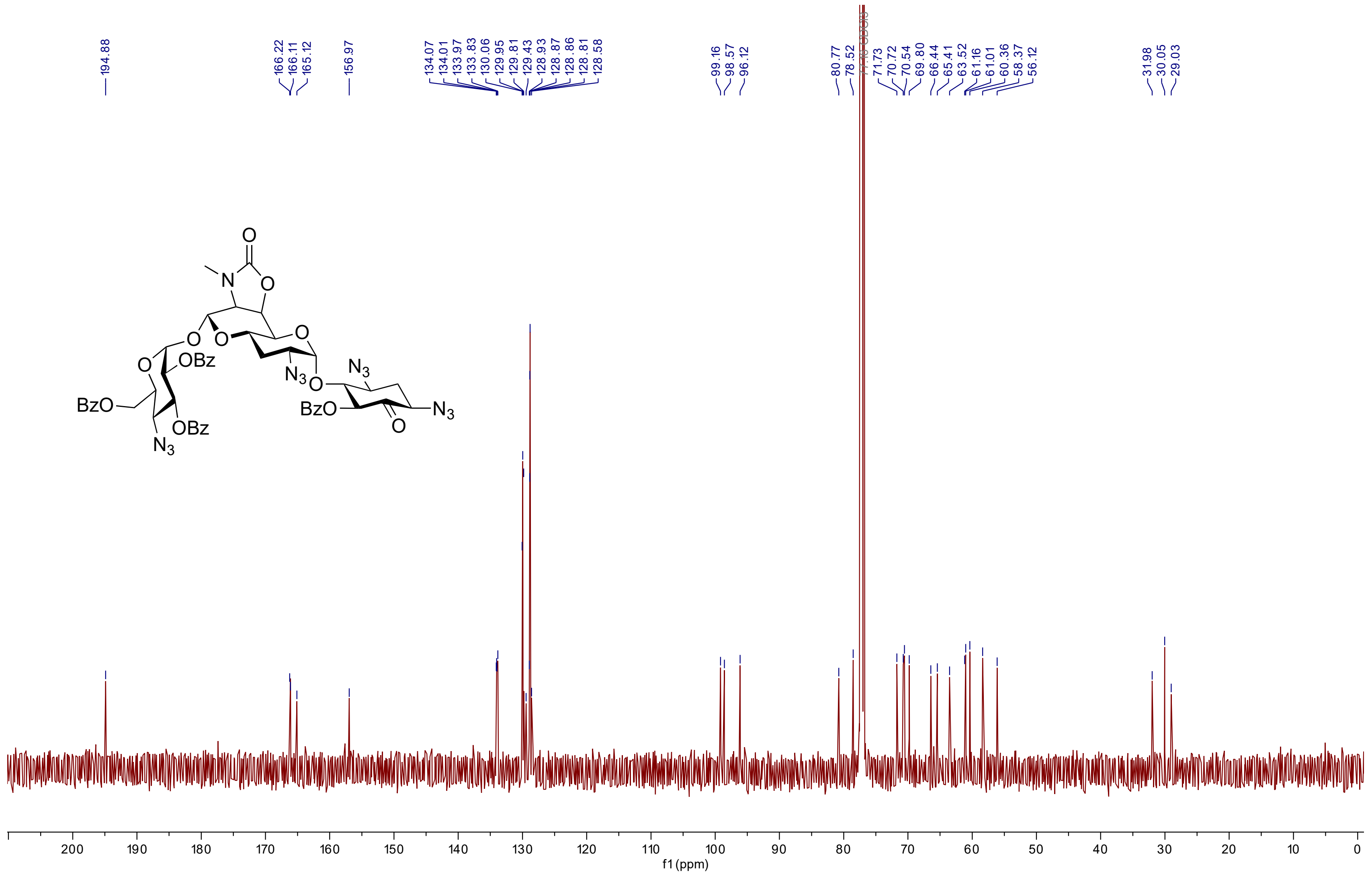

^{1}H-^{1}H COSY Spectrum (500 MHz, $CDCl_3$) of 1,3,2′,4″-Tetra-azido-5,2″,3″,6″-tetra-*O*-benzoyl-1,3,2′,4″-tetra-(desamino)-6′,7′-oxazolidino-apramycin-6-one (**27**)

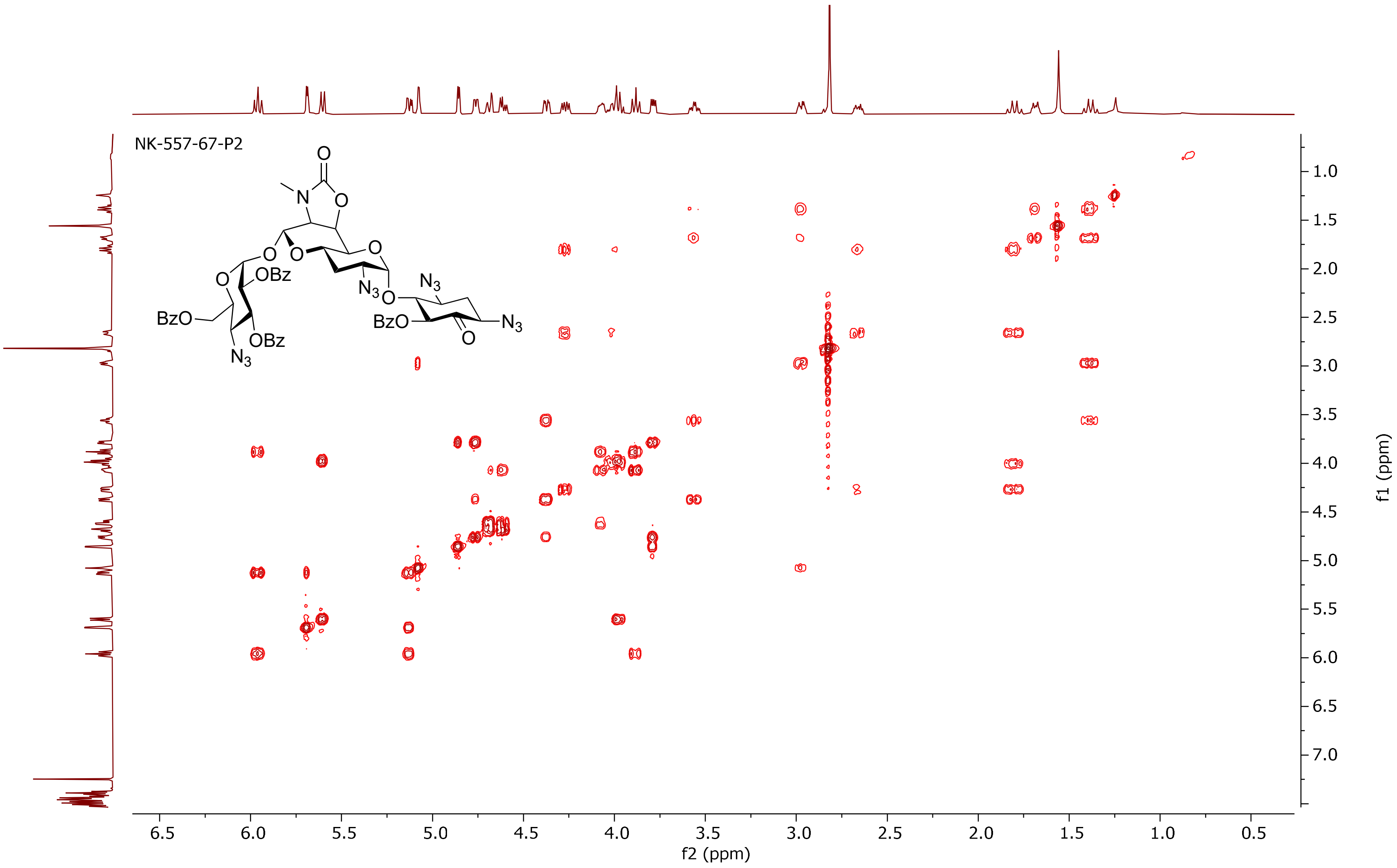

HSQC Spectrum (500 MHz, CDCl₃) of 1,3,2′,4″-Tetra-azido-5,2″,3″,6″-tetra-*O*-benzoyl-1,3,2′,4″-tetra-(desamino)-6′,7′-oxazolidino-apramycin-6-one (**27**)

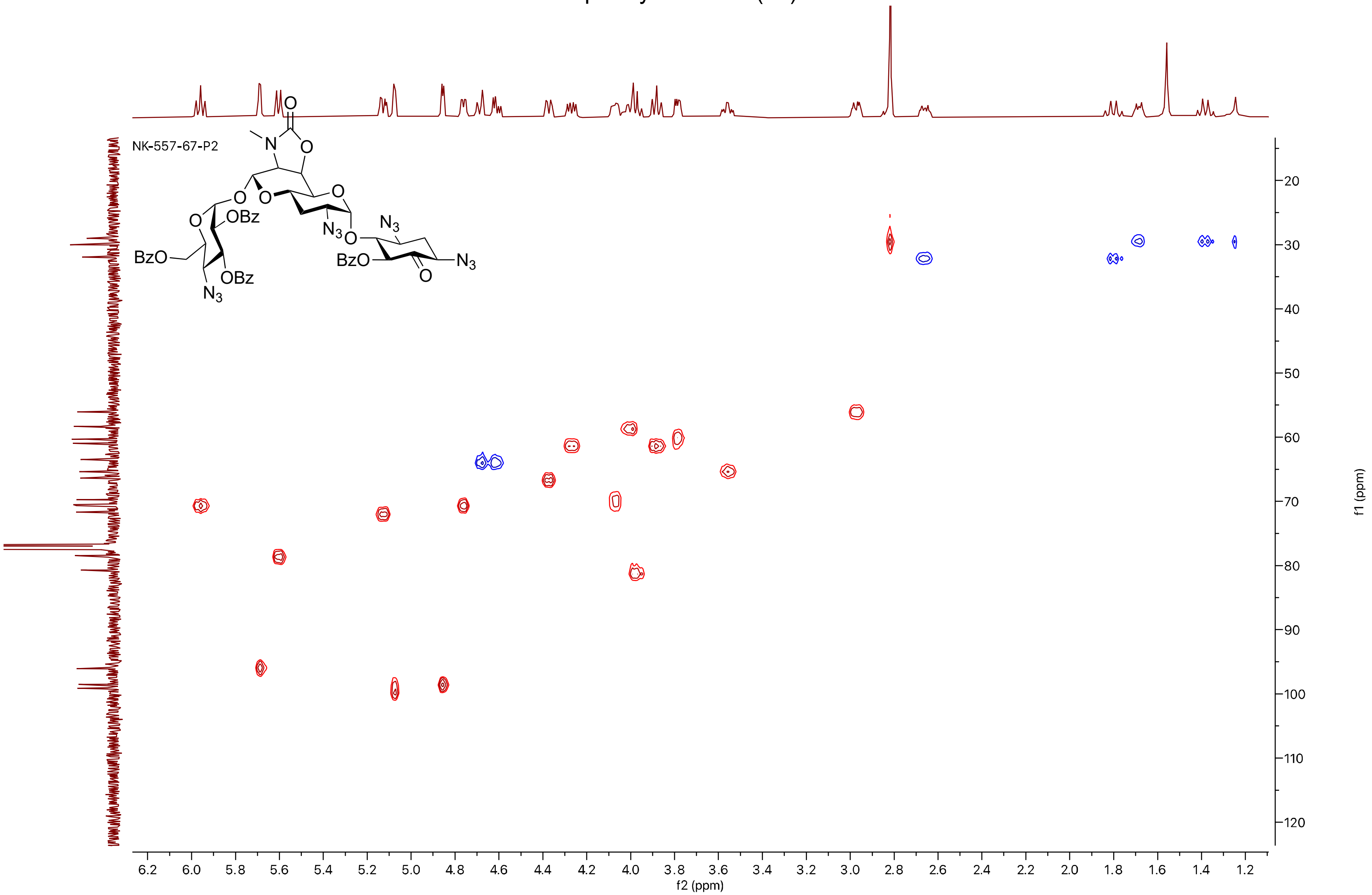

^{1}H NMR Spectrum (500 MHz, $CDCl_3$) of 2,4′-Di-azido-2′,3′,6′-tri-*O*-benzoyl-2,4′-di-(desamino)-6′,7′-oxazolidino-α,β-aprabiosamine (**28**)

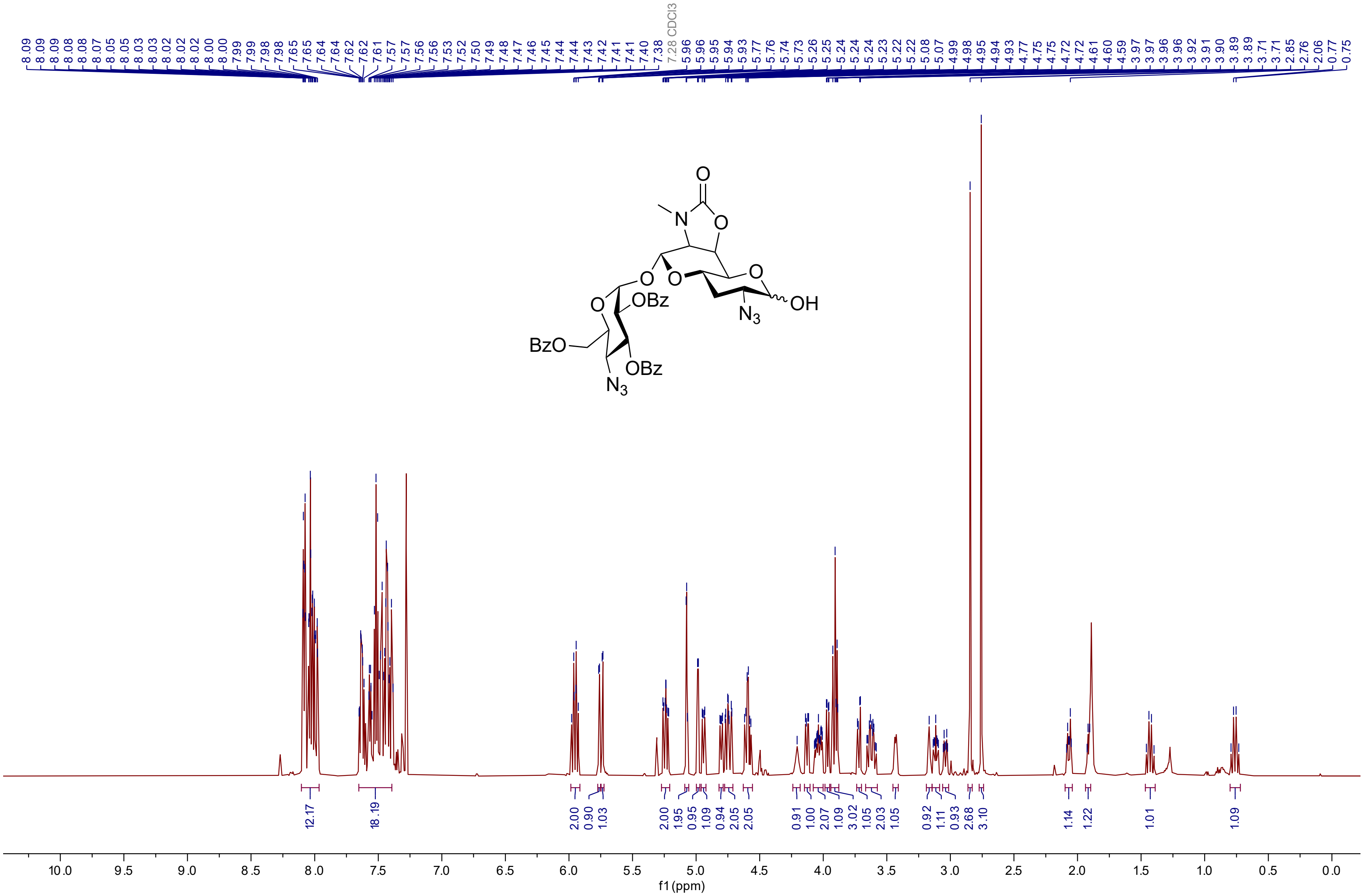

1D Selective gradient TOCSY (600 MHz, $CDCl_3$) of 2,4′-Di-azido-2′,3′,6′-tri-*O*-benzoyl-2,4′-di-(desamino)-6′,7′-oxazolidino-α,β-aprabiosamine (**28**)

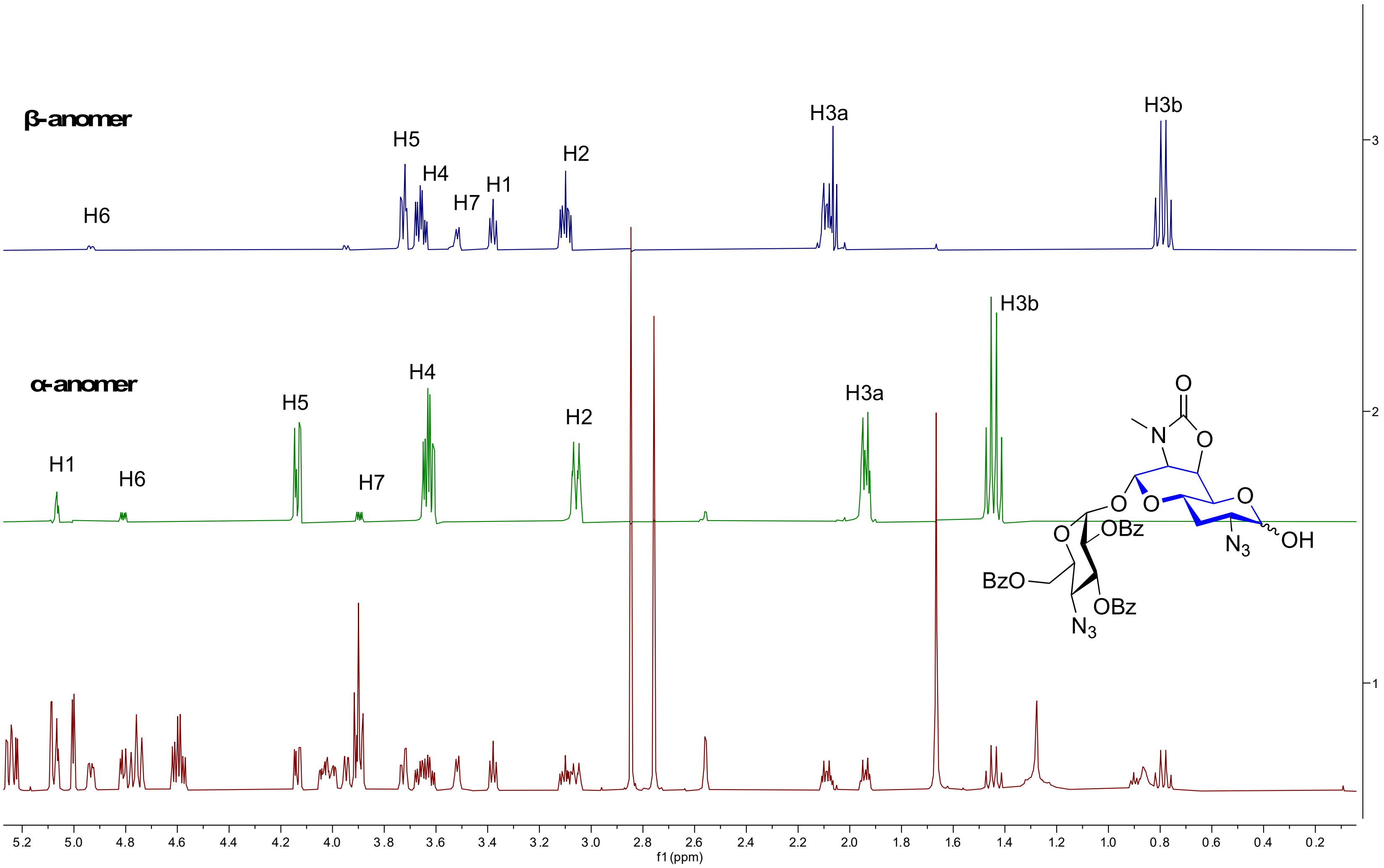

1D Selective gradient TOCSY (600 MHz, $CDCl_3$) of 2,4′-Di-azido-2′,3′,6′-tri-*O*-benzoyl-2,4′-di-(desamino)-6′,7′-oxazolidino-α,β-aprabiosamine (**28**)

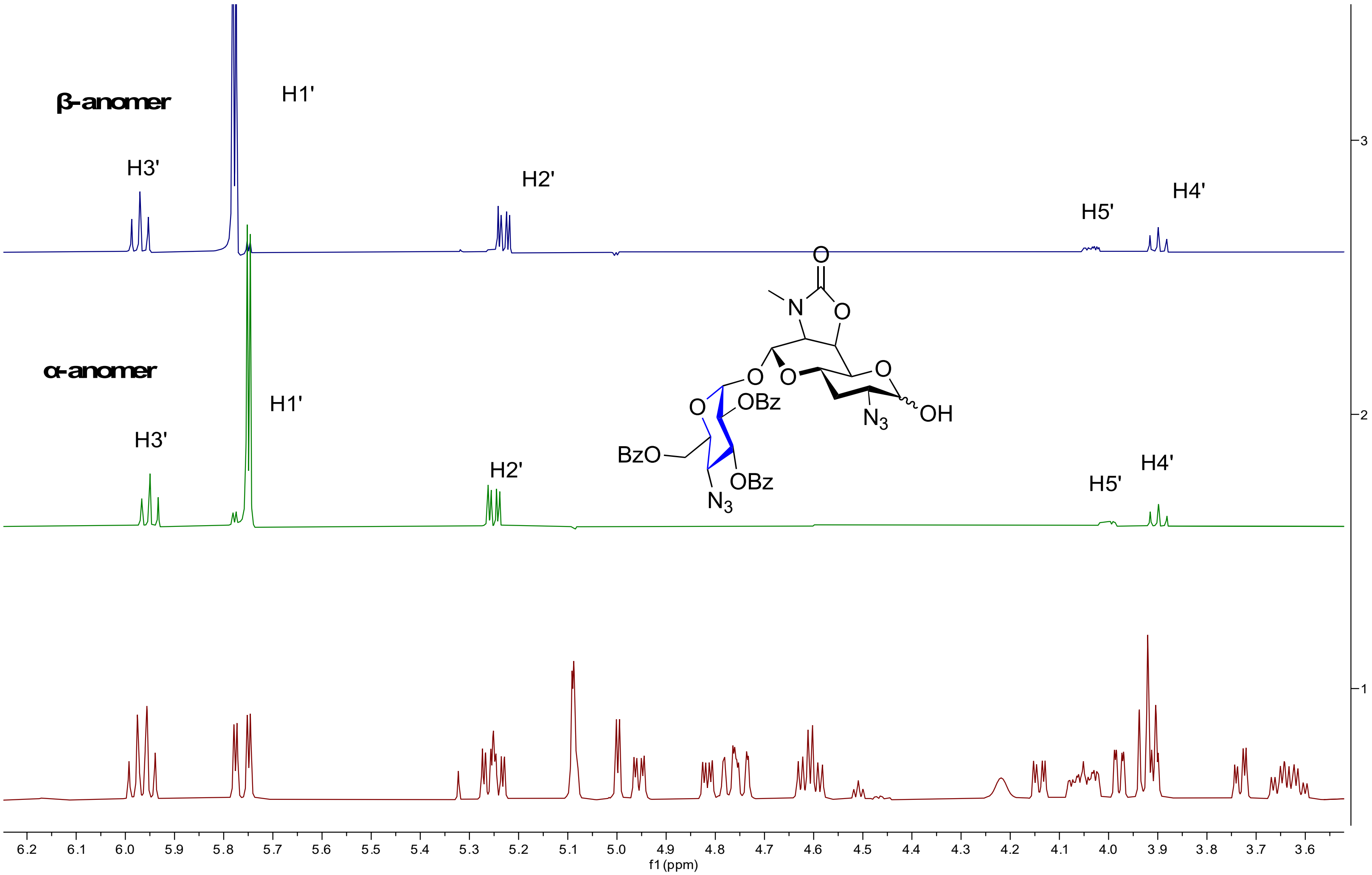

^{13}C NMR Spectrum (125 MHz, $CDCl_3$) of 2,4′-Di-azido-2′,3′,6′-tri-*O*-benzoyl-2,4′-di-(desamino)-6′,7′-oxazolidino-α,β-aprabiosamine (**28**)

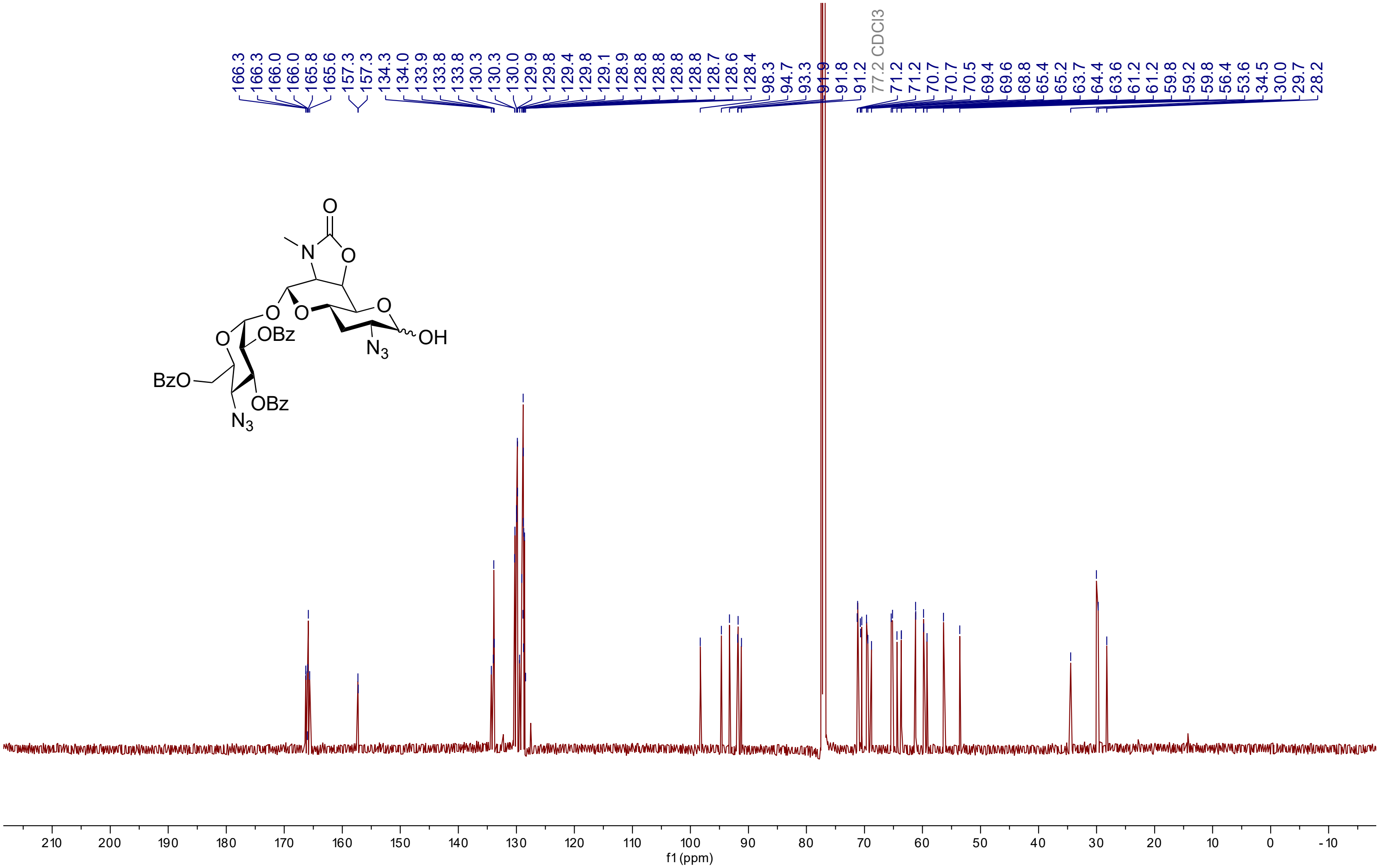

^{1}H-^{1}H COSY Spectrum (500 MHz, CDCl$_3$) of 2,4′-Di-azido-2′,3′,6′-tri-*O*-benzoyl-2,4′-di-(desamino)-6′,7′-oxazolidino-α,β-aprabiosamine (**28**)

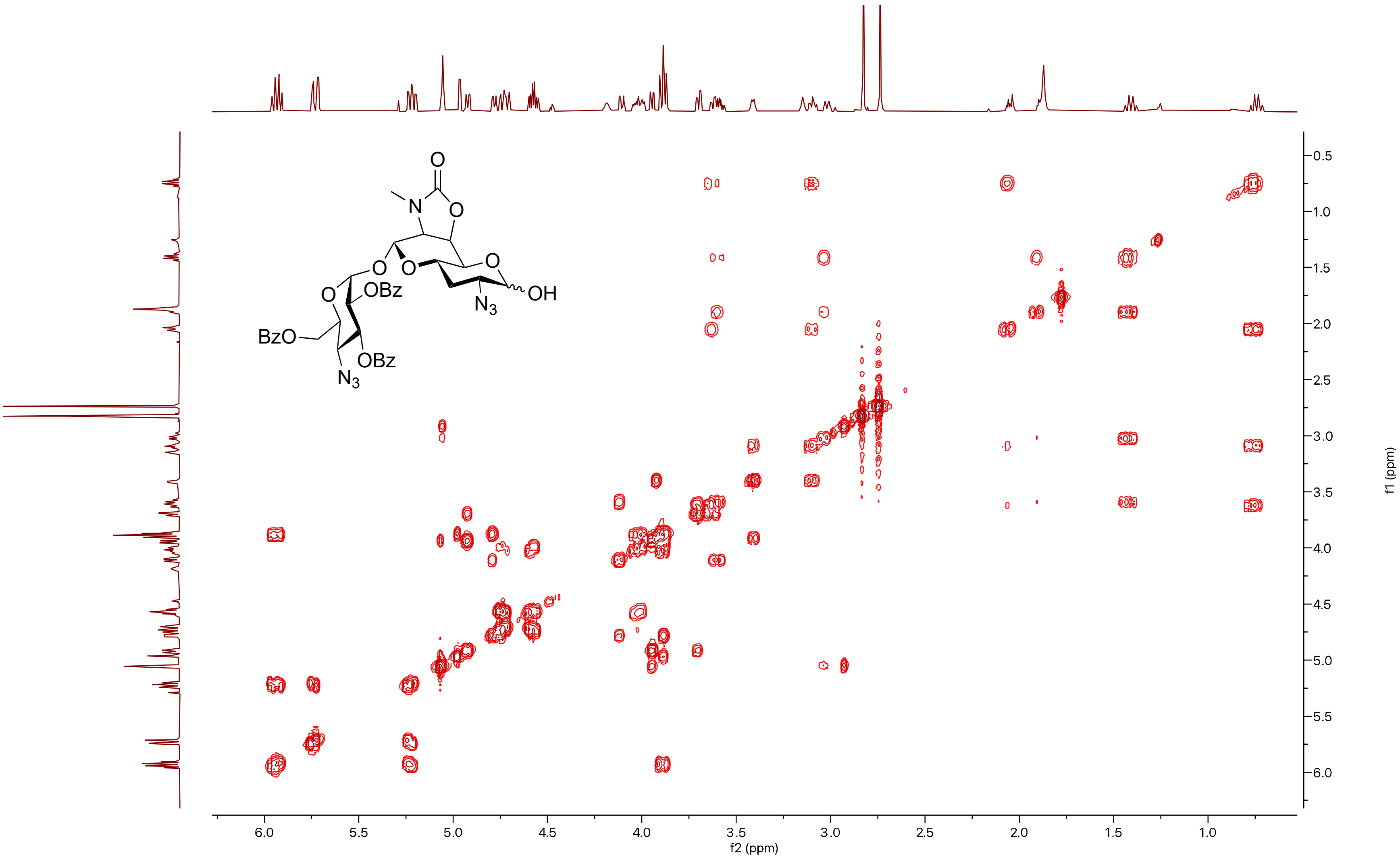

HSQC Spectrum (500 MHz, $CDCl_3$) of 2,4′-Di-azido-2′,3′,6′-tri-*O*-benzoyl-2,4′-di-(desamino)-6′,7′-oxazolidino-α,β-aprabiosamine (**28**)

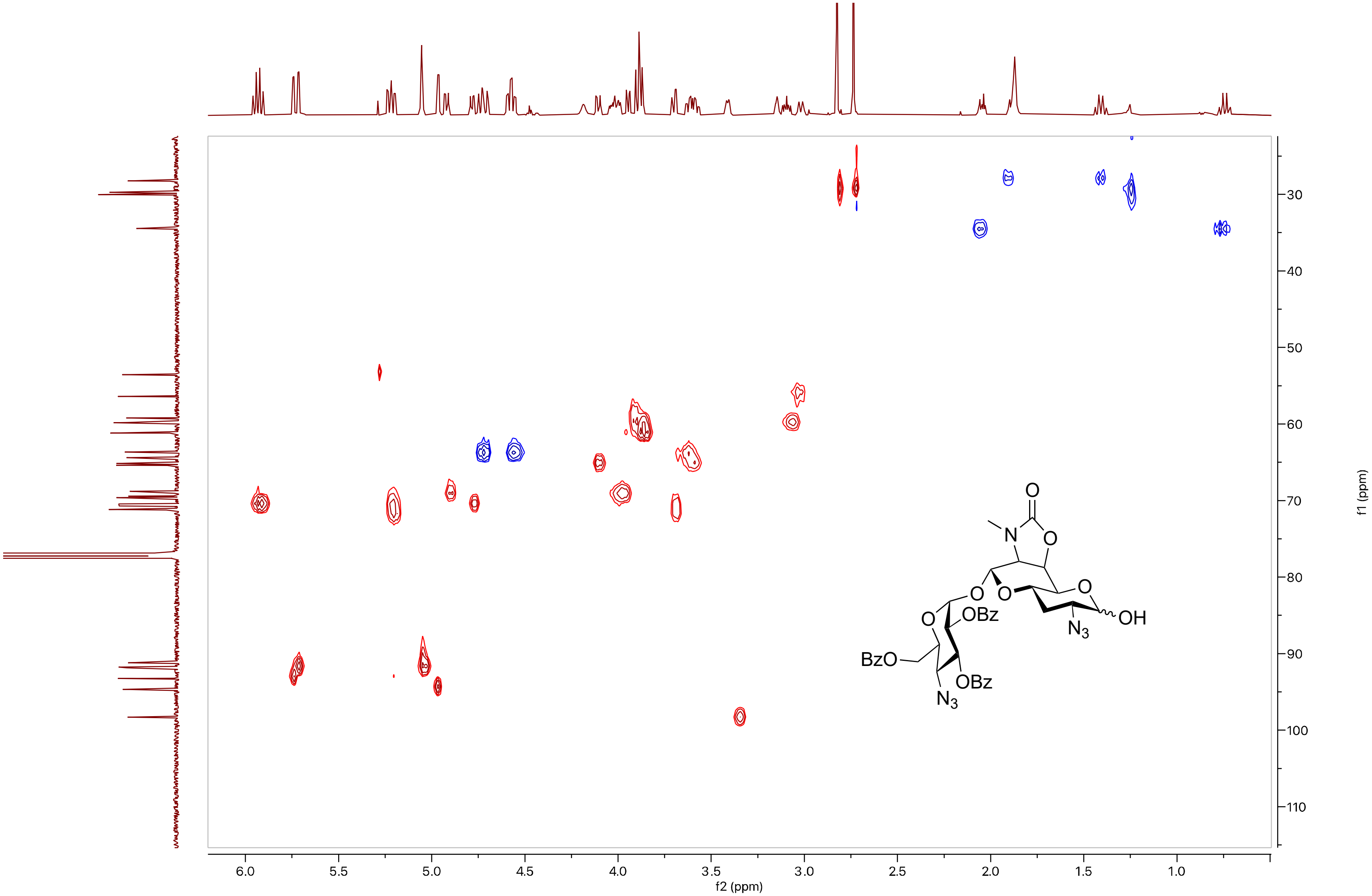

^{1}H NMR Spectrum (500 MHz, $CDCl_3$) of 2,3-Dibenzoyloxyphenyl 2',4''-di-azido-6',2'',3'',6''-tetra-*O*-benzoyl-2',4''-di-(desamino)-6',7'-oxazolidino-aprabiosaminide (**30**)

NK-570-125

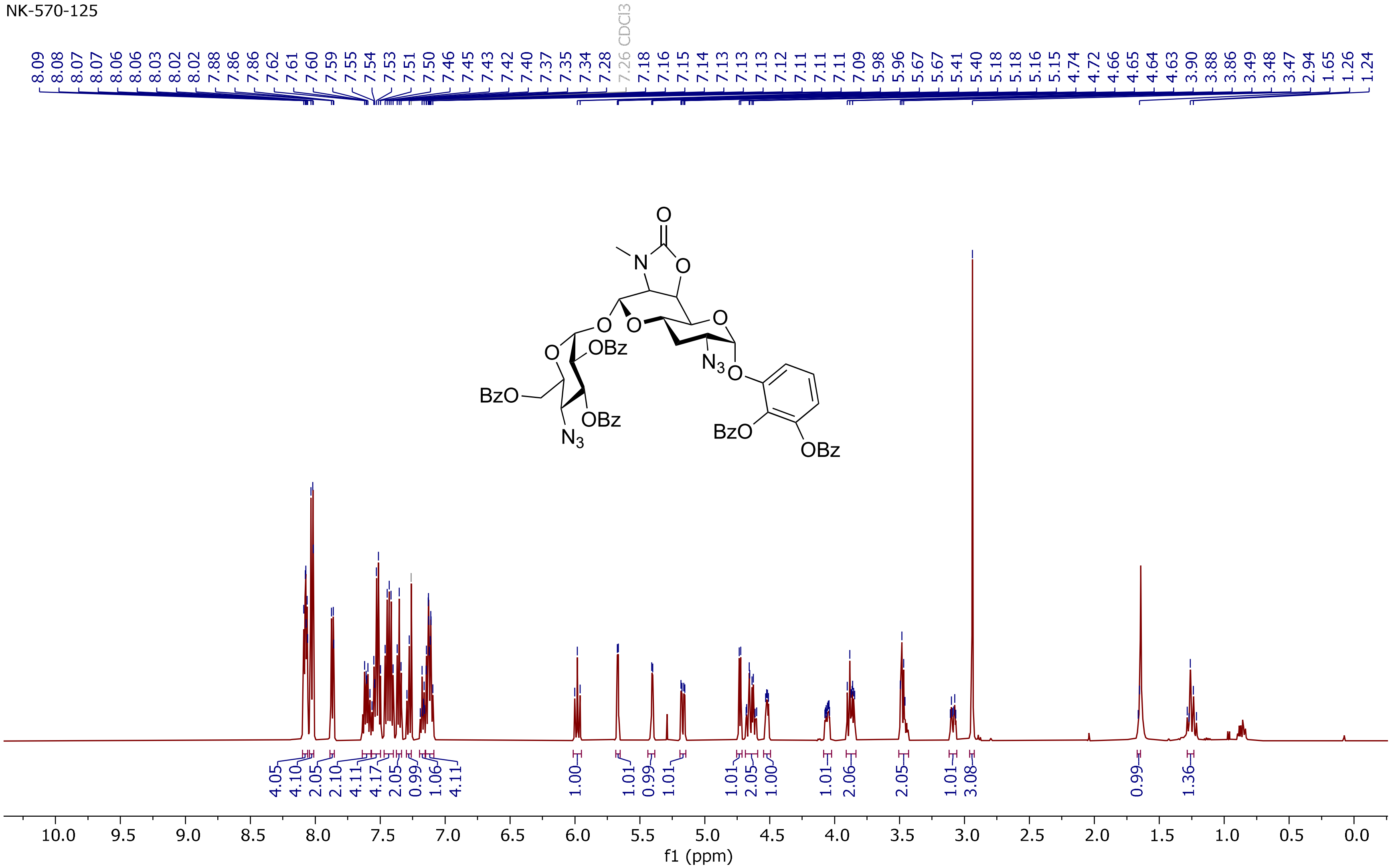

^{13}C NMR Spectrum (125 MHz, $CDCl_3$) of 2,3-Dibenzoyloxyphenyl 2',4''-di-azido-6',2'', 3'',6''-tetra-*O*-benzoyl-2',4''-di-(desamino)-6',7'-oxazolidino-aprabiosaminide (**30**)

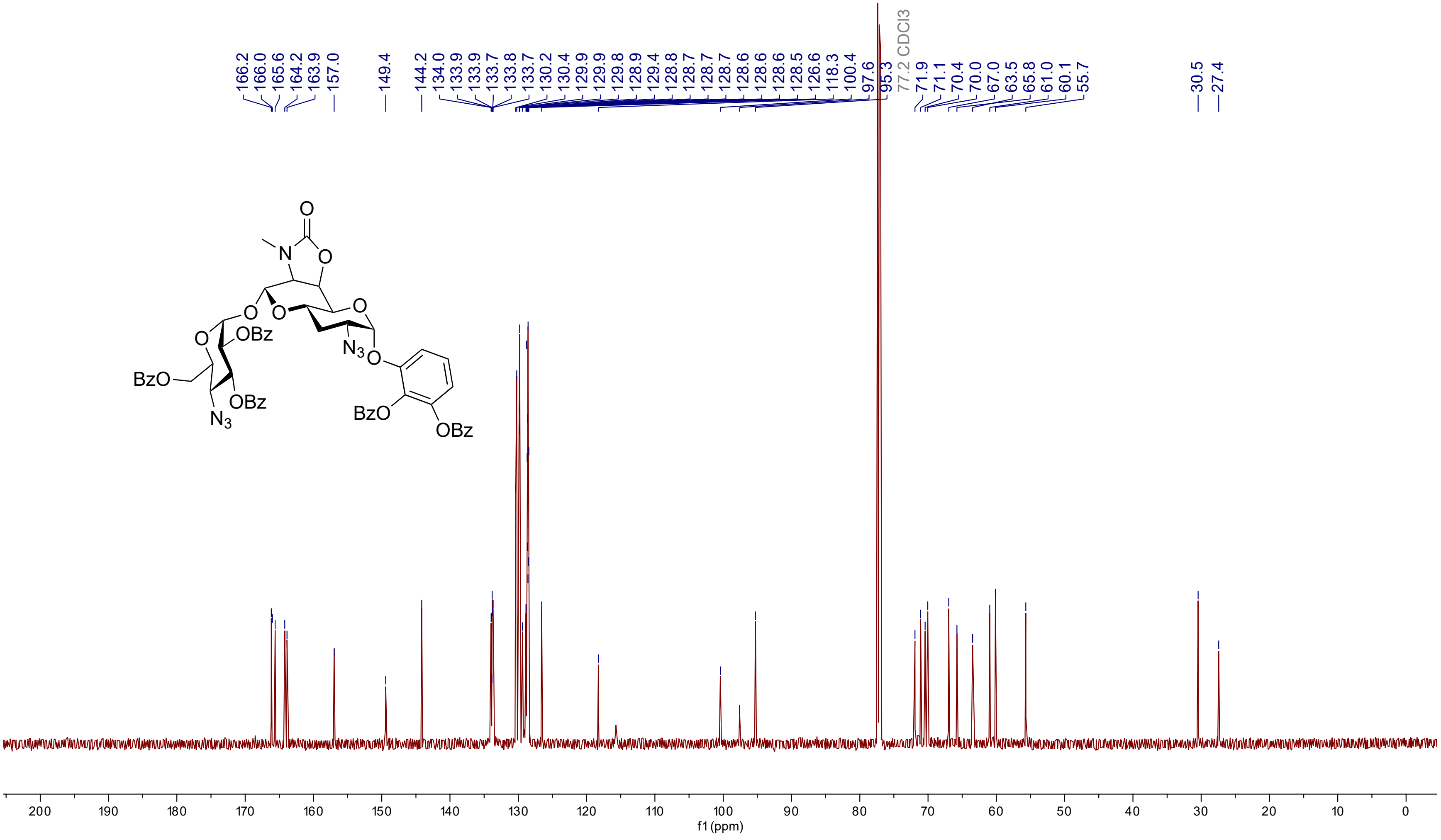

^{1}H-^{1}H COSY Spectrum (500 MHz, $CDCl_3$) of 2,3-Dibenzoyloxyphenyl 2’,4’’-di-azido-6’,2’’, 3’’,6’’-tetra-*O*-benzoyl-2’,4’’-di-(desamino)-6’,7’-oxazolidino-aprabiosaminide (**30**)

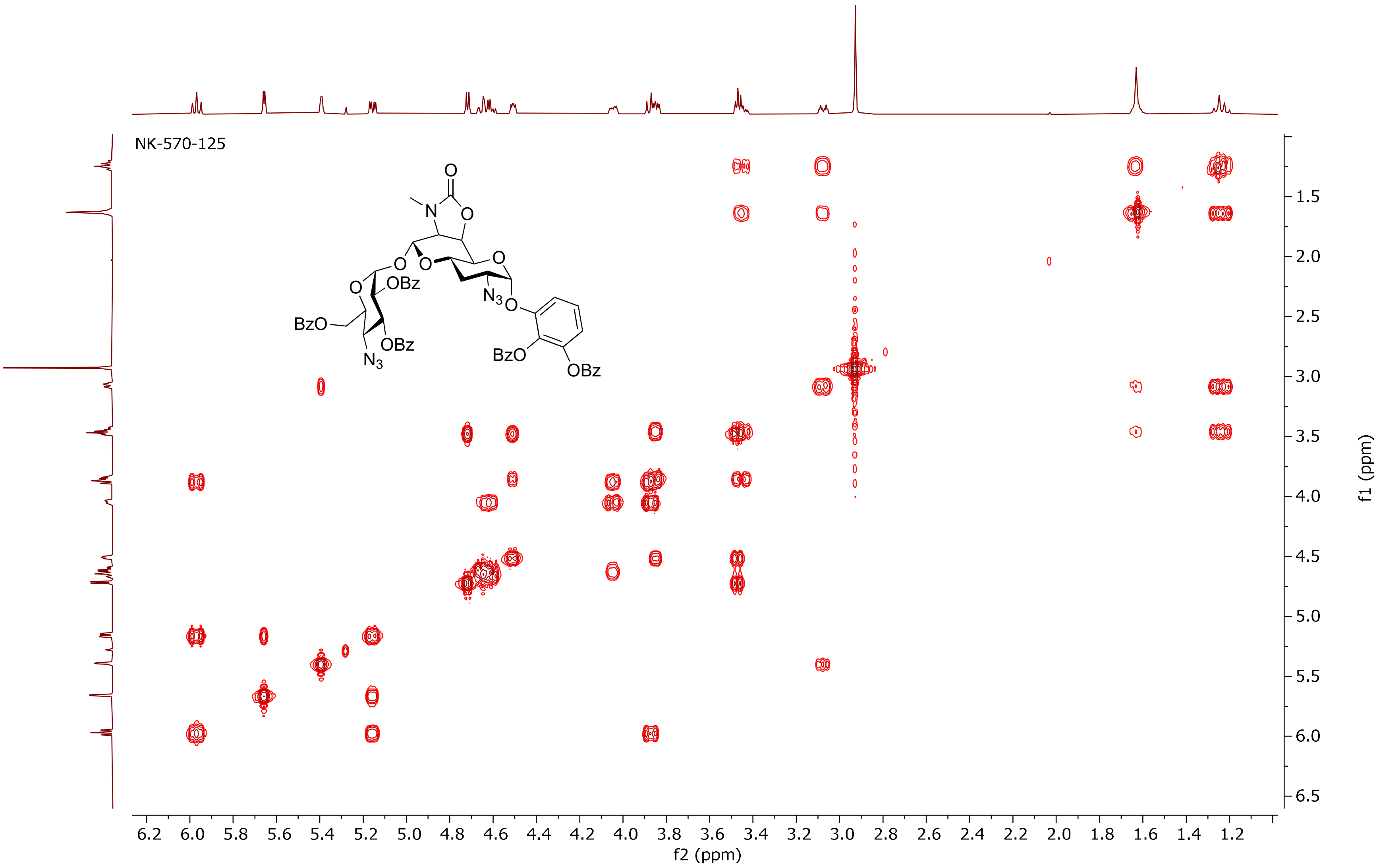

HSQC Spectrum (500 MHz, $CDCl_3$) of 2,3-Dibenzoyloxyphenyl 2’,4’’-di-azido-6’,2’’, 3’’,6’’-tetra-*O*-benzoyl-2’,4’’-di-(desamino)-6’,7’-oxazolidone-aprabiosaminide (**30**)

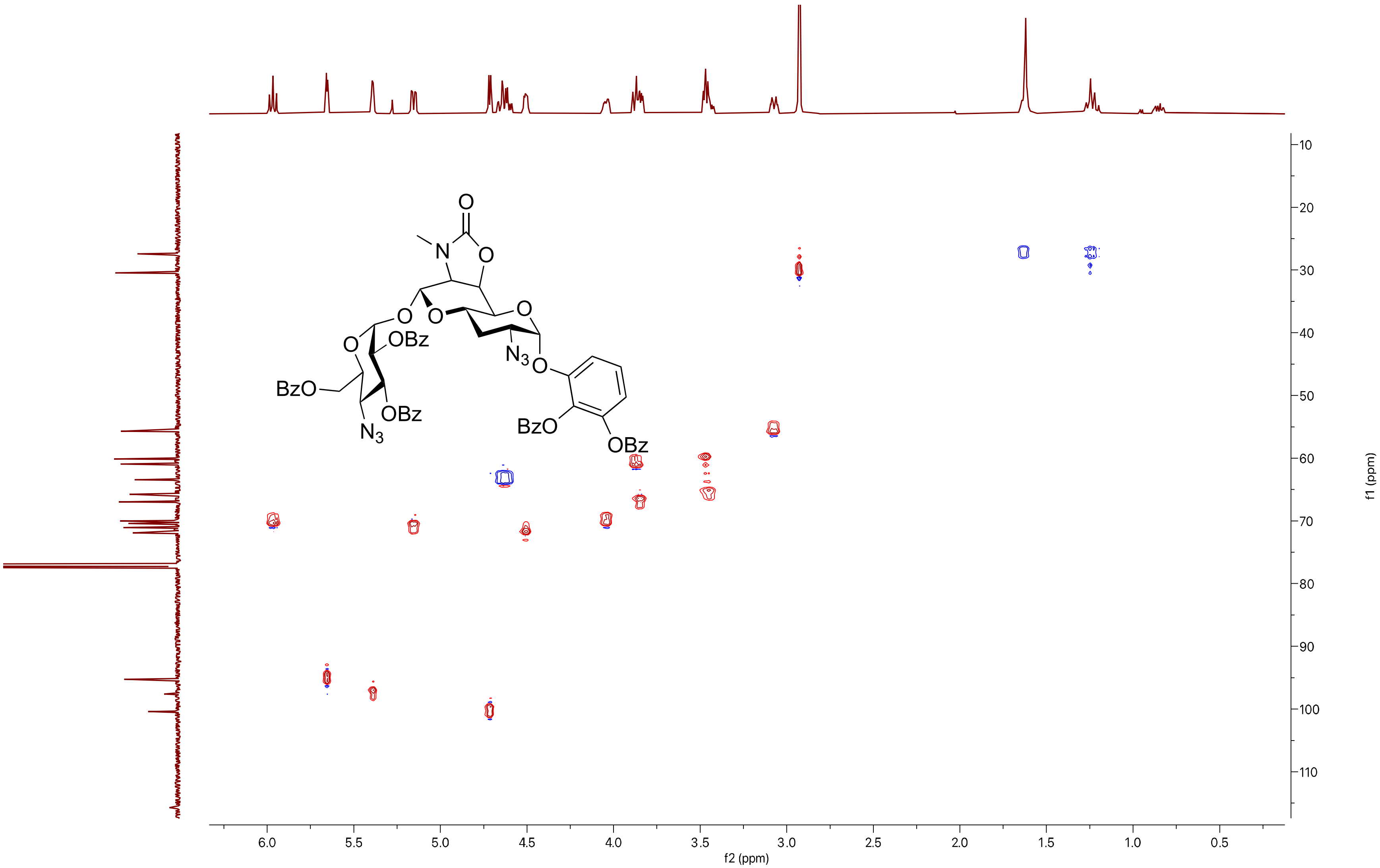

^{1}H NMR Spectrum (600 MHz, $CDCl_3$) of *N*-Phenyltrifluoroacetamidoyl 2,4′-di-azido-2′,3′,6′-tri-*O*-benzoyl-2,4′-di(desamino)-6′,7′-oxazolidino-α,β-aprabiosaminide (**31**)

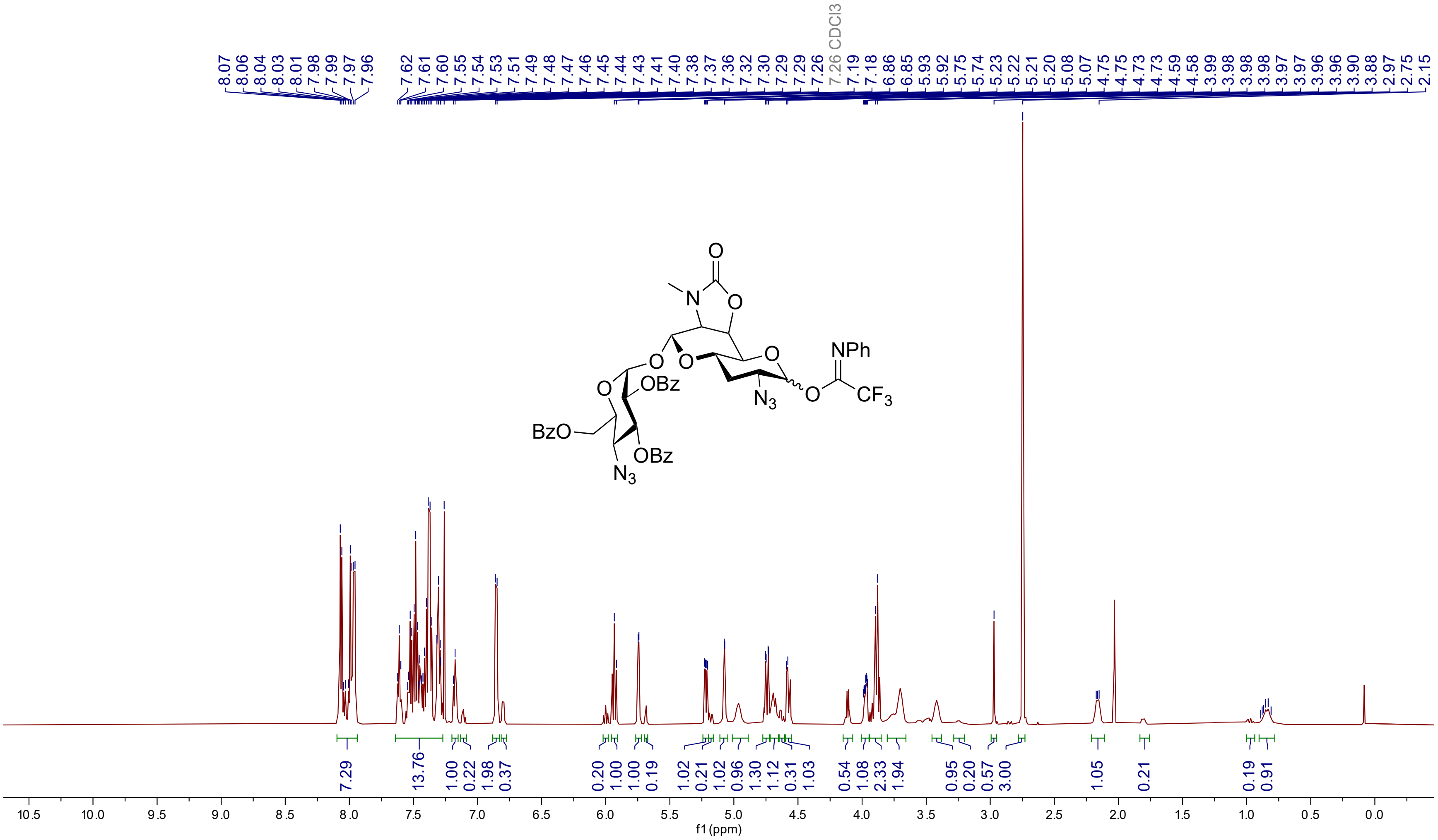

1D Selective gradient TOCSY (600 MHz, $CDCl_3$) of *N*-Phenyltrifluoroacetamidoyl 2,4′-di-azido-2′,3′,6′-tri-*O*-benzoyl-2,4′-di(desamino)-6′,7′-oxazolidino-α,β-aprabiosaminide (**31**)

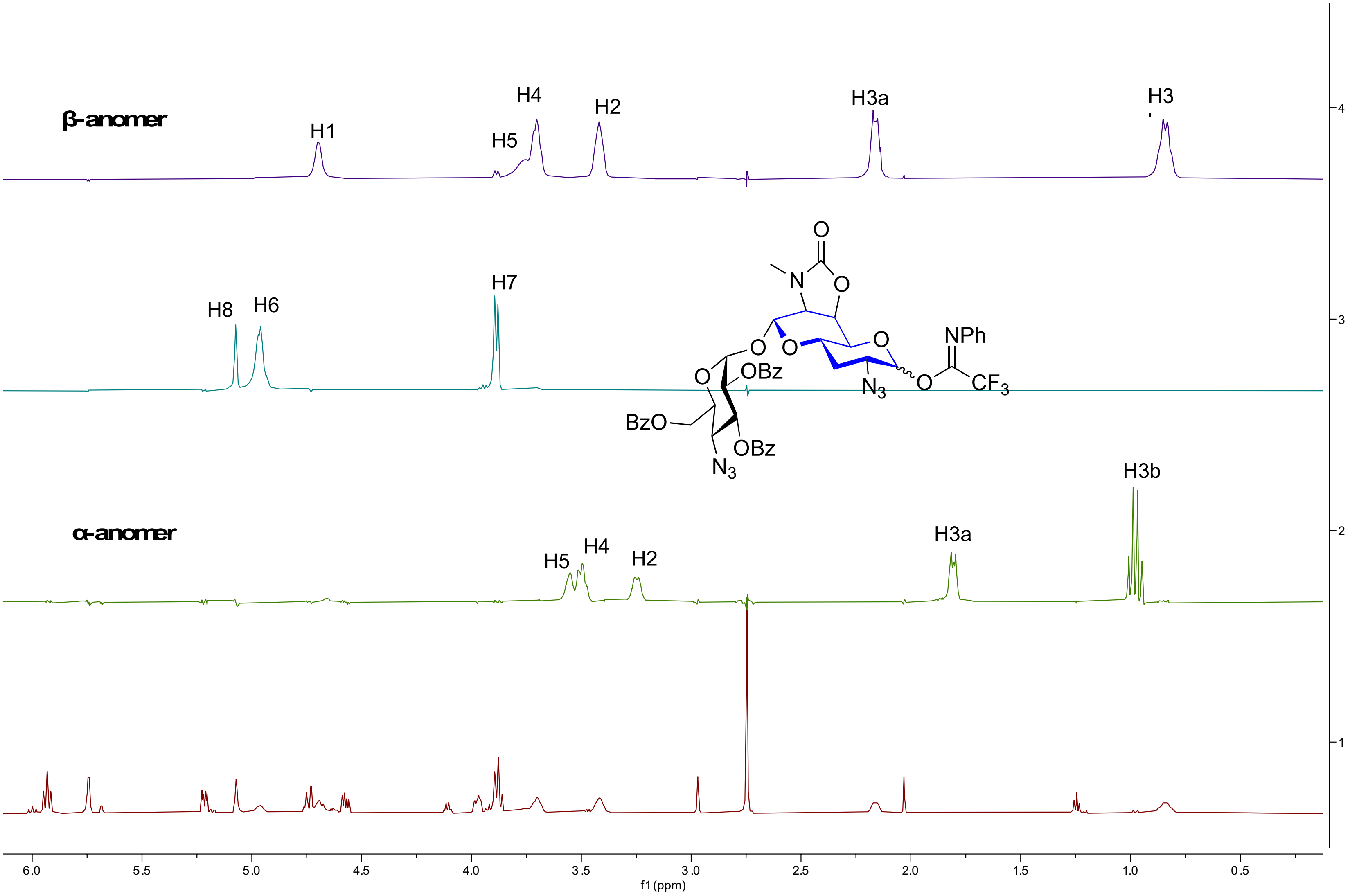

1D Selective gradient TOCSY (600 MHz, $CDCl_3$) of *N*-Phenyltrifluoroacetamidoyl 2,4′-di-azido-2′,3′,6′-tri-*O*-benzoyl-2,4′-di(desamino)-6′,7′-oxazolidino-α,β-aprabiosaminide (**31**)

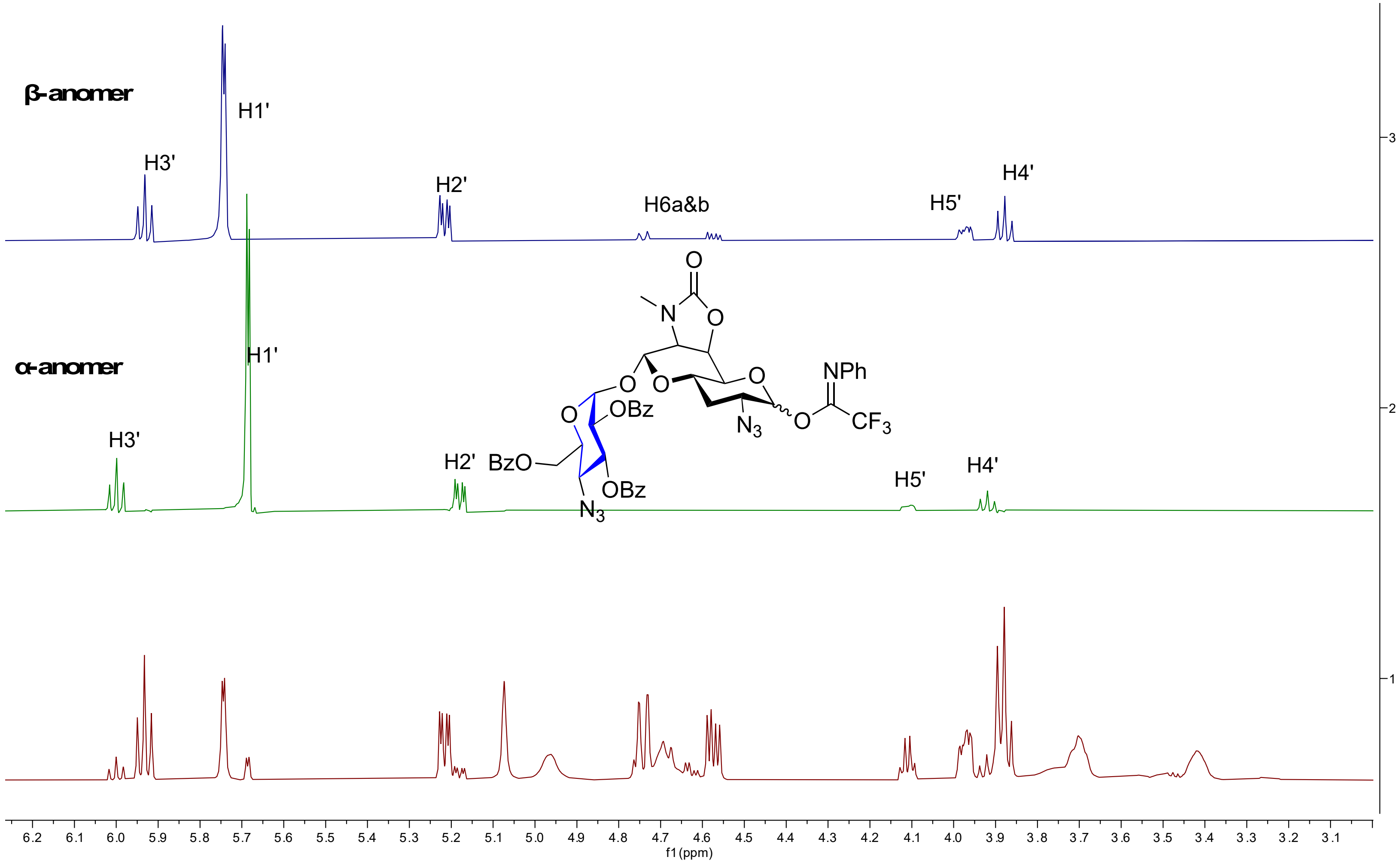

^{13}C NMR Spectrum (200 MHz, $CDCl_3$) of *N*-Phenyltrifluoroacetamidoyl 2,4′-di-azido-2′,3′,6′-tri-*O*-benzoyl-2,4′-di(desamino)-6′,7′-oxazolidino-α,β-aprabiosaminide (**31**)

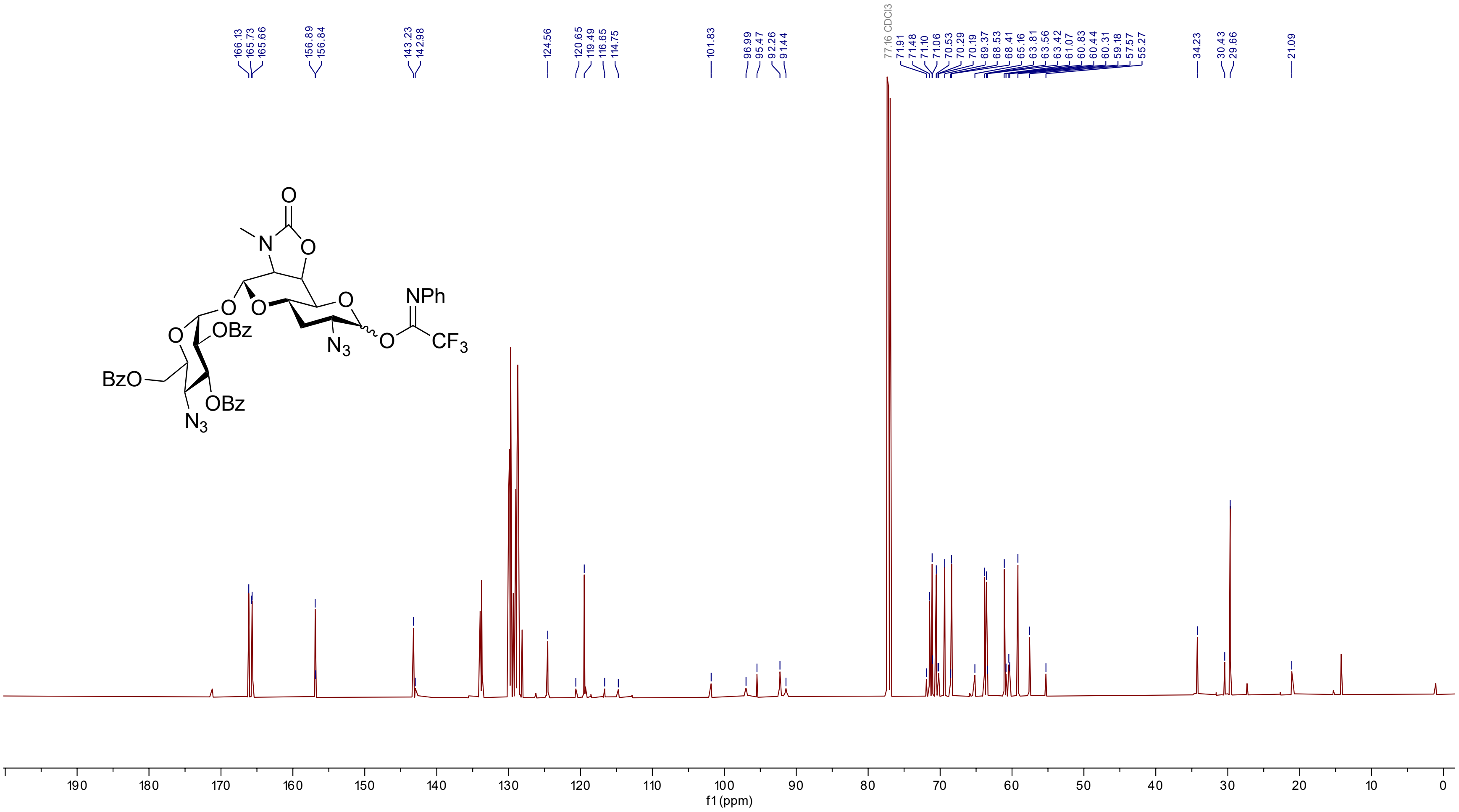

^{1}H-^{1}H COSY Spectrum (600 MHz, $CDCl_3$) of *N*-Phenyltrifluoroacetamidoyl 2,4′-di-azido-2′,3′,6′-tri-*O*-benzoyl-2,4′-di(desamino)-6′,7′-oxazolidino-α,β-aprabiosaminide (**31**)

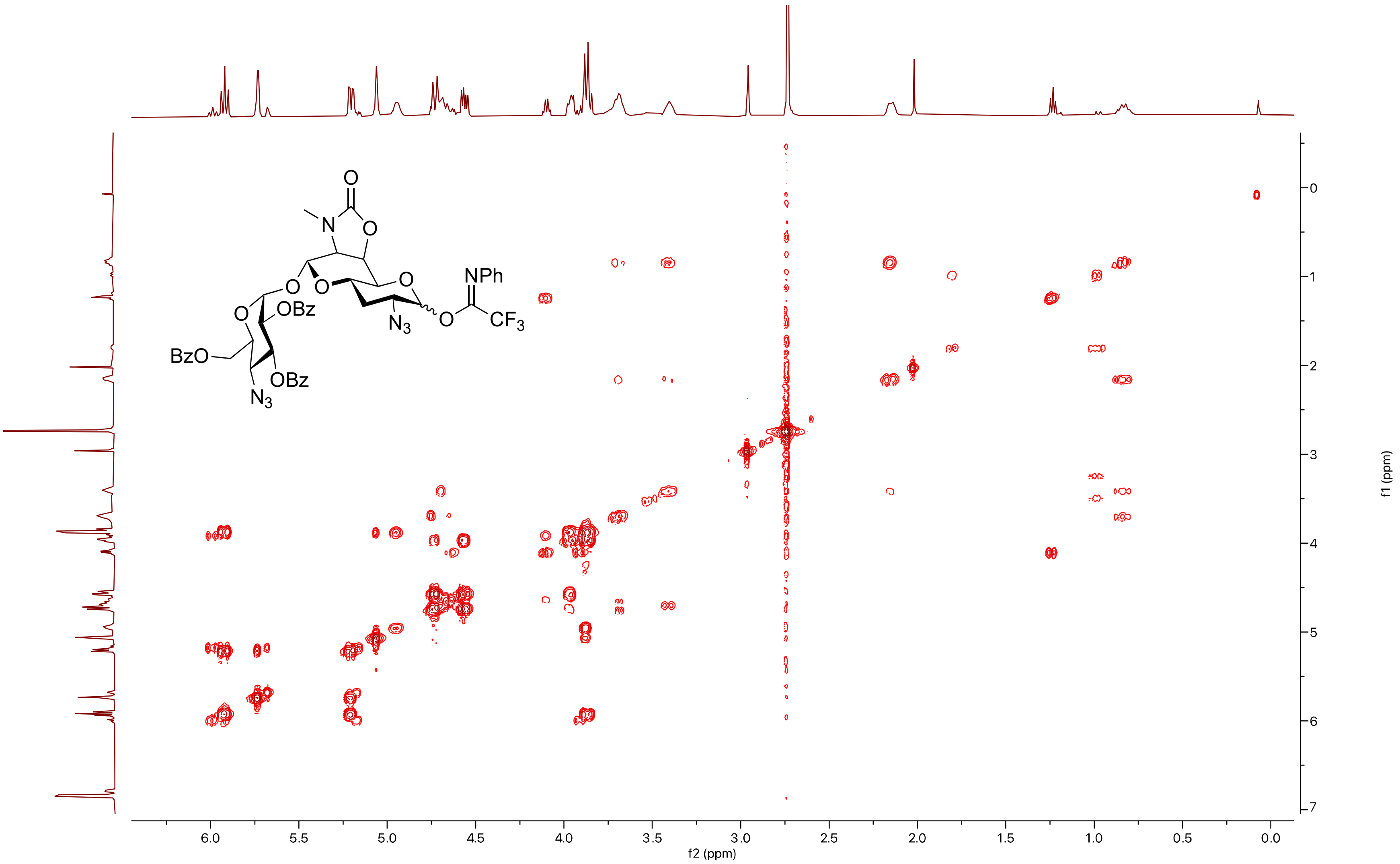

HSQC Spectrum (600 MHz, $CDCl_3$) of *N*-Phenyltrifluoroacetamidoyl 2,4′-di-azido-2′,3′,6′-tri-*O*-benzoyl-2,4′-di(desamino)-6′,7′-oxazolidino-α,β-aprabiosaminide (**31**)

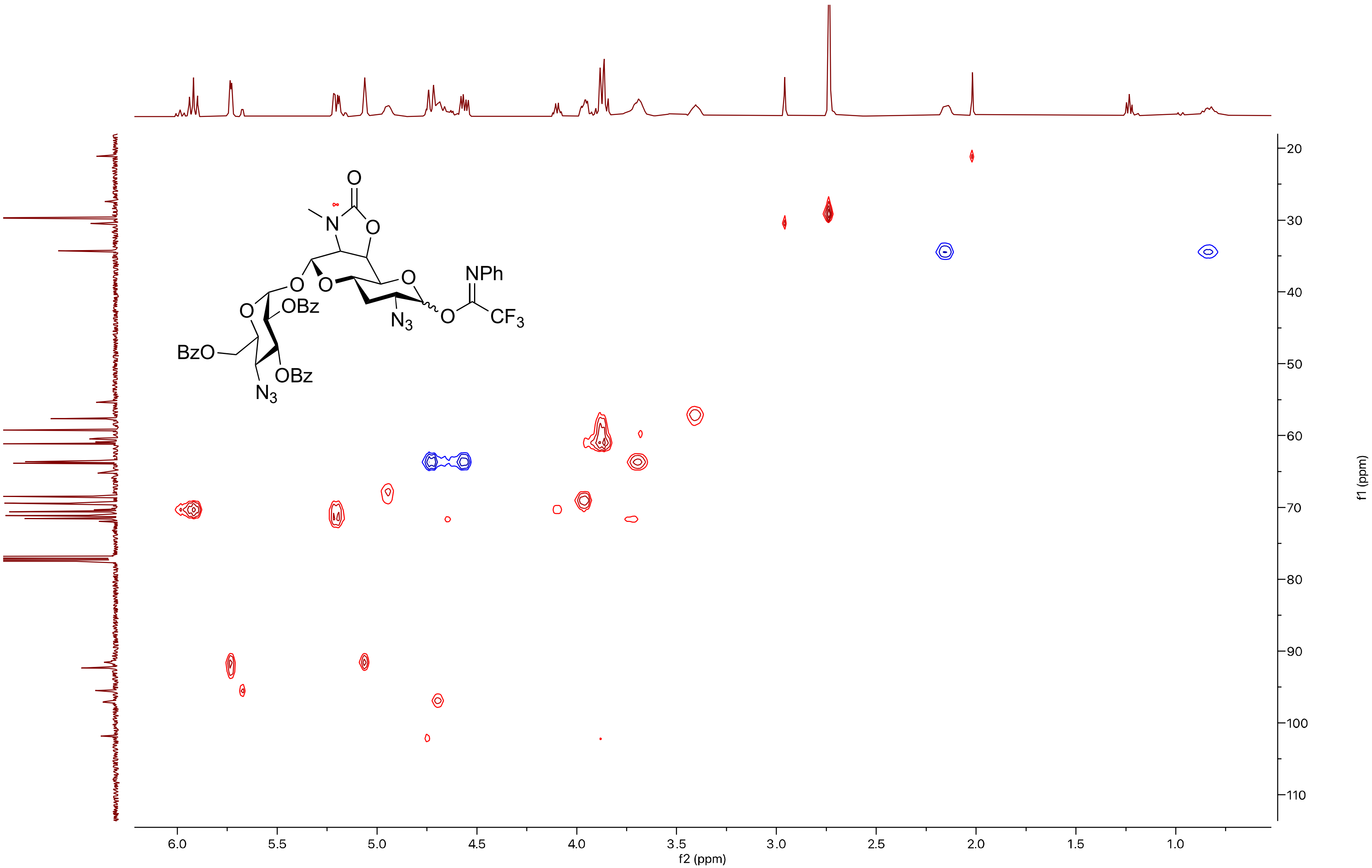

^{1}H NMR Spectrum (600 MHz, $CDCl_3$) of 1,3,2′,4″-Tetra-azido-2,5,6-tri-*O*-benzyl-1,3,2′,4″-tetra-(desamino)-6′,7′-oxazolidino-1’-epi-2-hydroxy-apramycin (**33**)

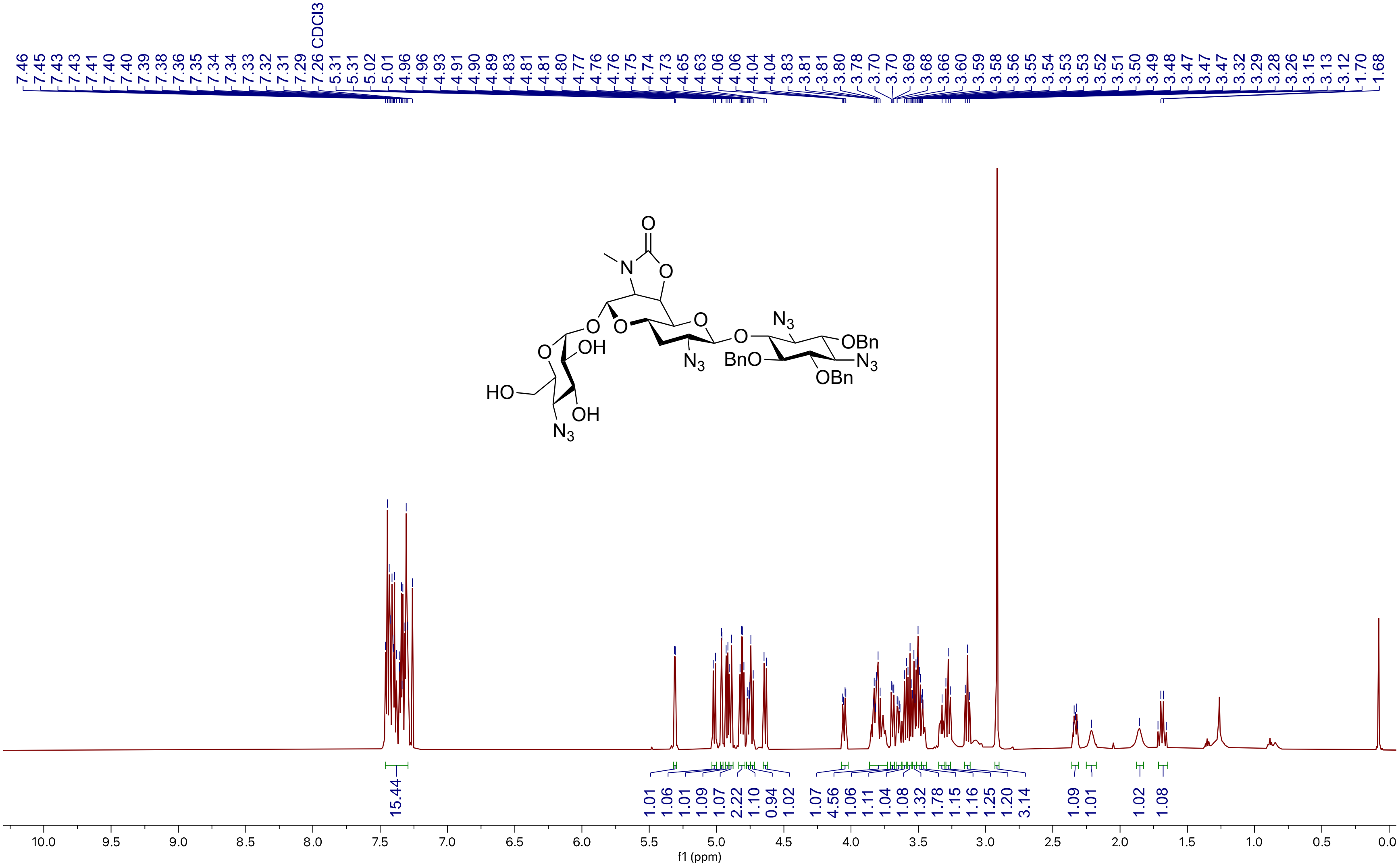

^{13}C NMR Spectrum (150 MHz, $CDCl_3$) of 1,3,2′,4″-Tetra-azido-2,5,6-tri-*O*-benzyl-1,3,2′,4″-tetra-(desamino)-6′,7′-oxazolidino-1’-epi-2-hydroxy-apramycin (**33**)

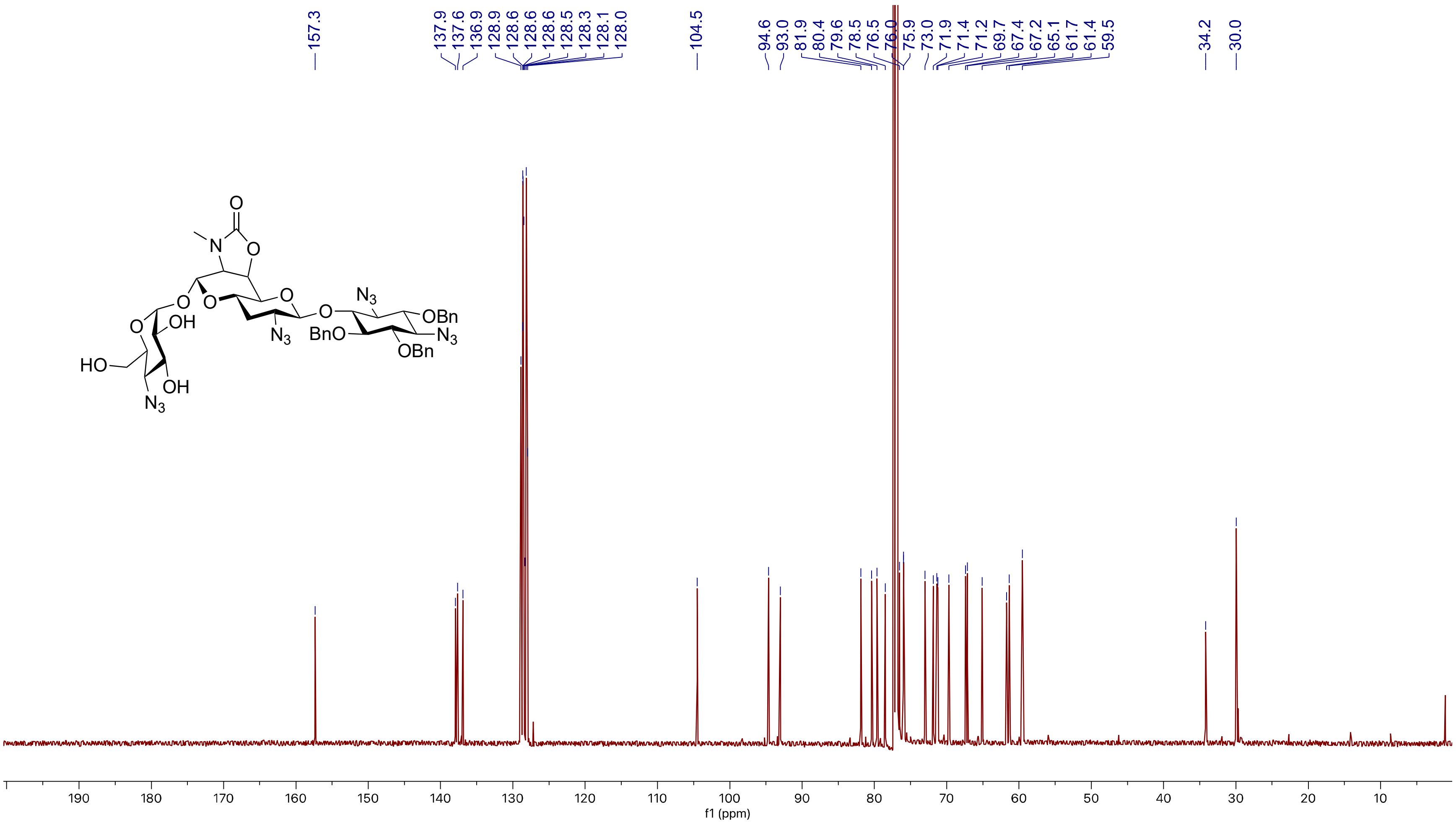

^{1}H-^{1}H COSY Spectrum (600 MHz, $CDCl_3$) of 1,3,2′,4″-Tetra-azido-2,5,6-tri-*O*-benzyl-1,3,2′,4″-tetra-(desamino)-6′,7′-oxazolidino-1’-epi-2-hydroxy-apramycin (**33**)

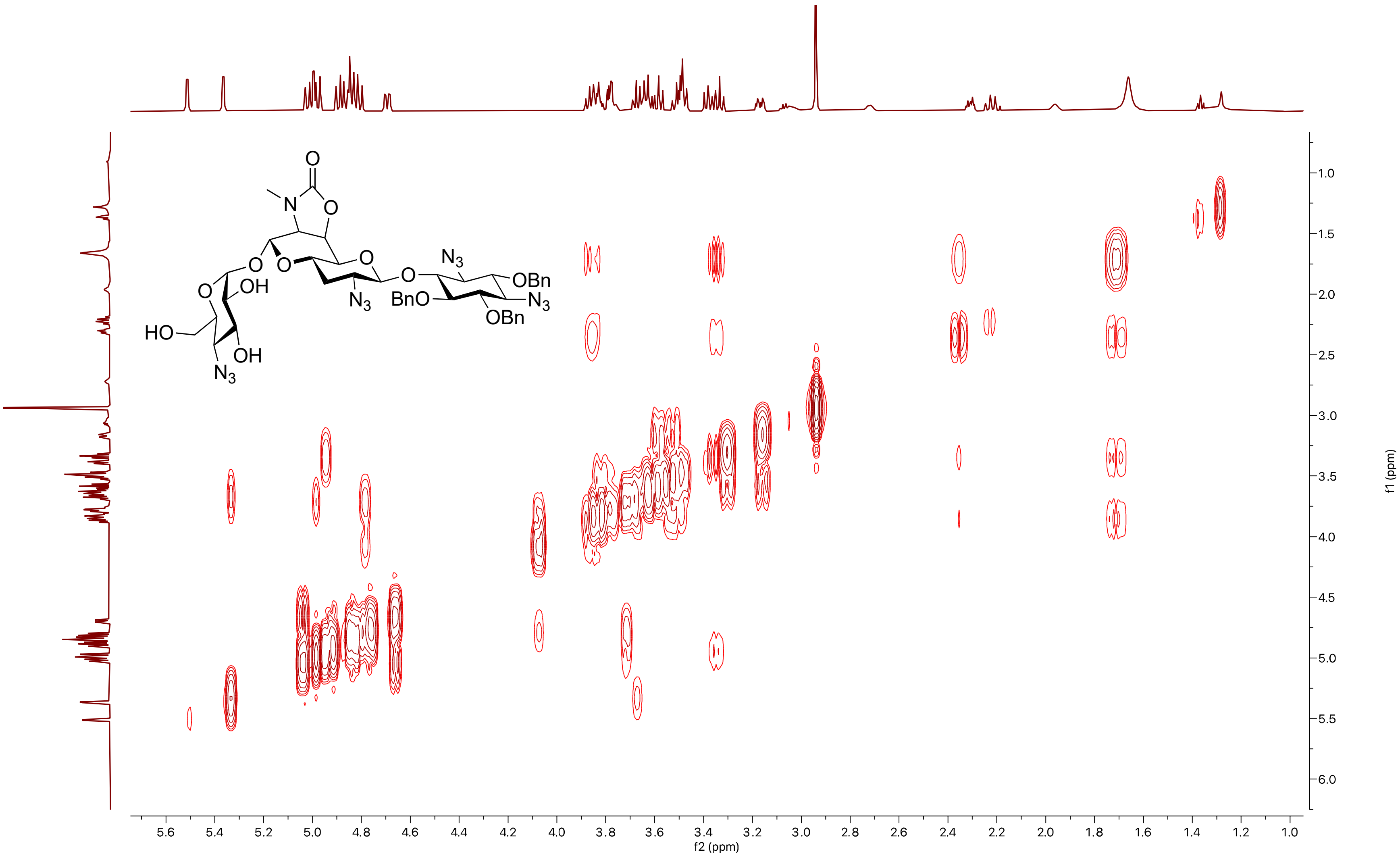

HSQC Spectrum (600 MHz, $CDCl_3$) of 1,3,2′,4″-Tetra-azido-2,5,6-tri-*O*-benzyl-1,3,2′,4″-tetra-(desamino)-6′,7′-oxazolidino-1’-epi-2-hydroxy-apramycin (**33**)

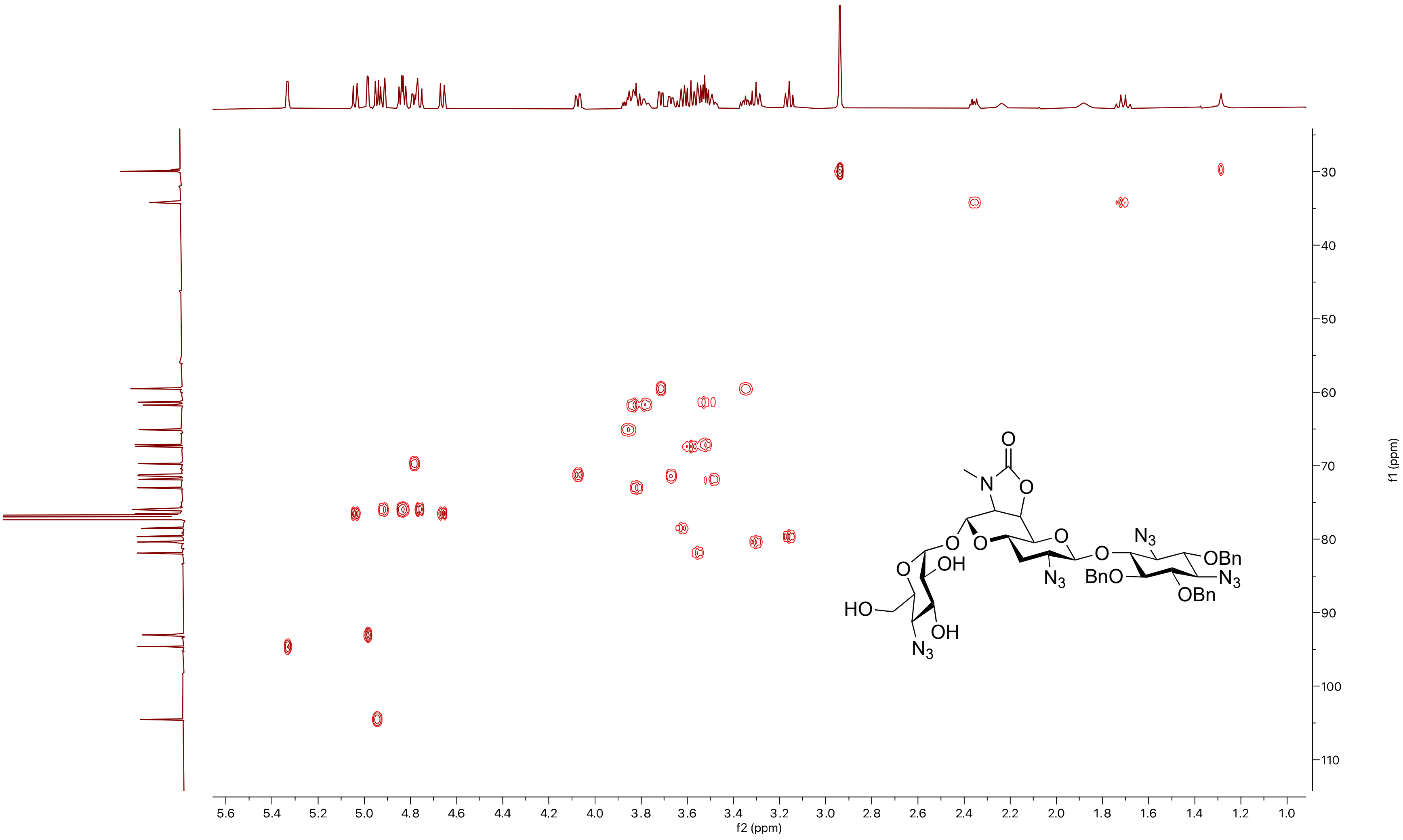

^{1}H NMR Spectrum (600 MHz, $CDCl_3$) of 1,3,2′,4″-Tetra-azido-2,5,6-tri-*O*-benzyl-1,3,2′,4″-tetra-(desamino)-6′,7′-oxazolidino-2-hydroxy-apramycin (**34**)

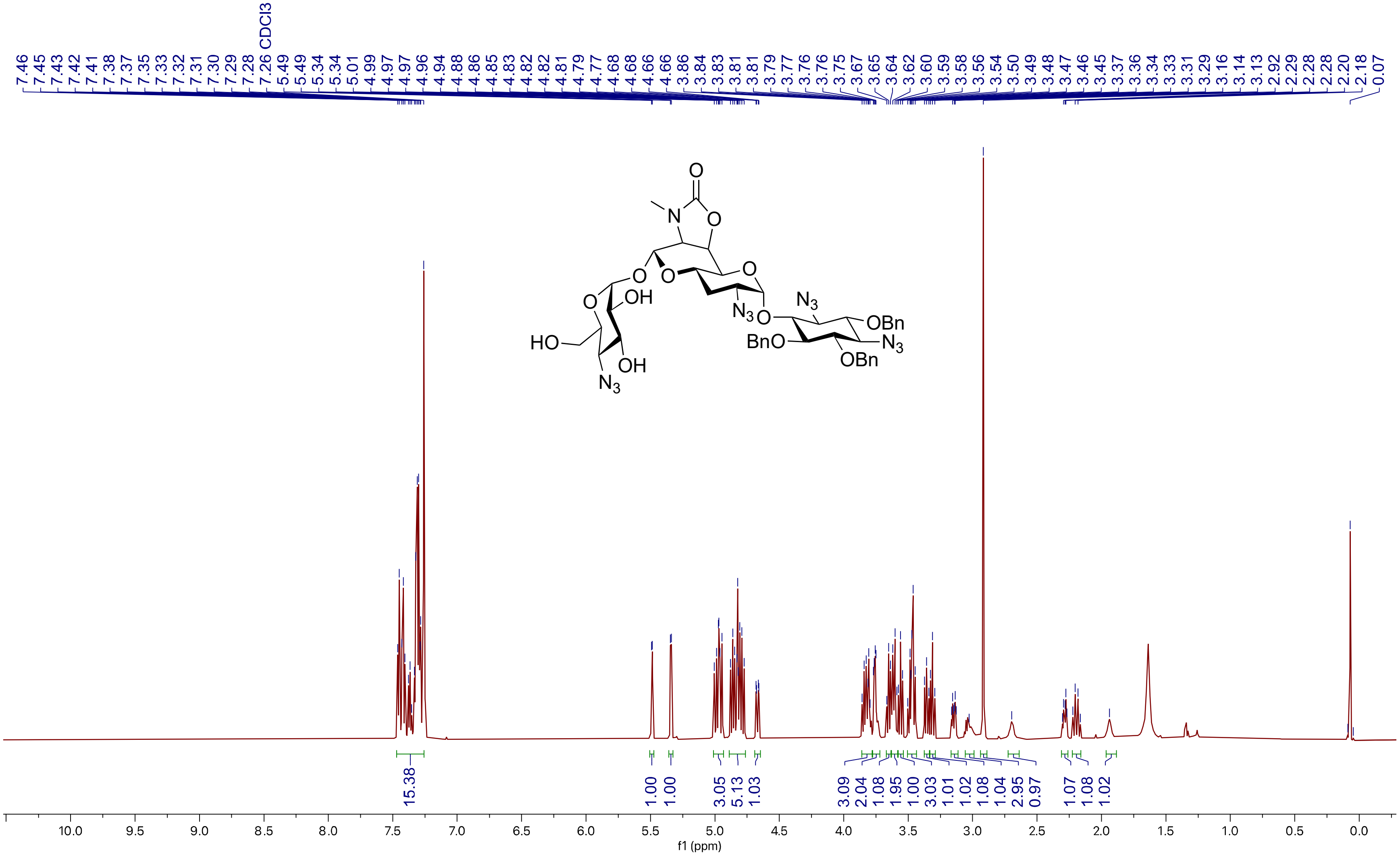

^{13}C NMR Spectrum (150 MHz, $CDCl_3$) of 1,3,2′,4″-Tetra-azido-2,5,6-tri-*O*-benzyl-1,3,2′,4″-tetra-(desamino)-6′,7′-oxazolidino-2-hydroxy-apramycin (**34**)

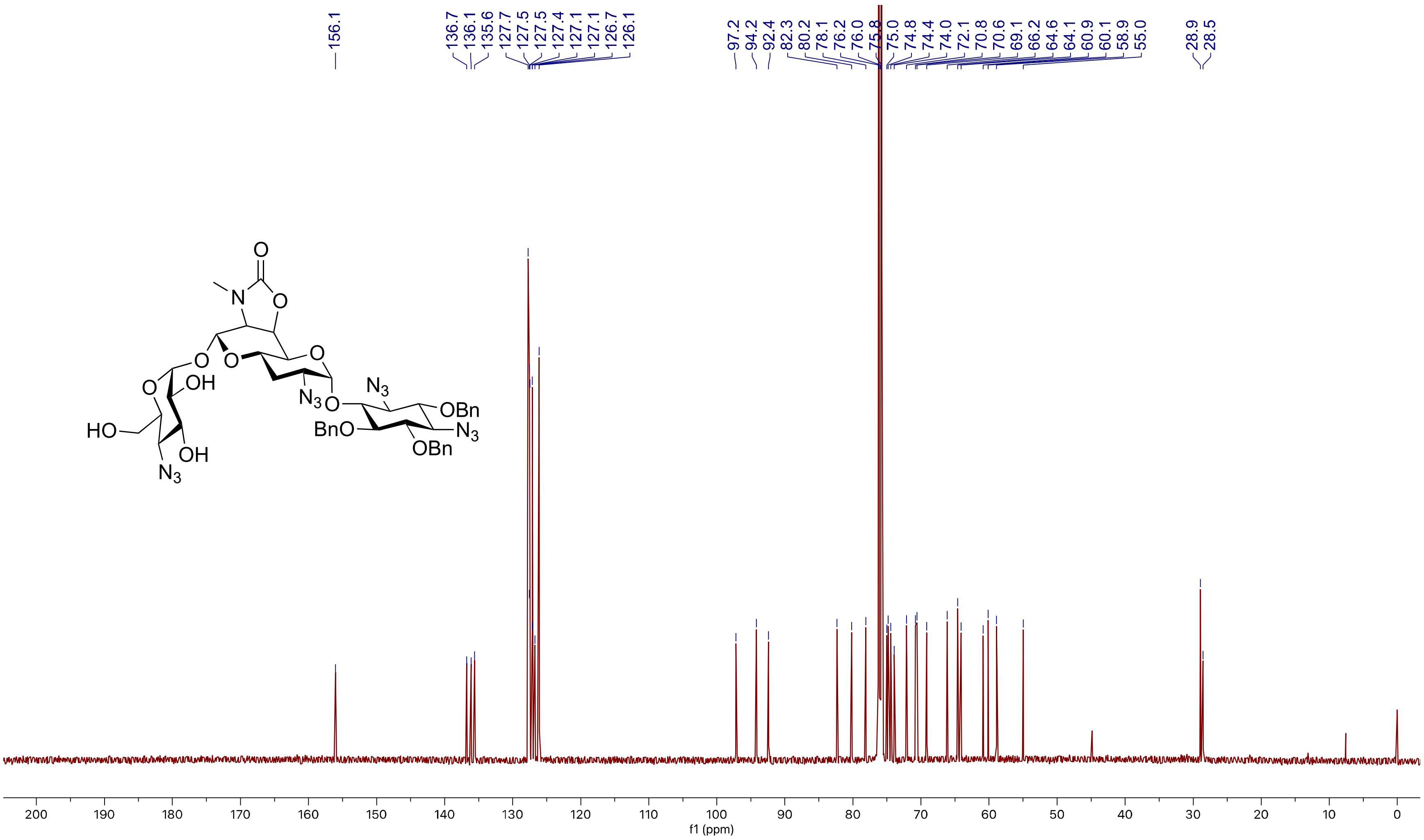

^{1}H-^{1}H COSY Spectrum (600 MHz, $CDCl_3$) of 1,3,2′,4″-Tetra-azido-2,5,6-tri-*O*-benzyl-1,3,2′,4″-tetra-(desamino)-6′,7′-oxazolidino-2-hydroxy-apramycin (**34**)

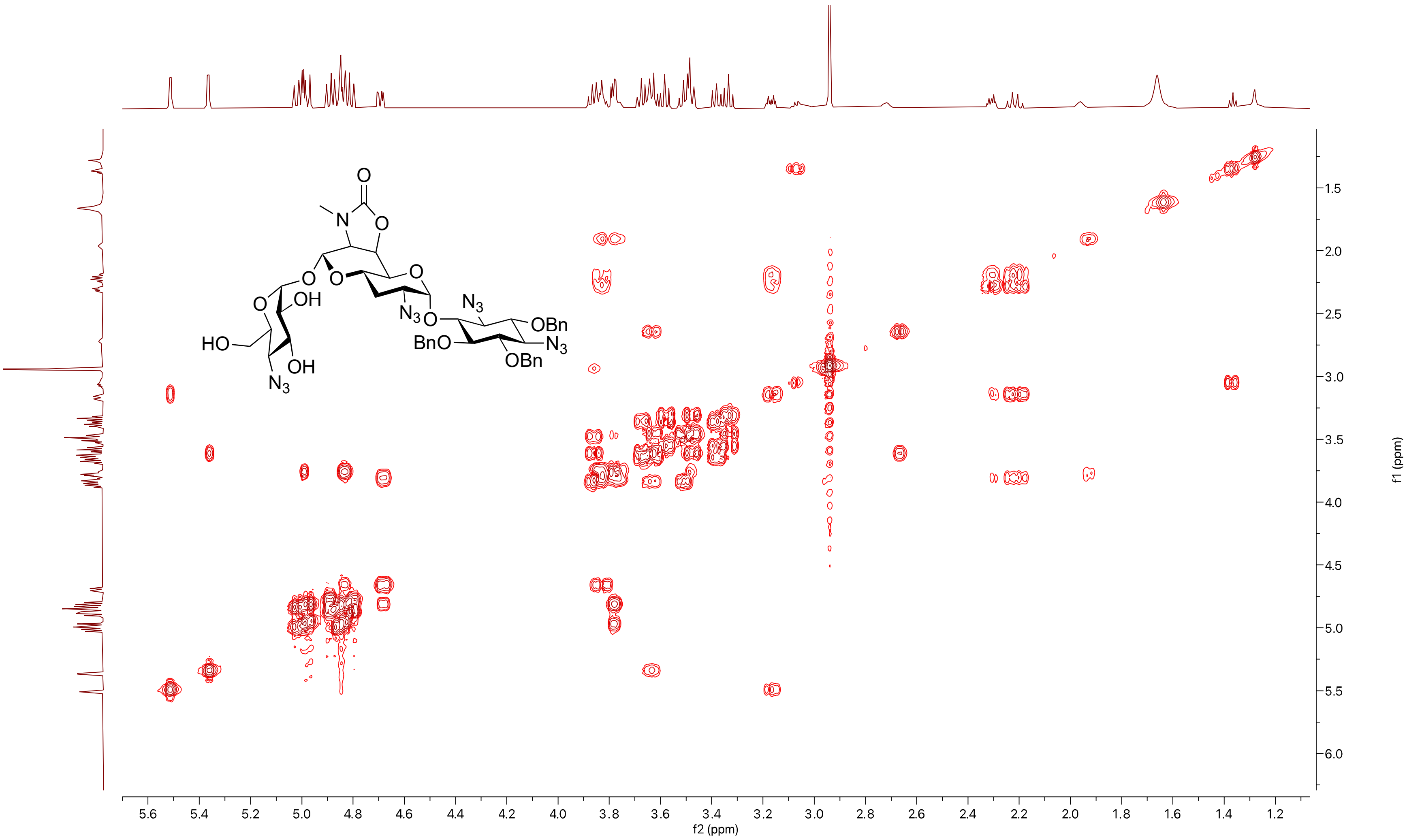

HSQC Spectrum (600 MHz, $CDCl_3$) of 1,3,2′,4″-Tetra-azido-2,5,6-tri-*O*-benzyl-1,3,2′,4″-tetra-(desamino)-6′,7′-oxazolidino-2-hydroxy-apramycin (**34**)

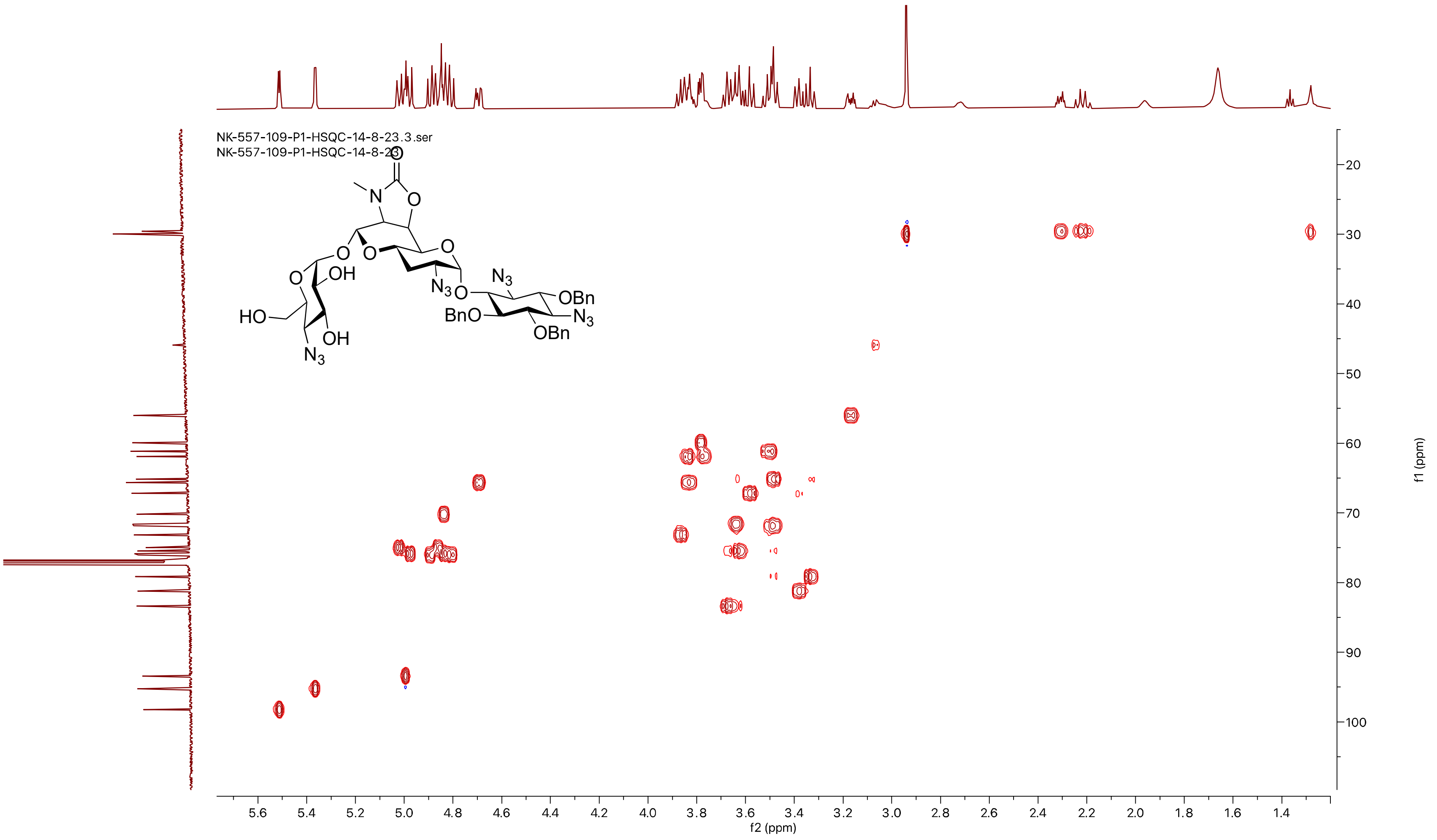

^{1}H NMR Spectrum (500 MHz, D_2O) of 1'-epi-2-Hydroxy-apramycin pentaacetate salt (**35**)

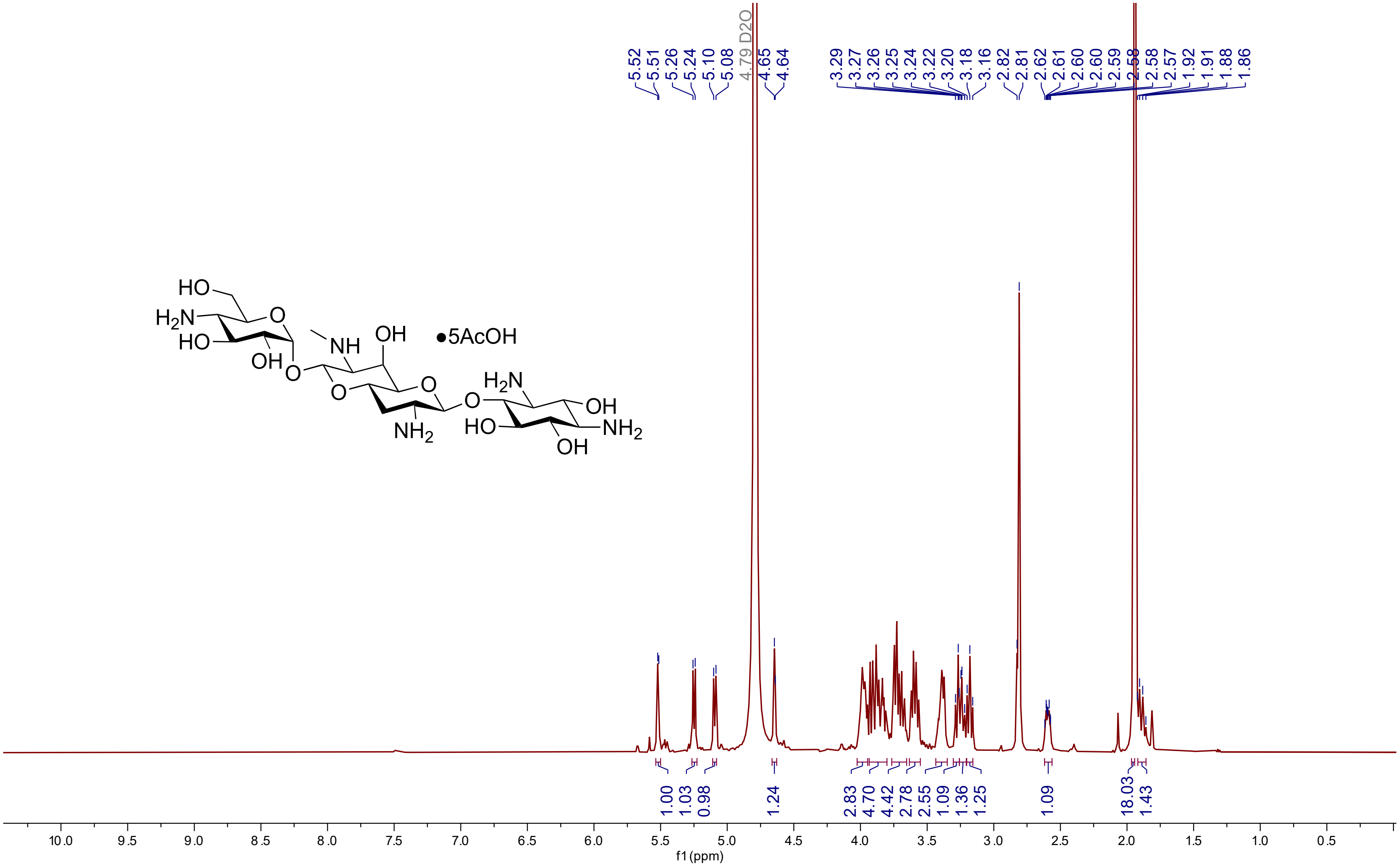

^{13}C NMR Spectrum (125 MHz, D_2O) of 1'-epi-2-Hydroxy-apramycin pentaacetate salt (**35**)

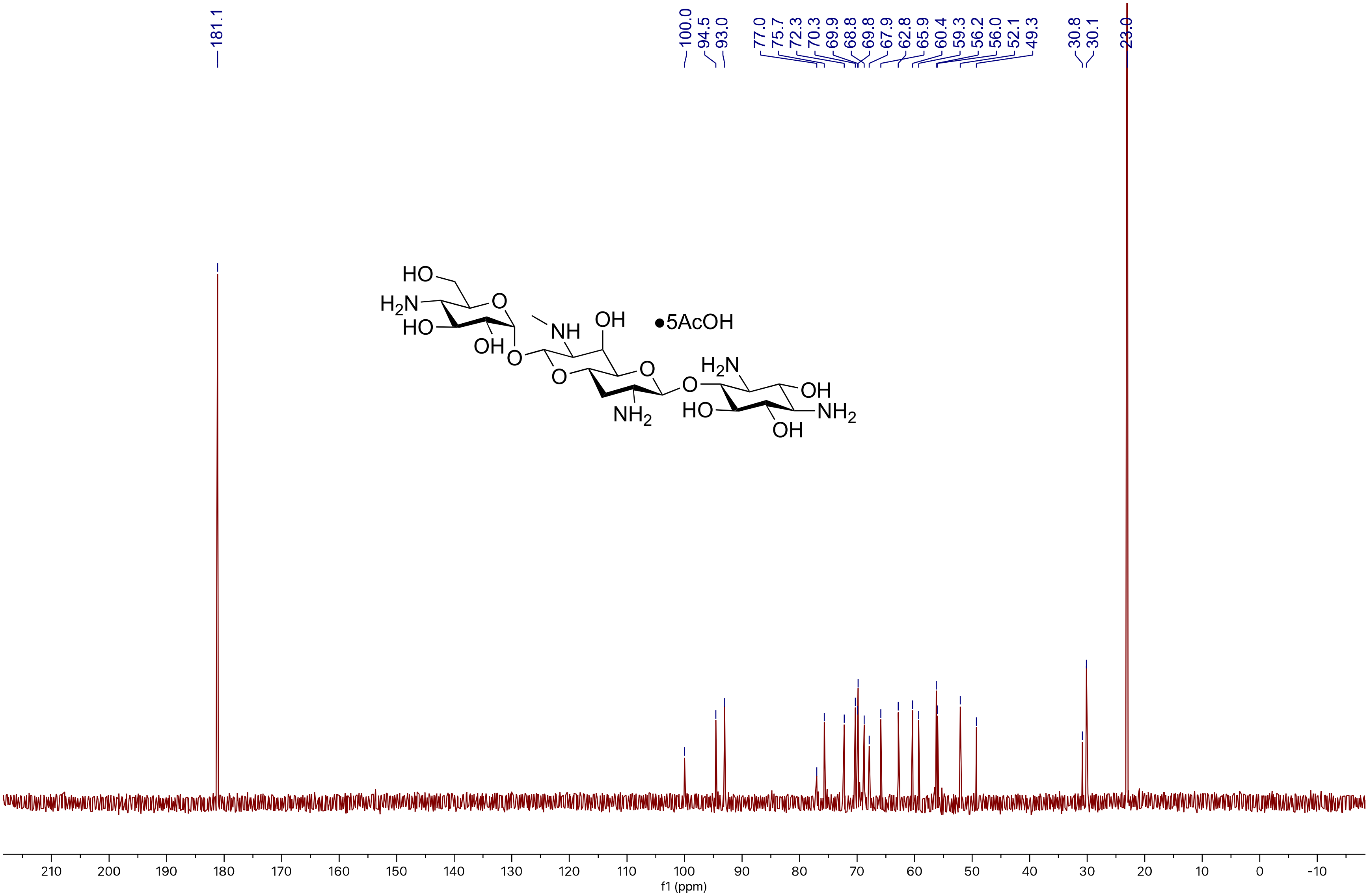

^1H-^1H COSY Spectrum (500 MHz, D_2O) of 1'-epi-2-Hydroxy-apramycin pentaacetate salt (**35**)

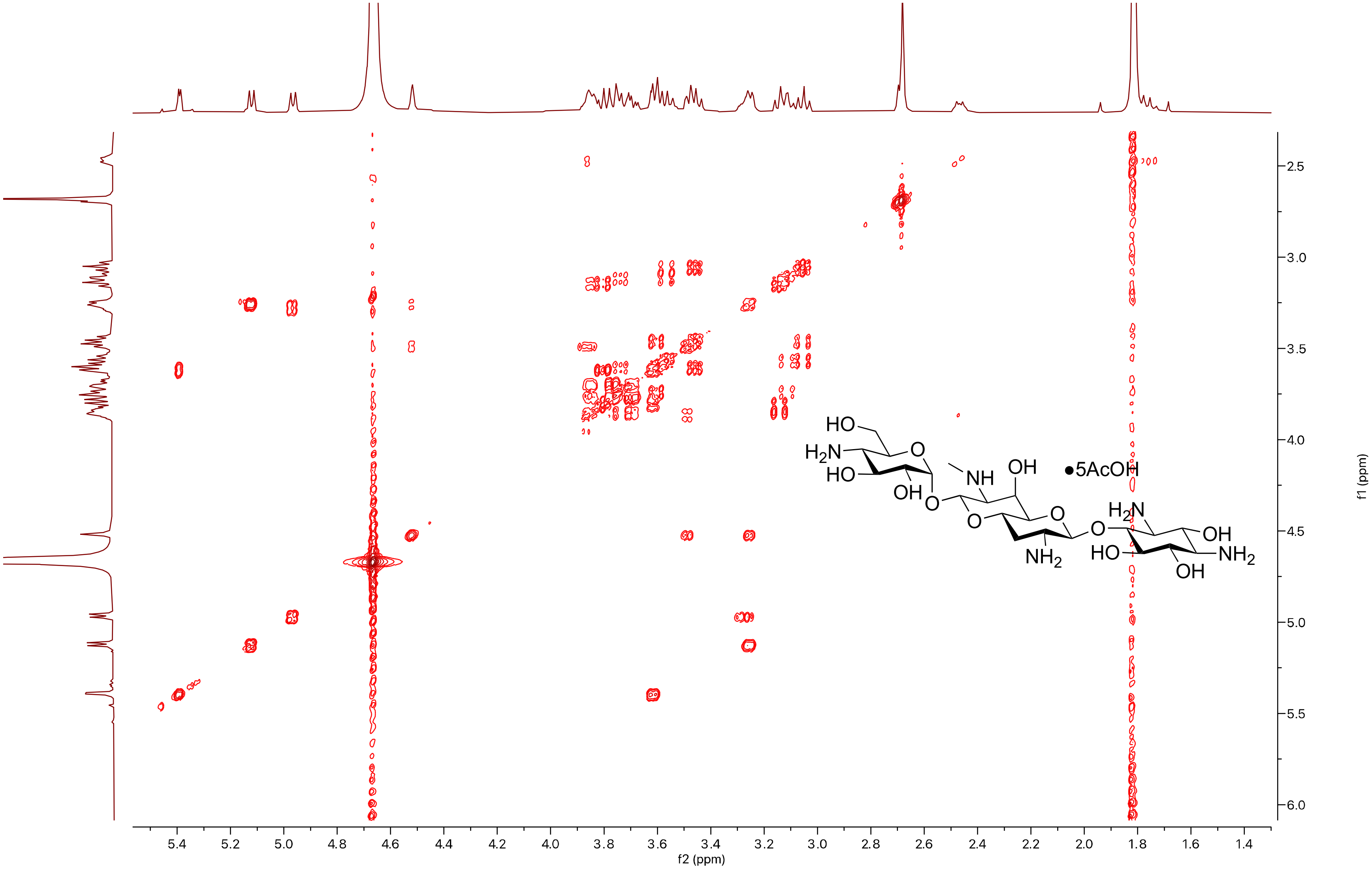

HSQC Spectrum (500 MHz, D_2O) of 1'-epi-2-Hydroxy-apramycin pentaacetate salt (**35**)

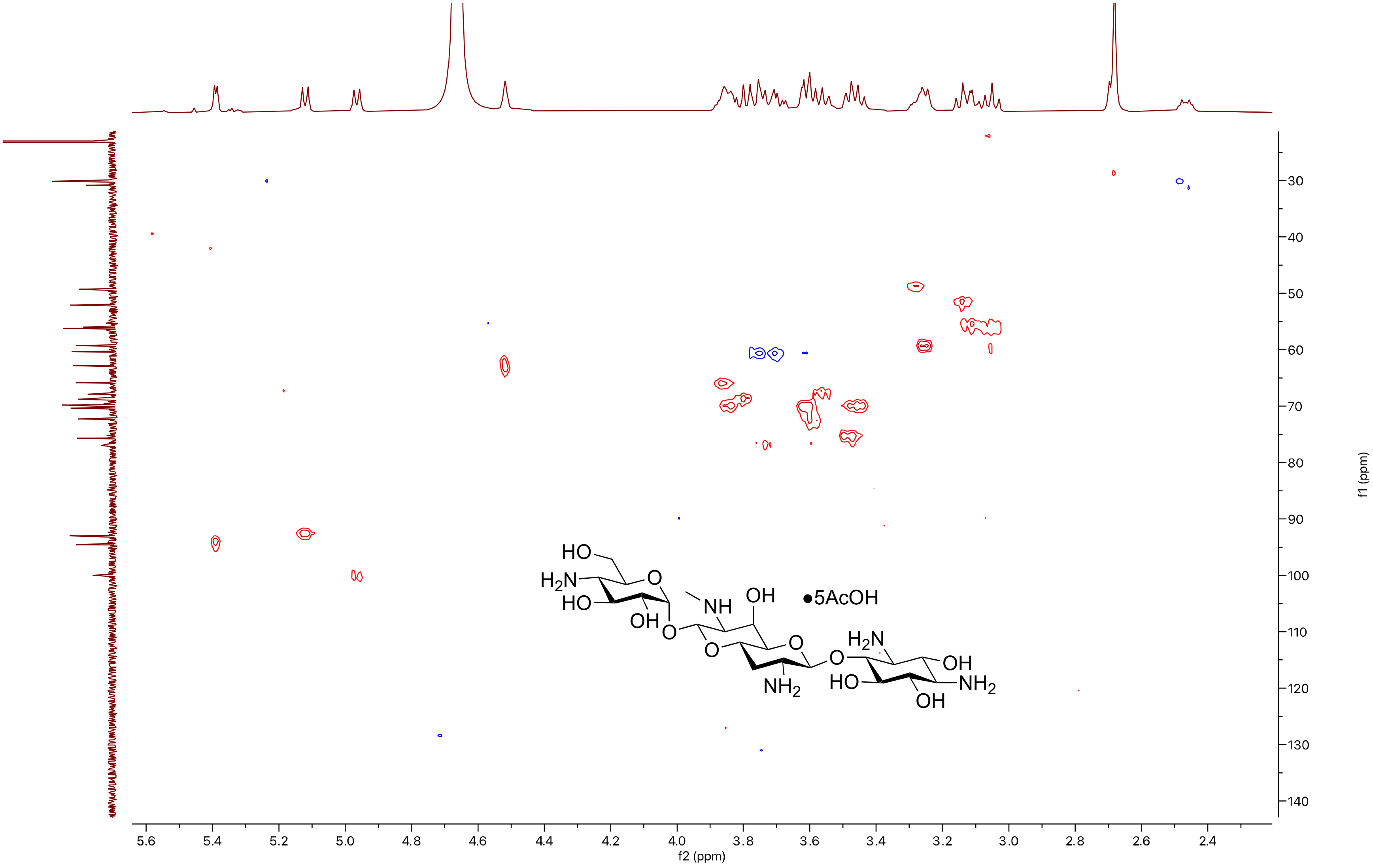

^{1}H NMR Spectrum (500 MHz, D_2O) of 2-Hydroxy-apramycin pentaacetate salt (**2**)

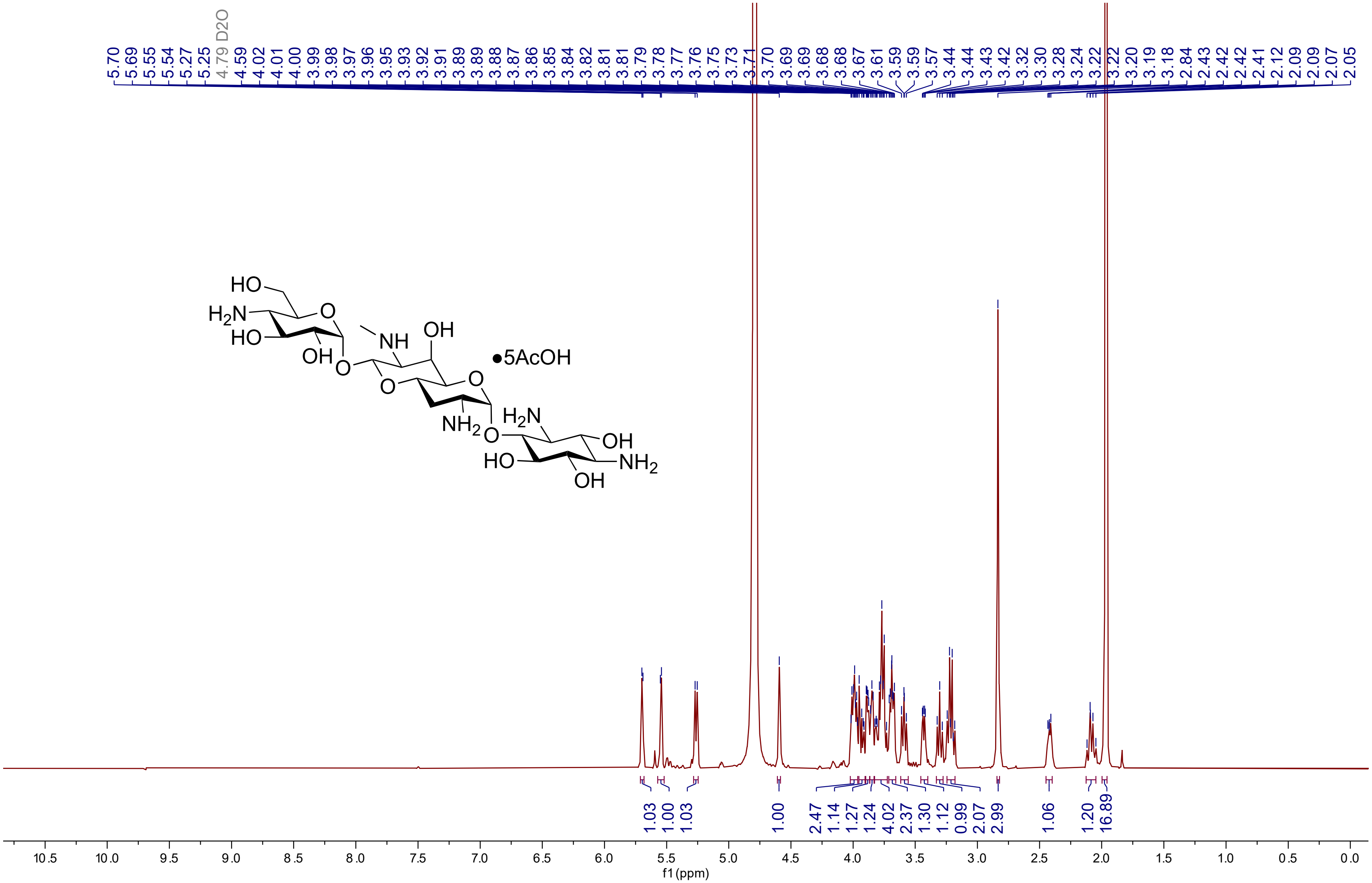

^{13}C NMR Spectrum (125 MHz, D_2O) of 2-Hydroxy-apramycin pentaacetate salt (**2**)

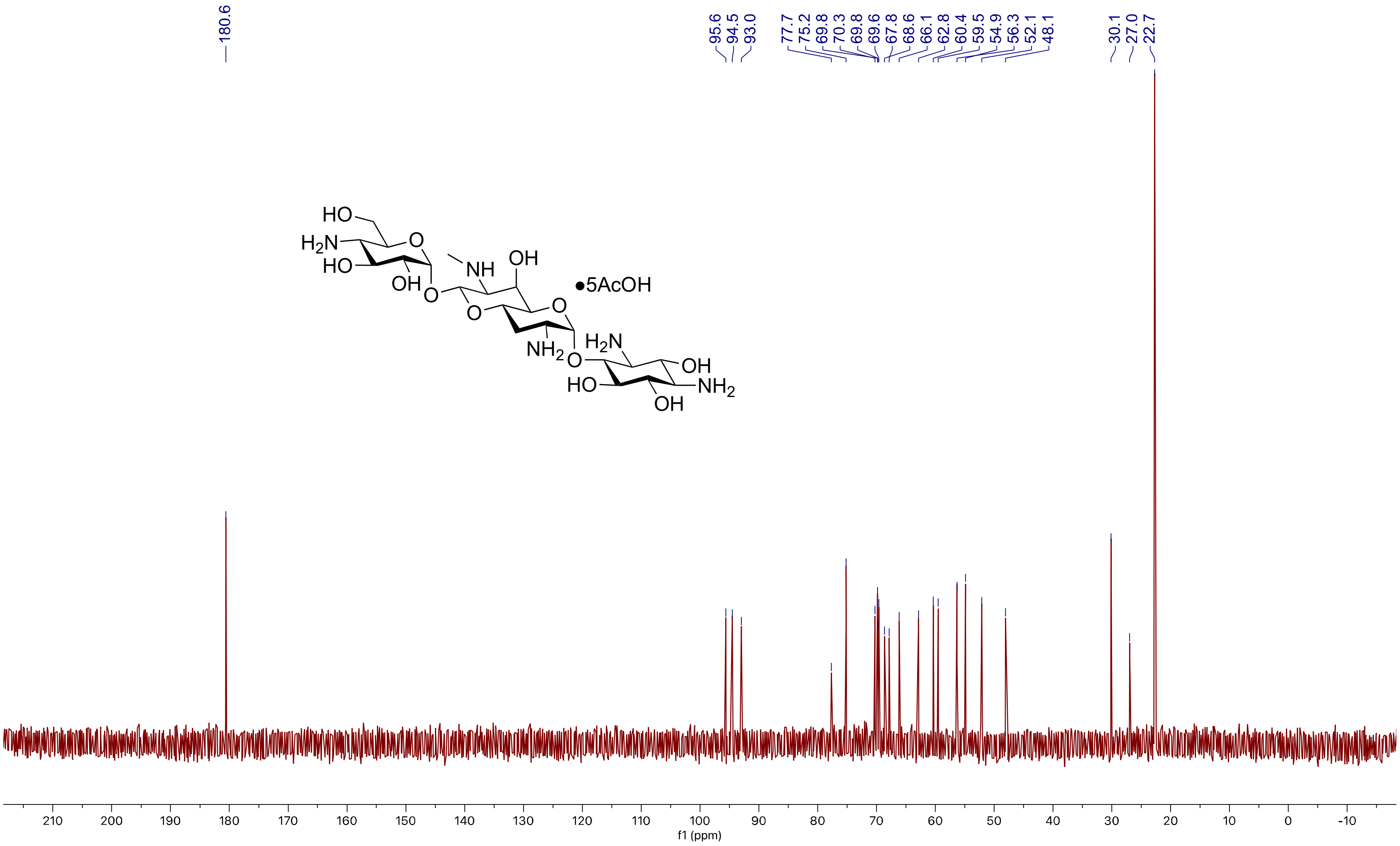

^1H-^1H COSY Spectrum (500 MHz, D_2O) of 2-Hydroxy-apramycin pentaacetate salt (**2**)

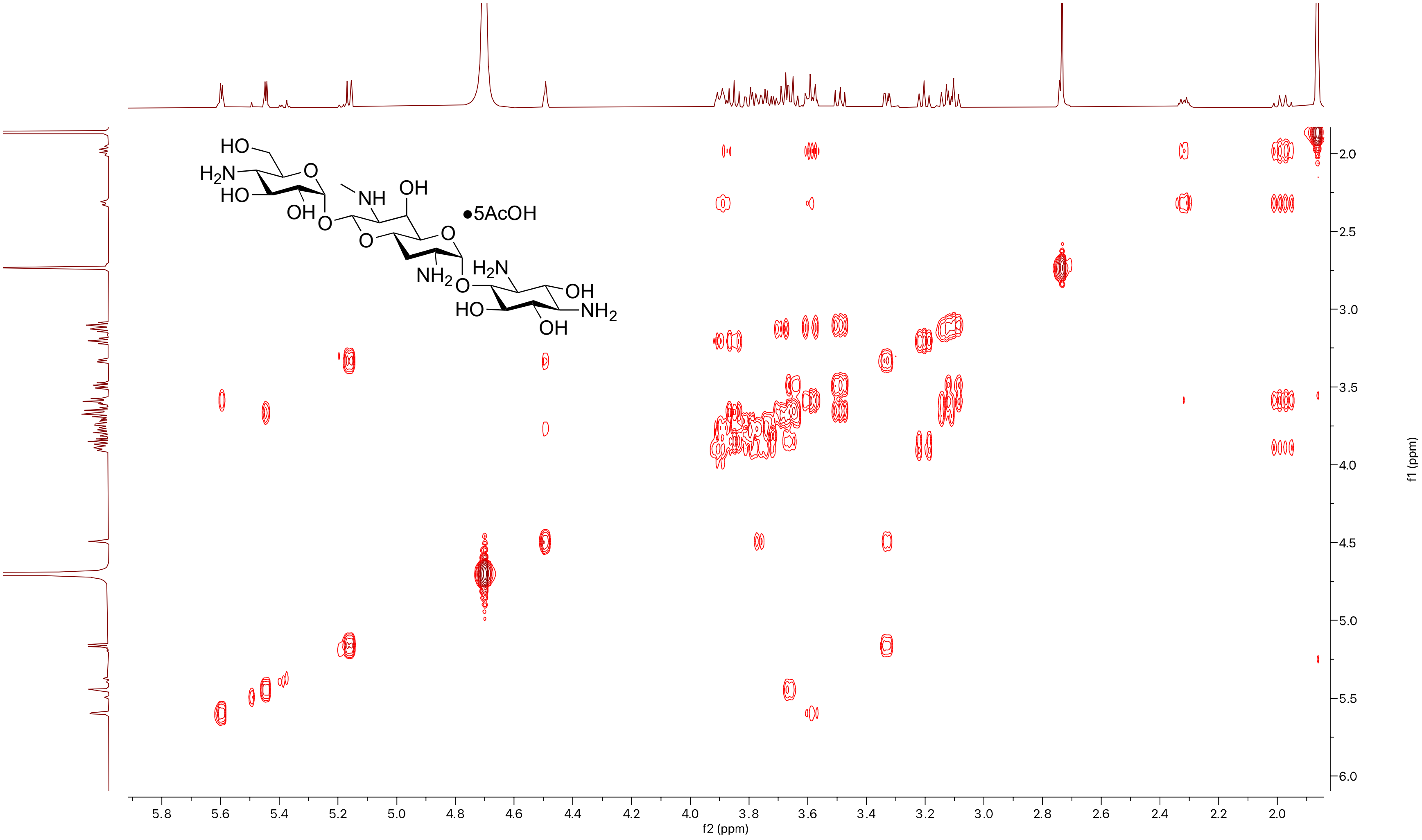

HSQC Spectra (500 MHz, D_2O) of compound 2-Hydroxy-apramycin pentaacetate salt (**2**)

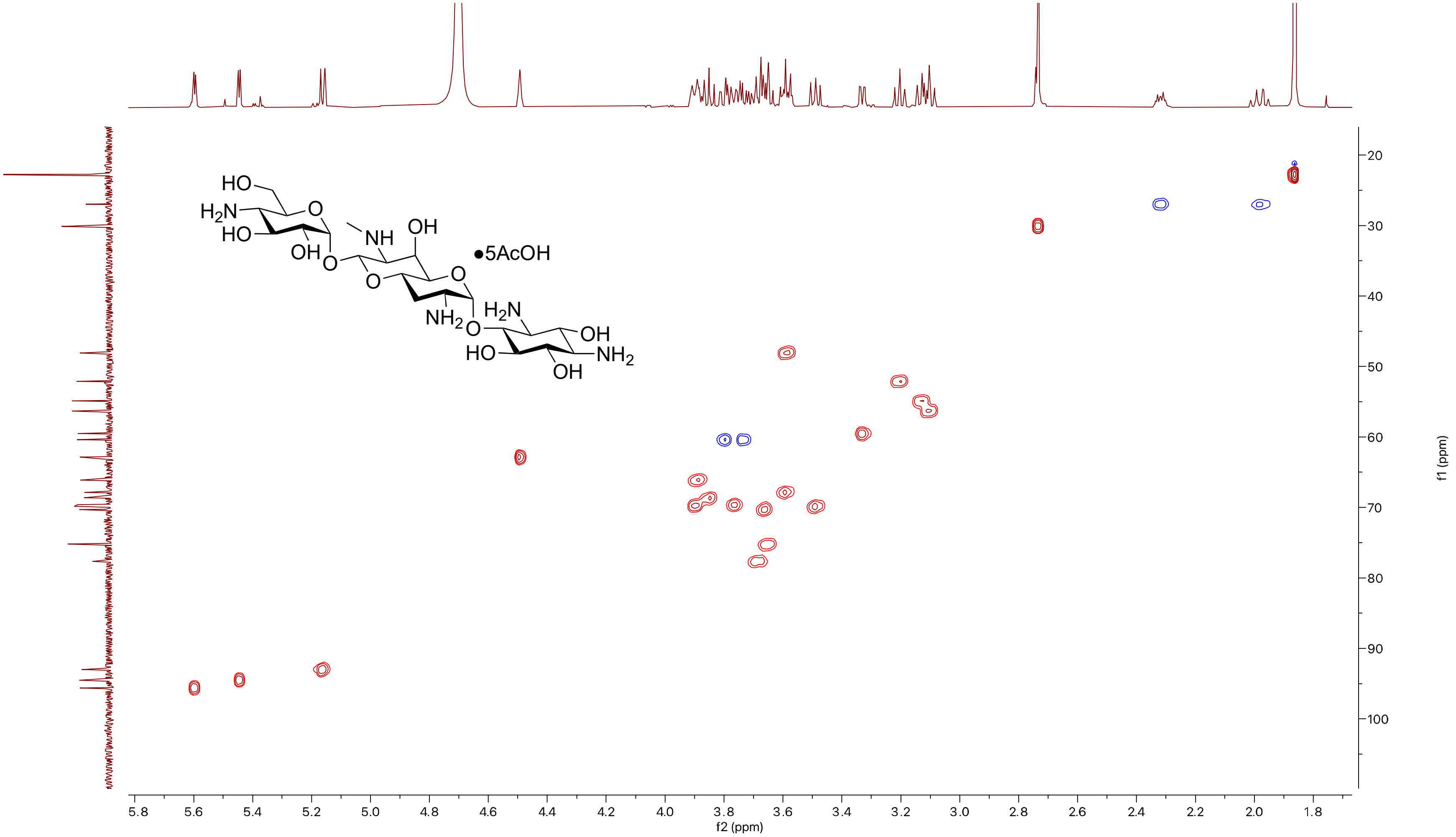

^{1}H NMR Spectrum (600 MHz, $CDCl_3$) of Methyl 2,4′-di-azido-2′,3′,6′-tri-*O*-benzoyl-2,4′-di-(desamino)-6′,7′-oxazolidino-α,β-aprabiosaminide (**36**)

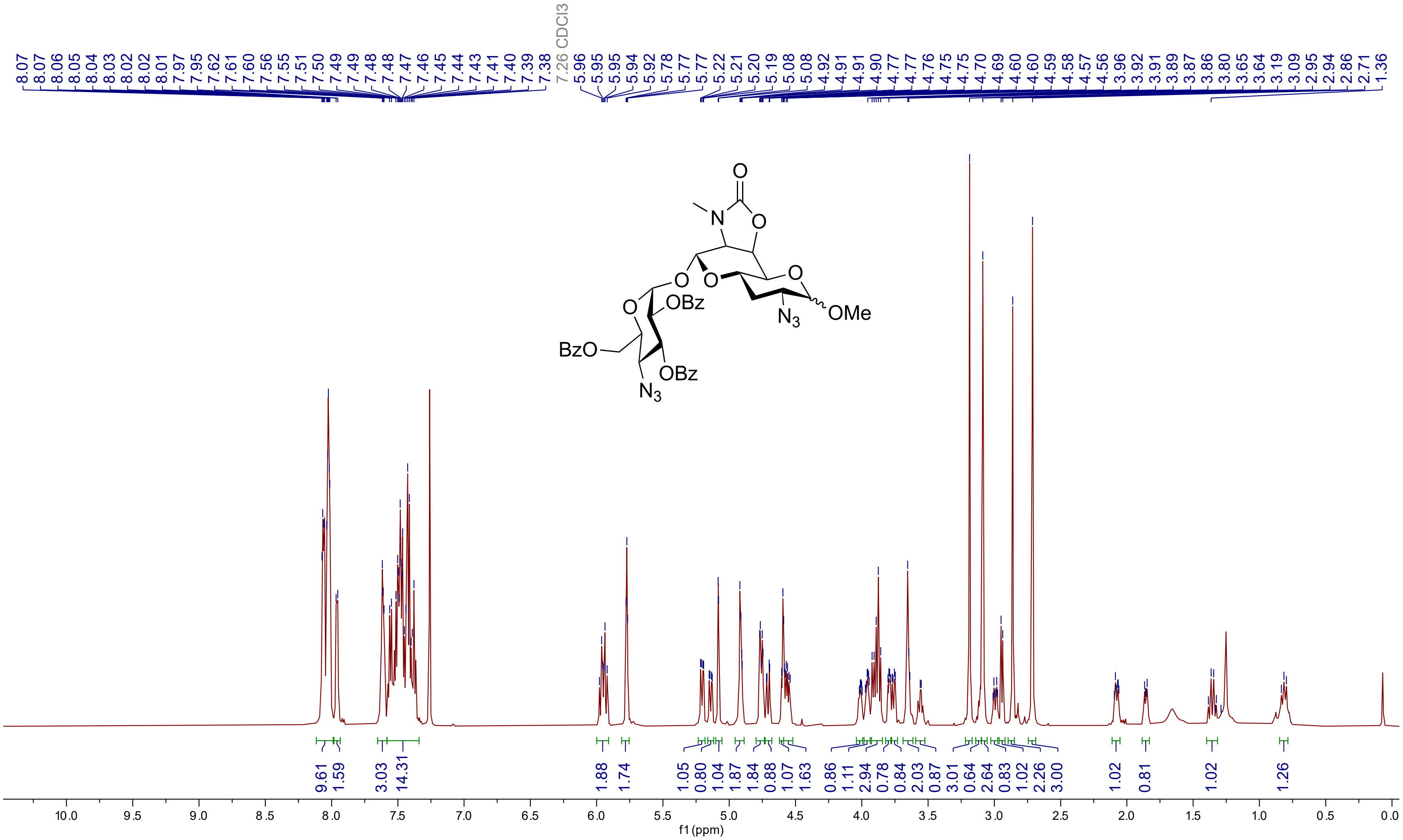

1D Selective gradient TOCSY (600 MHz, $CDCl_3$) of Methyl 2,4′-di-azido-2′,3′,6′-tri-*O*-benzoyl-2,4′-di-(desamino)-6′,7′-oxazolidino-α,β-aprabiosaminide (**36**)

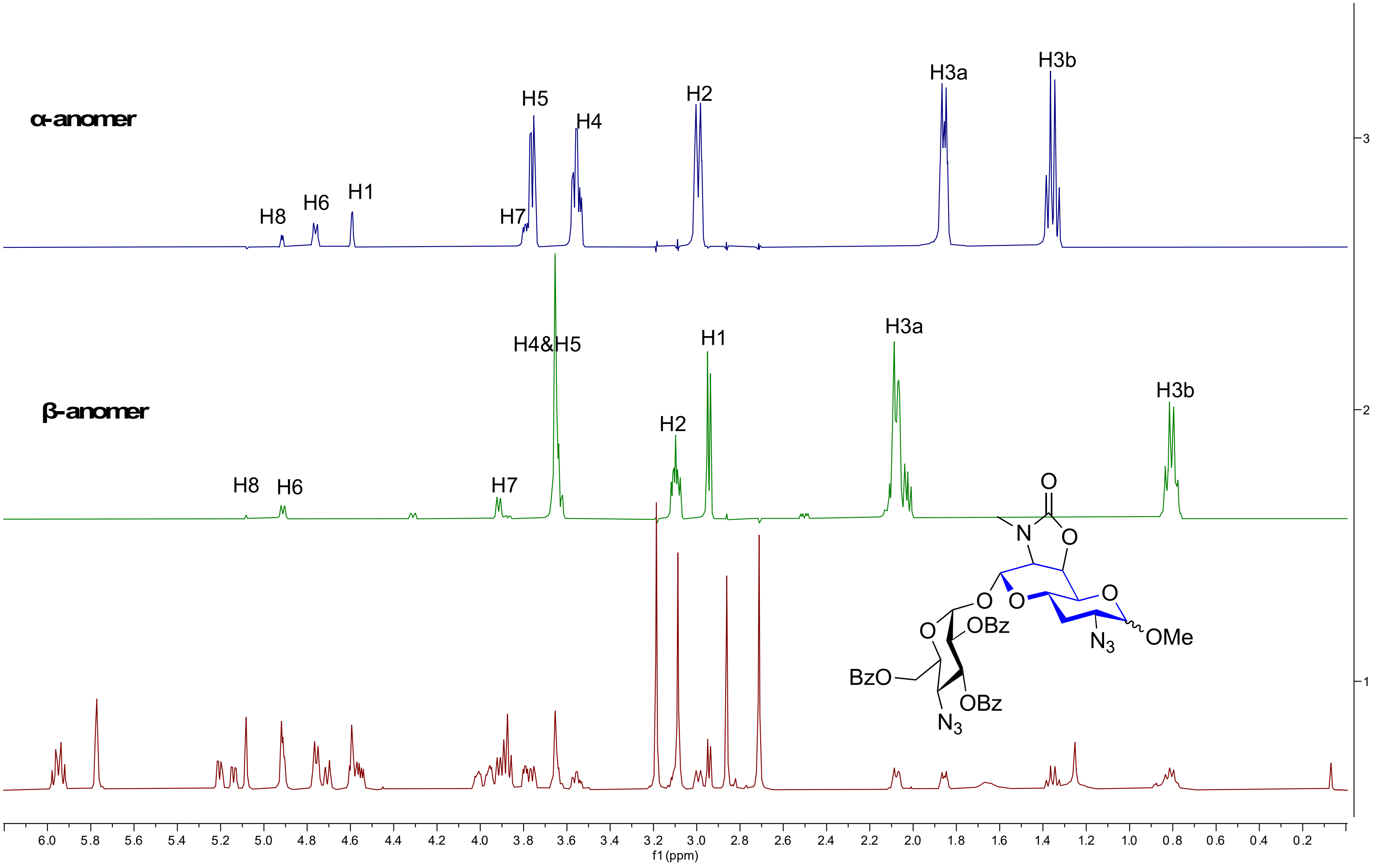

1D Selective gradient TOCSY (600 MHz, $CDCl_3$) of Methyl 2,4′-di-azido-2′,3′,6′-tri-*O*-benzoyl-2,4′-di-(desamino)-6′,7′-oxazolidino-α,β-aprabiosaminide (**36**)

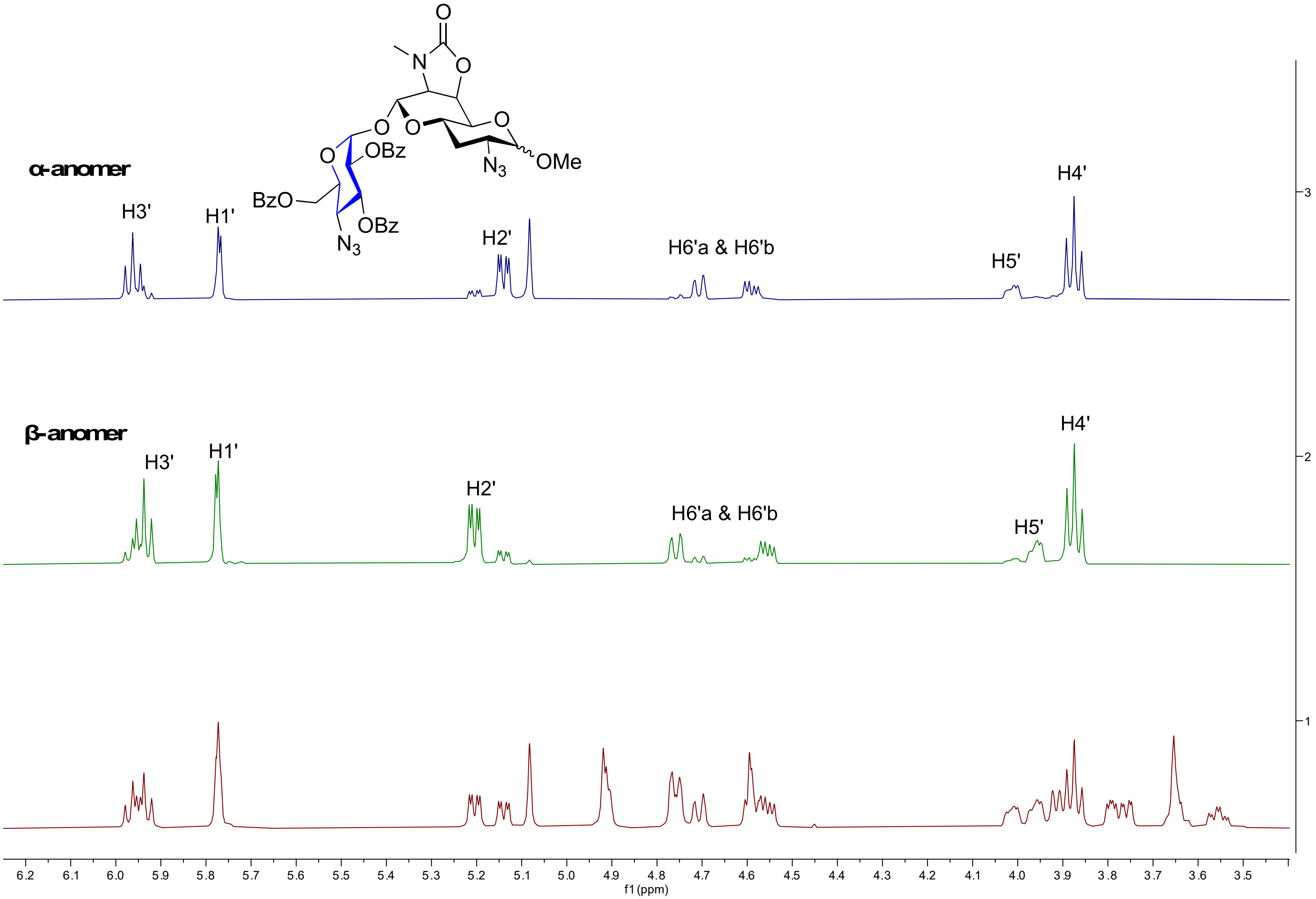

^{13}C NMR Spectrum (150 MHz, $CDCl_3$) of Methyl 2,4′-di-azido-2′,3′,6′-tri-*O*-benzoyl-2,4′-di-(desamino)-6′,7′-oxazolidino-α,β-aprabiosaminide (**36**)

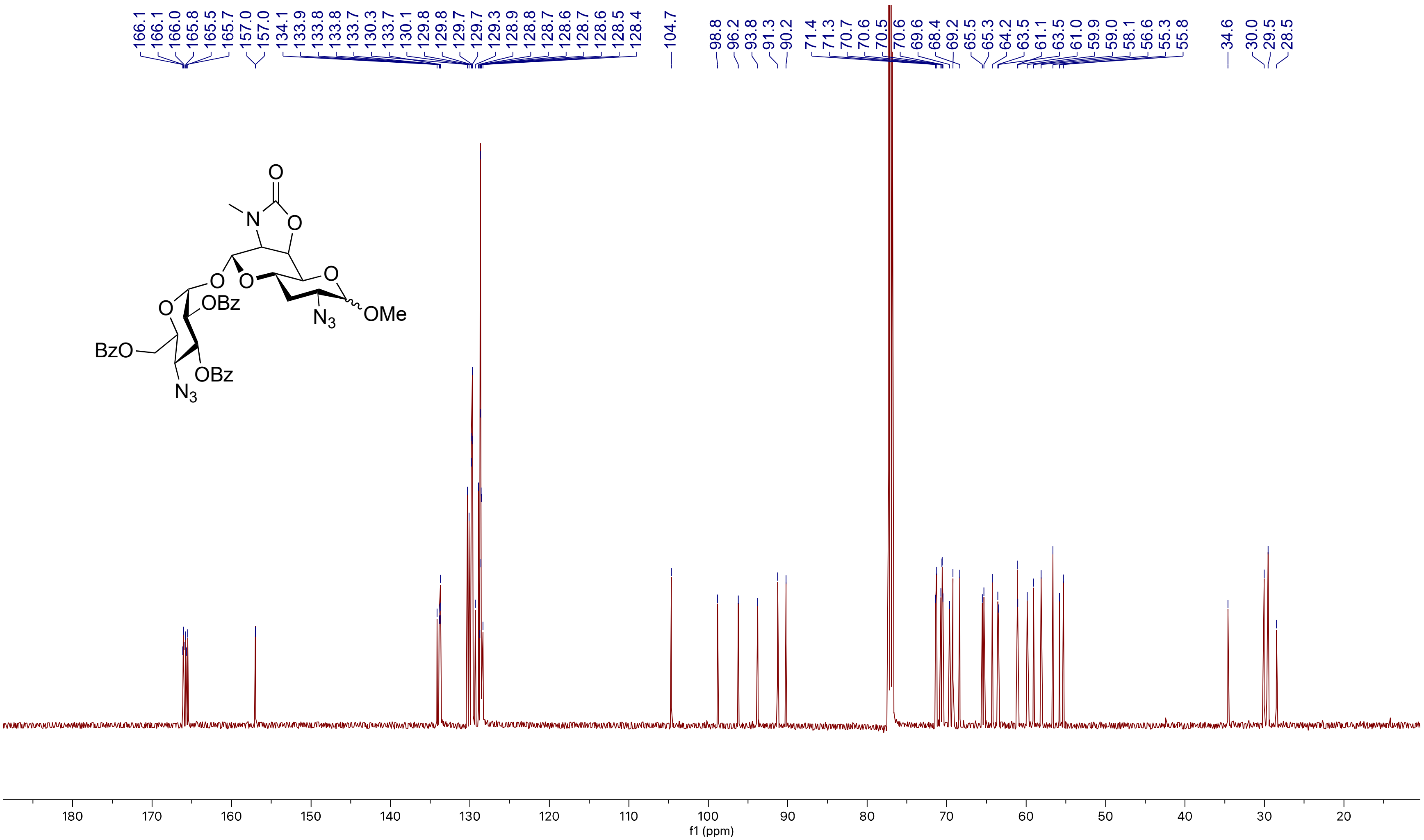

^{1}H-^{1}H COSY Spectrum (600 MHz, $CDCl_3$) of Methyl 2,4′-di-azido-2′,3′,6′-tri-*O*-benzoyl-2,4′-di-(desamino)-6′,7′-oxazolidino-α,β-aprabiosaminide (**36**)

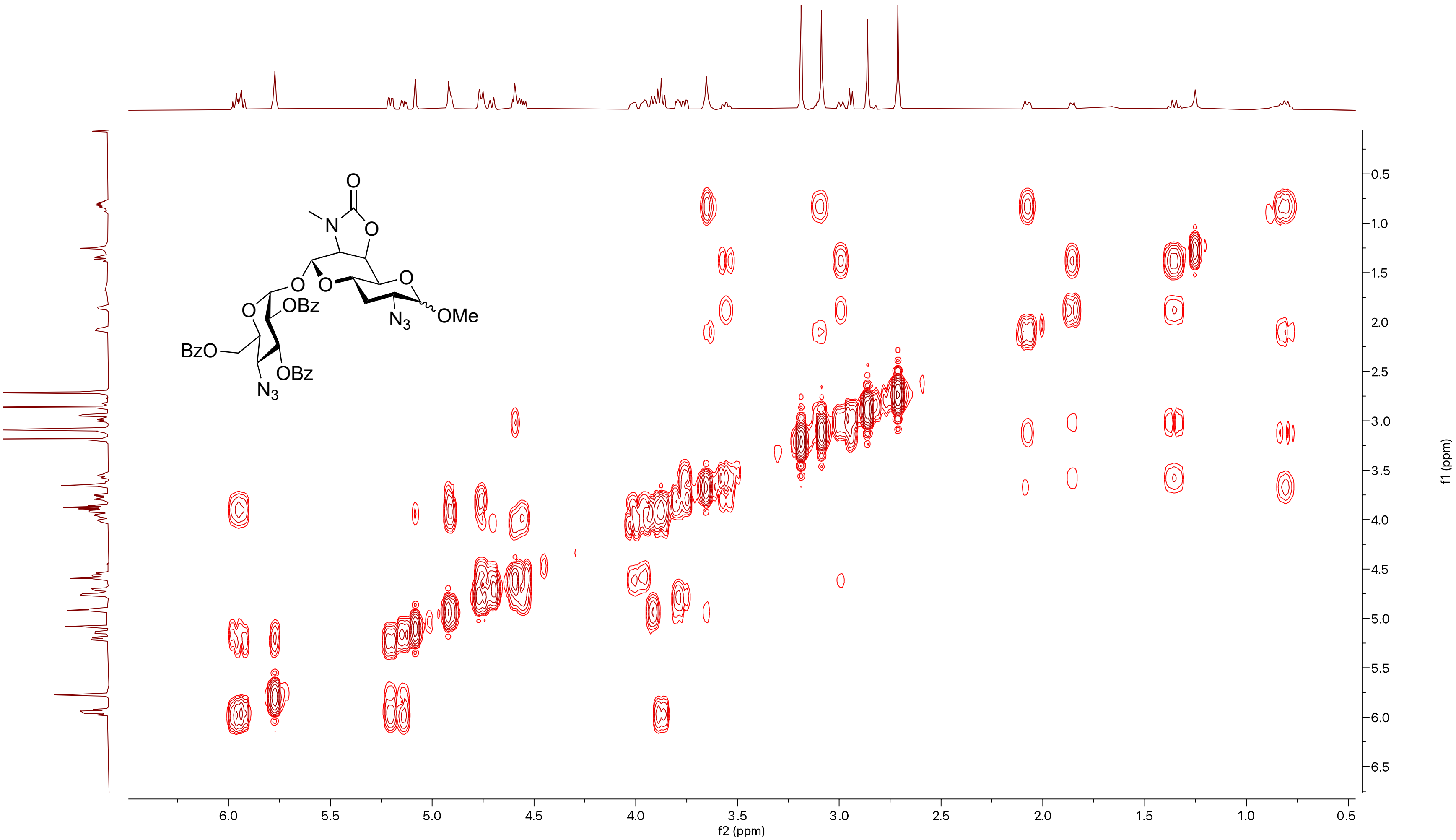

HSQC Spectrum (600 MHz, $CDCl_3$) of Methyl 2,4′-di-azido-2′,3′,6′-tri-*O*-benzoyl-2,4′-di-(desamino)-6′,7′-oxazolidino-α,β-aprabiosaminide (**36**)

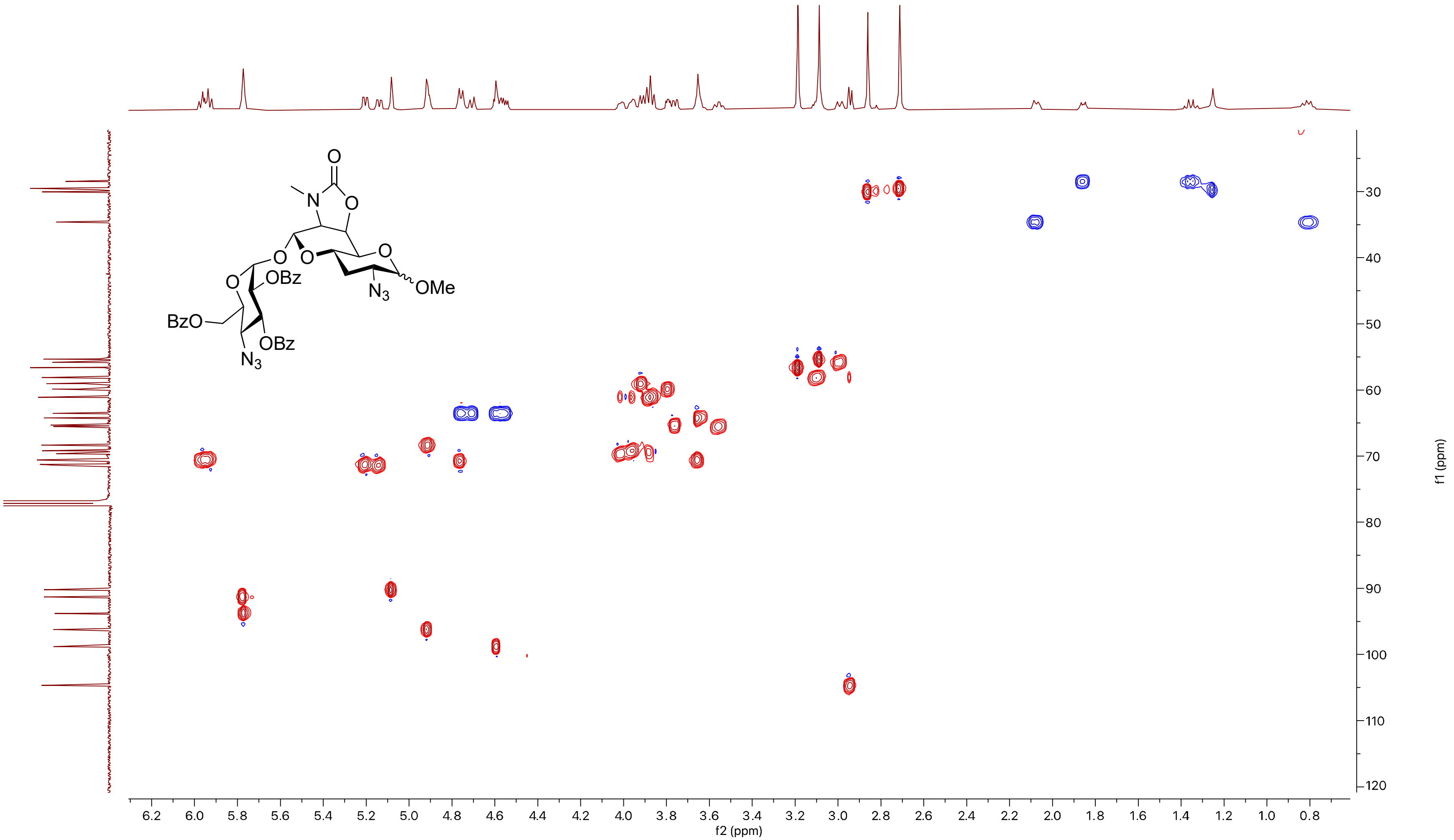

^{1}H NMR Spectrum (500 MHz, D_2O) of Methyl α,β-aprabiosaminide triacetate salt (**37**)

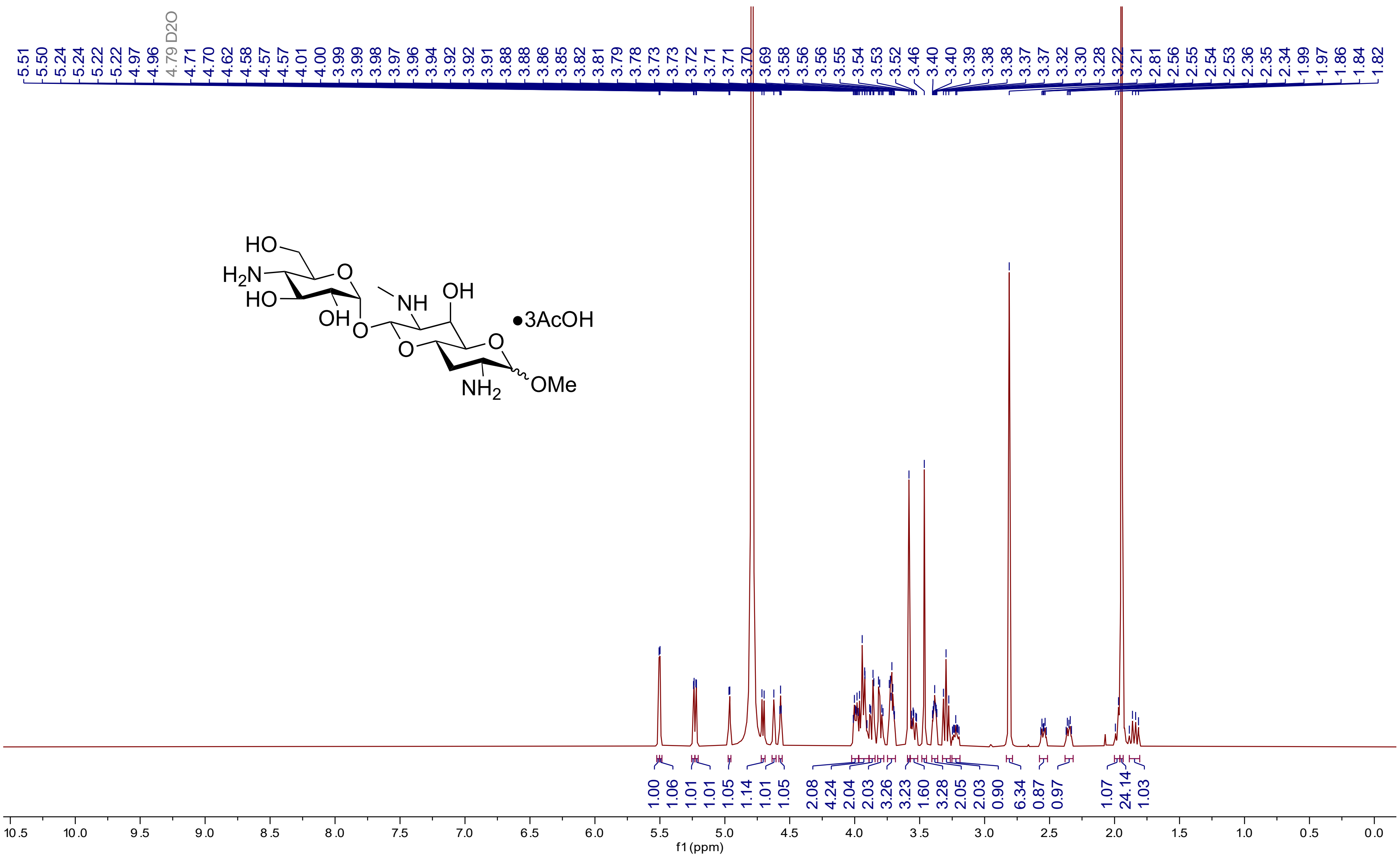

1D Selective gradient TOCSY (600 MHz, D_2O) of Methyl α,β-aprabiosaminide triacetate salt (**37**)

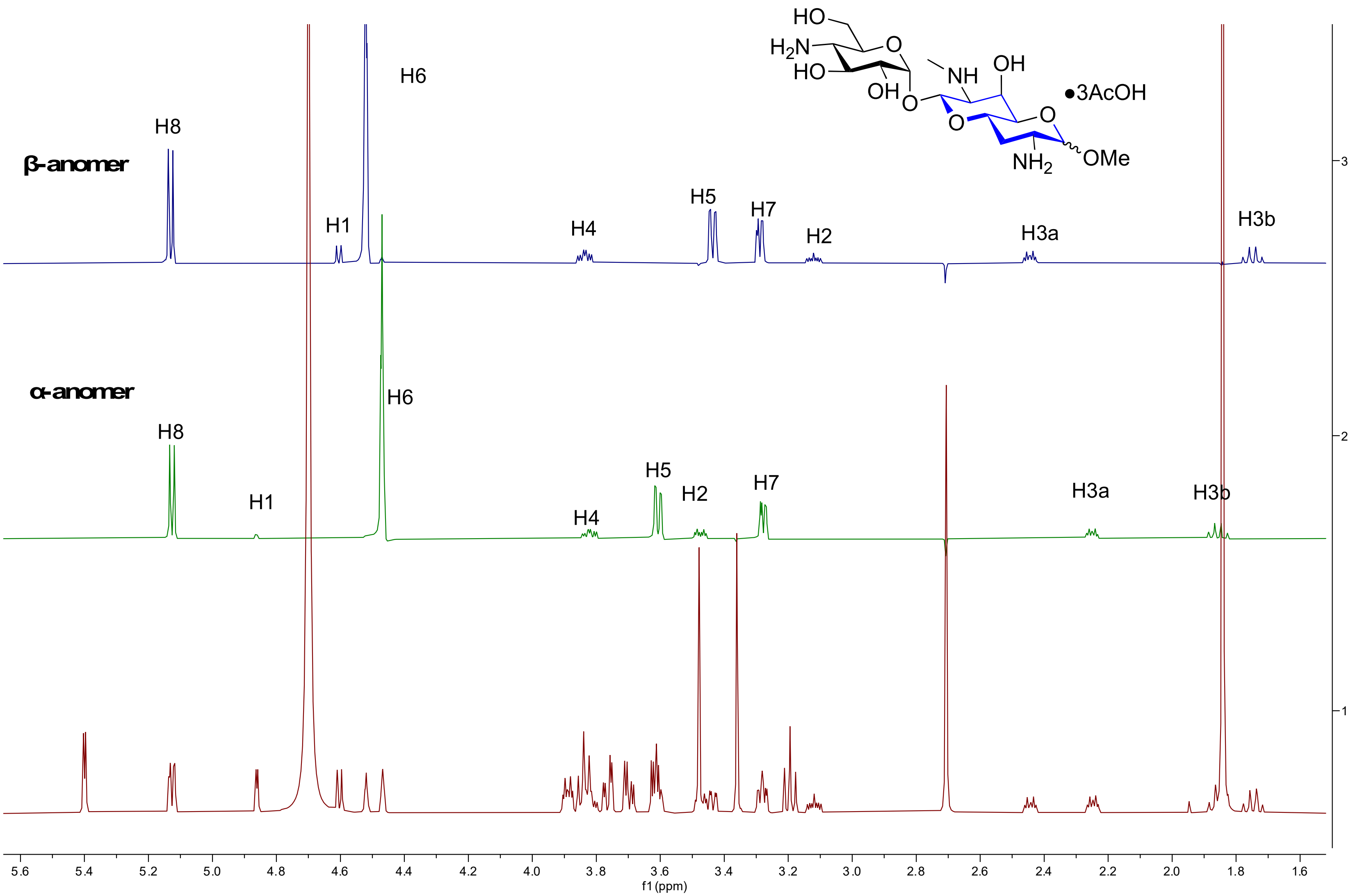

^{13}C NMR Spectrum (500 MHz, D_2O) of Methyl α,β-aprabiosaminide triacetate salt (**37**)

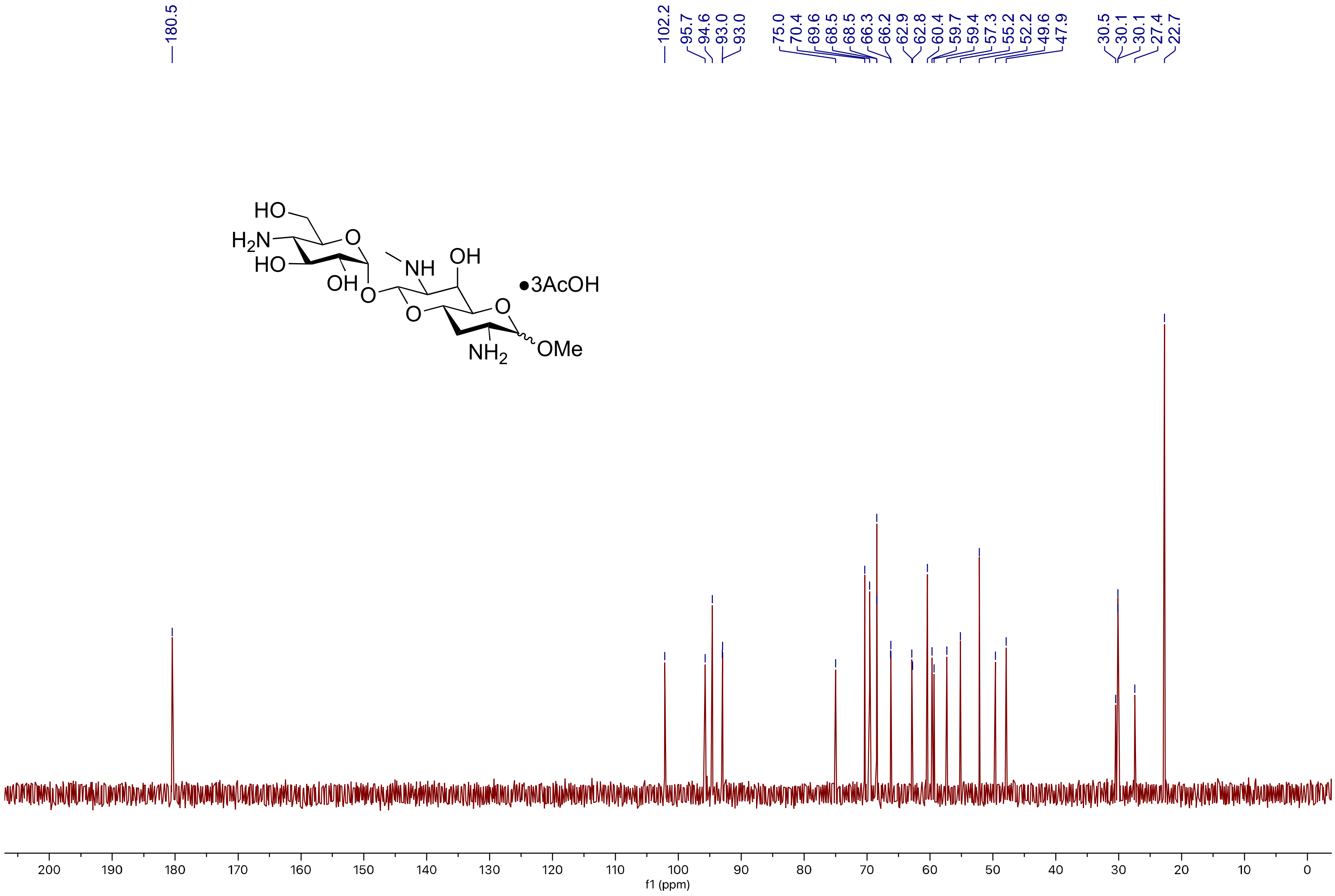

^{1}H-^{1}H COSY Spectrum (500 MHz, D_2O) of Methyl α,β-aprabiosaminide triacetate salt (**37**)

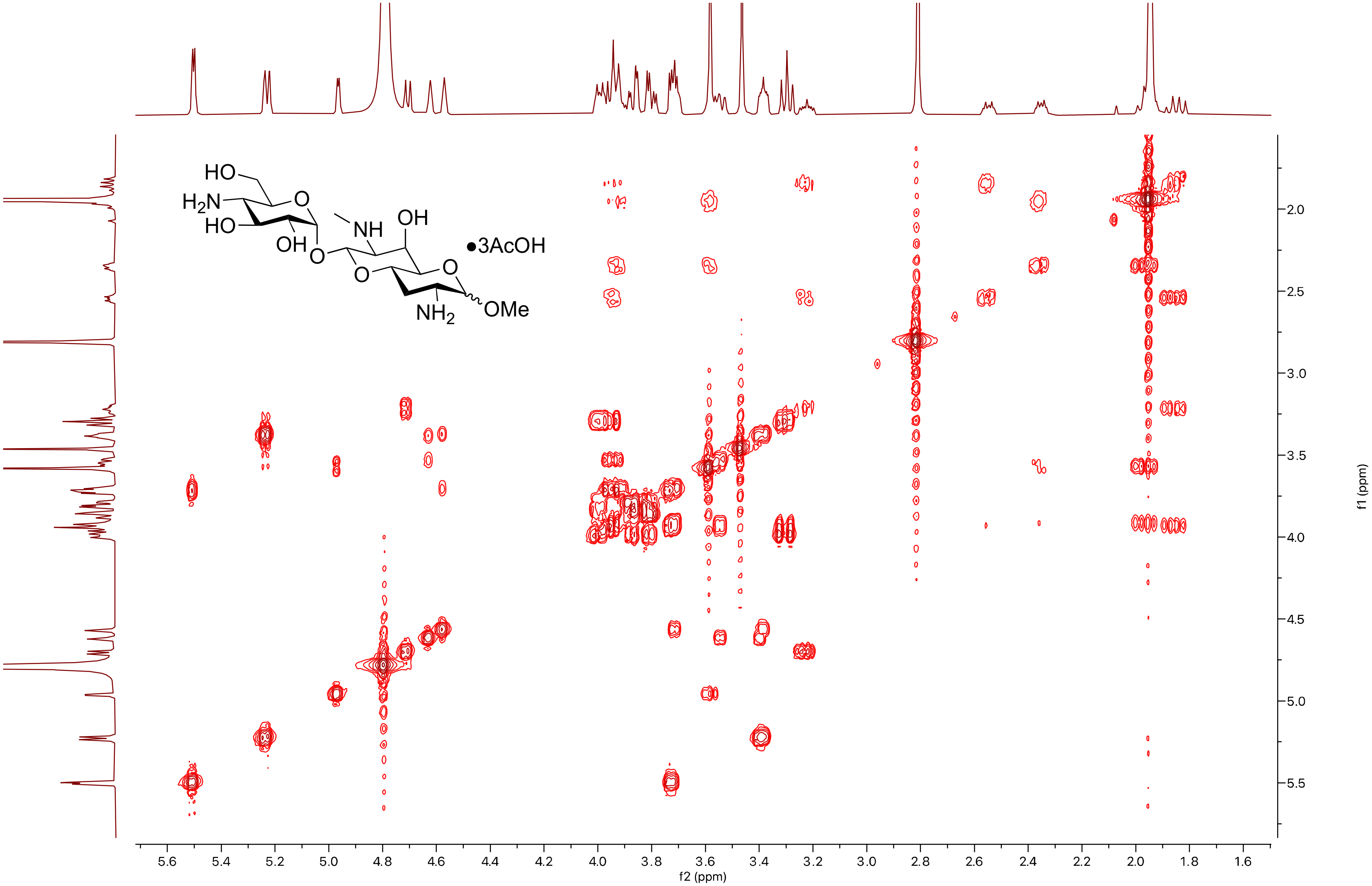

HSQC Spectrum (500 MHz, D_2O) of Methyl α,β-aprabiosaminide triacetate salt (**37**)

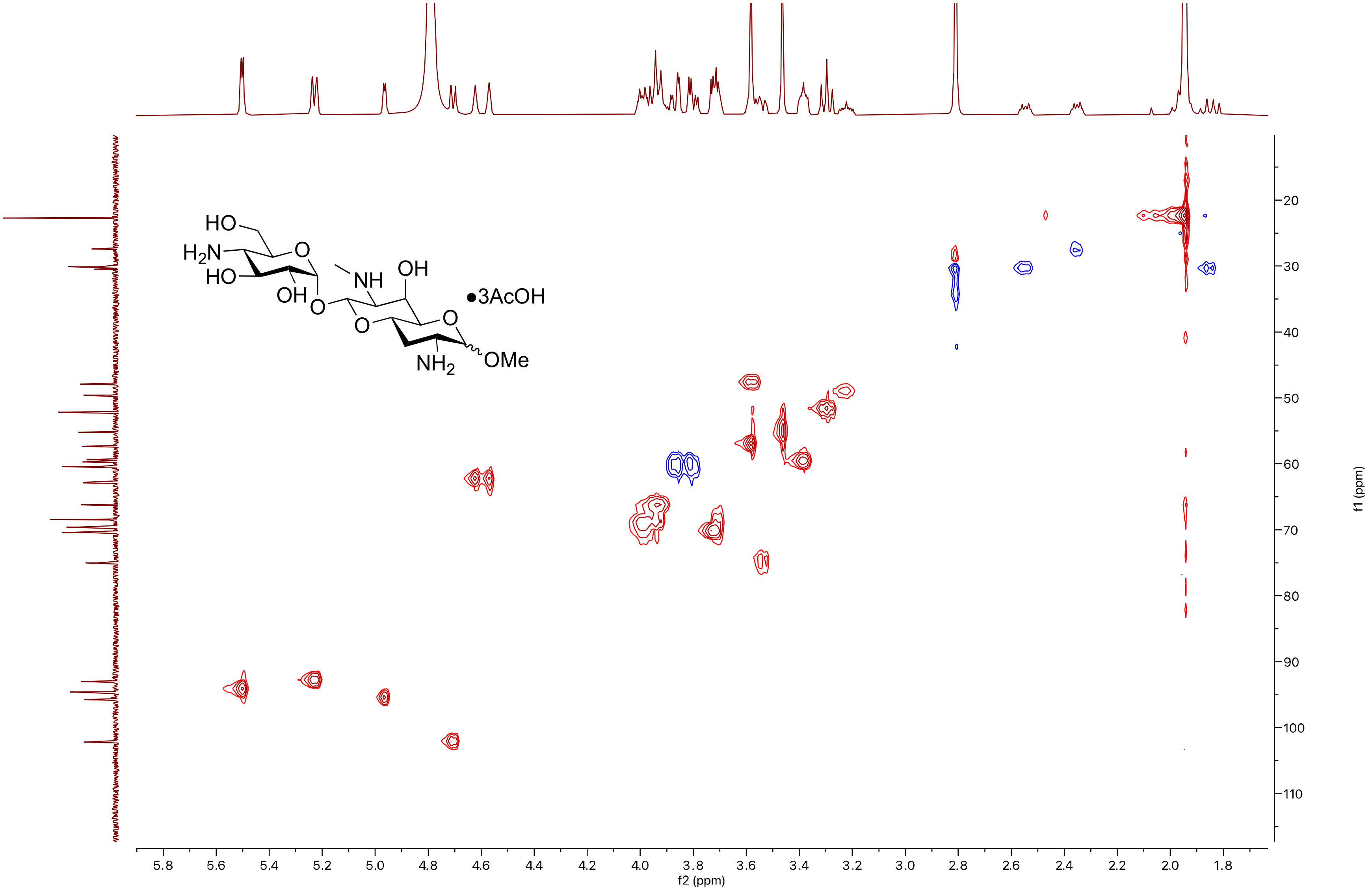

Supplement: Supplementary file 1 — ol5c00168_si_001.pdf [file ol5c00168_si_001.pdf]
